# Microfluidics-Based Ionic Catch and Release Oligosaccharide Synthesis (ICROS-Microflow) to Expedite Glycosylation Chemistry

**DOI:** 10.1021/jacsau.4c00686

**Published:** 2024-10-21

**Authors:** Yao-Yao Zhang, Mattia Ghirardello, Ryan Williams, Adrian Silva Diaz, Javier Rojo, Josef Voglmeir, Javier Ramos-Soriano, M. Carmen Galan

**Affiliations:** † School of Chemistry, Cantock’s Close, University of Bristol, Bristol BS8 1TS, U.K.; ‡ Instituto de Investigaciones Químicas, CSIC—Universidad de Sevilla, Avenue Américo Vespucio 49, Seville 41092, Spain; § Glycomics and Glycan Bioengineering Research Center, College of Food Science and Technology, Nanjing Agricultural University, 1 Weigang, 210095 Nanjing, China

**Keywords:** ICROS, ITag, continuous flow, glycosylation, oligosaccharide synthesis

## Abstract

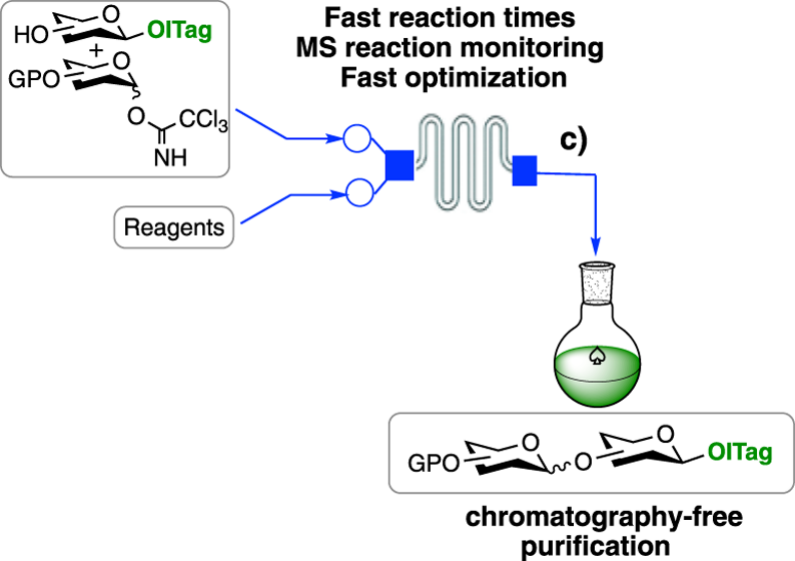

A continuous microfluidic glycosylation strategy that requires no chromatography between steps and significantly expedites glycosylation chemistry is described. This practical approach incorporates the advantages of imidazolium-based chromatography-free purification and in situ mass spectrometry reaction monitoring to achieve fast reaction optimization and shorter reaction times. We demonstrate the utility of this strategy in the synthesis of a series of glycoside targets, including an α-(1,6)-trimannoside and a branched Man_5_ oligomannoside, within 1 day.

Carbohydrates are one of the most abundant biopolymers on Earth and are key components of all living organisms. Complex oligosaccharides are involved in a myriad of biological processes, from cell recognition, immune response modulation, to signal transduction.^1^ Access to diverse and structurally defined glycan libraries to study such processes is essential for the advancement of glycobiology and glycomedicine research. The synthesis of oligosaccharides is a complex and challenging task due to the structural diversity and stereochemical complexity of these molecules. Significant progress has been made in the development of automated oligosaccharide synthesis methods, enabling more efficient and streamlined access to these valuable biomolecules.^2−4^ To this end, polymer-supported oligosaccharide syntheses have shown great promise for the purpose of automation.^2,4^ However, issues such as incomplete conversion, insufficient control of reaction rates, and the need to control the stereoselectivity of the glycosylation reaction for each given target are more difficult to manage on a solid support than in solution phase. Moreover, most automated systems rely on expensive equipment, e.g., Glyconeer,^2^ adapted HPLC,^5^ or peptide synthesizer systems,^6^ and often require specialized expertise.

As a solution-phase alternative, we previously reported an ionic-liquid-supported “catch-and-release” oligosaccharide synthesis (ICROS) strategy, where imidazolium-based purification labels (ITags) introduced at the anomeric position of the reducing end oligosaccharide target are used as a soluble functional support to facilitate chromatography-free purification by simple biphasic extractions.^7^ Moreover, the permanent positive charge of ITags provides the labeled molecules with exceptional mass spectrometry (MS) low limit of detection^8−10^ and thus allows in situ reaction progress monitoring by MS in addition to HPLC and NMR analysis, offering great advantage over other traditional supported methodologies.^9,11,12^ The methodology was shown to be compatible with both chemical and enzymatic processes;^9,10,12−14^ however, chemical reactions in batch in the presence of ITagged-glycosyl acceptors were often slow (between 1 and 16 h), making the approach less effective.^9,12^

Continuous flow strategies allow large-scale production^15^ and increased reaction efficiencies^16^ as compared to batch processes and have been implemented in organic chemistry for the efficient synthesis of complex natural products,^17^ drug molecules,^18^ including examples in carbohydrate chemistry.^15,16,19−21^ Microfluidic-based devices featuring submillimeter reaction channels can perform a wide range of single and multiphase organic reactions,^22^ allowing for the precise control of reaction variables such as flow rates, reagent mixing, reaction time, and heat and mass transfer. However, despite these advantages, reaction optimization, particularly in the context of glycosylation chemistry, is still time-consuming and requires significant amounts of starting materials, since analysis of intermediates and product isolation using conventional laborious approaches is needed after each step. The above issues ultimately hinder the speed at which multistep reactions can be optimized and streamlined to efficiently access the desired products.

While flow chemistry does not change the chemistry or kinetics of a reaction, these type of strategies can help eliminate or reduce concentration gradients that may be detrimental to reaction outcomes.^20^ Furthermore, microfluidic systems feature increased surface area to volume ratios due to the decreased size of the reactor; this is particularly important in multiphasic reaction systems, as in ICROS,^7^ where the interfacial area can play an important role in phase transfer of reaction components and can be rate limiting.^20^

On this basis, we envisioned that combining ICROS with microfluidic-based glycosylation strategies could address the current limitations and pave the way for solution-phase automated oligosaccharide synthesis. Herein, we describe the development of the ICROS-microflow strategy (Figure 1), which incorporates the advantages of ionic liquid-based chromatography-free purification and in situ MS reaction monitoring with continuous flow chemistry. This enables shorter reaction times with glycosylations completed within 15 s–2 min, excellent control of reaction conditions, and fast reaction optimization (e.g., reaction time/temperature/conversion rates). The strategy facilitates the expedient synthesis of a range of oligosaccharides such as a linear α-(1,6)trimannoside and a branched Man_5_ oligomannoside (a fragment of the relevant high-mannose oligosaccharide).^23^

**Figure 1:**
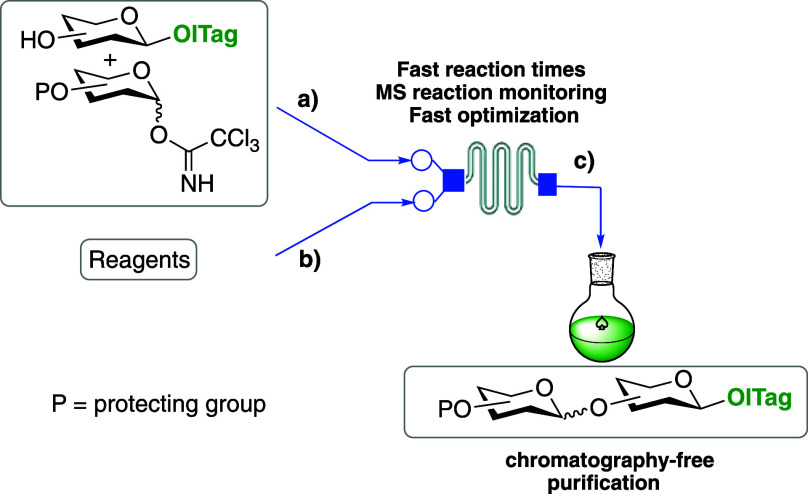
General ICROS-microflow strategy.

The microreactor glycosylation setup employed here is composed of a microfluidic borosilicate glass chip with a total internal volume of 18.7 μL, featuring two inlet ports containing the solution of the glycosyl donor and acceptor (a), catalyst (b), and a single outlet port (c) leading to a receiving flask (Figures 1 and S1). The inlet tubing pieces (a) and (b) are connected to syringe pumps, which control the reaction flow and thus reaction residence time. The small dimensions of the microreactor chip help maximize the rapid mixing of reagents and heat transfer that often lead to improved reaction outcomes.^24^

Initial efforts were aimed at evaluating the feasibility of incorporating the ICROS strategy into an in-flow glycosylation protocol (Table 1). To that end, a model glycosylation reaction between perbenzylated glucosyl trichloroacetimidate **1a** and HO-ITag1 **3a**,^25^ which features an imidazolium (ICROS) handle, was screened under different conditions, e.g., residence time (rt), reagent concentration, temperature, and solvent, and the outcome was monitored by MS and NMR (see Supporting Information for full details). The presence of the ITag label helped expedite the optimization process since the ITagged-species (i.e., starting material and product) could be easily monitored through MS. It was found that the reactions were completed much faster under microflow conditions compared to batch reactions, and product **4a** could be obtained in a rt of 15 s (Table 1, entry 2) at room temperature in 84% yield (1:1.4 α/β) when employing 2 equiv of **1a** and 1 equiv of **3a** in the presence of TMSOTf (0.45 equiv) in MeCN. We attribute the significant increase in reaction rate for microfluidic conditions, when compared to batch, to the microflow regime that facilitates a more homogeneous reaction mixing of the reagents and the imidazolium-tagged substrates during the glycosylation reaction due to a larger interfacial area.^26^ Product isolation was accomplished without the need of chromatography by simple trituration or/and biphasic washes in ether/hexanes mixtures of the dried crude mixture, as previously demonstrated for ITagged-glycosides.^9^ Interestingly, when comparing the reaction between **1a** and **3a** under batch conditions, reactions required 16 h to reach the same level of conversion, demonstrating that microflow conditions do indeed expedite the process (Table 1, entry 1). Next, the reaction conditions were evaluated in glycosylations involving other differentially protected glycosyl donors featuring silyl ethers, benzoyl, levulinyl, chloroacetyl, Fmoc, or acyl ester protecting groups **1b**-**e** with HO-ITag **3a**–**b**.^14^ In all cases, reaction optimization (catalyst loading: 0.25–1 equiv and room temperature) was expedited by the monitoring of the ITagged-species. The products were obtained in good yields of 72–91%, and most reactions reached completion within 15 s, with the exception of glycosylations with mannosyl donors, which required 1 min instead, likely due to the different reactivities of the donors (Table 1, entries 10 and 11). The reaction stereoselectivity outcome was not affected by the microfluidic conditions, with complete stereocontrol only observed for reactions carried out with donors bearing a OAc or OBz group at C-2, as in entries 3–5 and 8–10, as expected because of the neighboring group participation.^27^

**Table 1:**
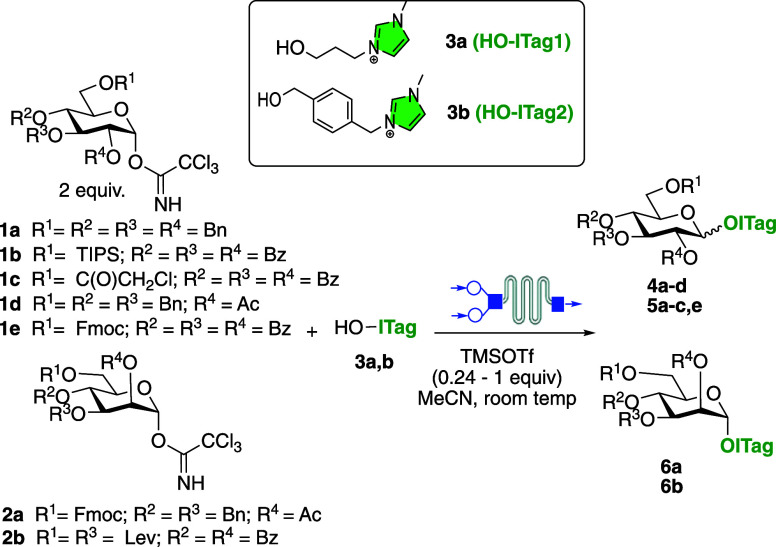
ICROS Microflow Glycosylation to Access ITagged Substrates **4–6**

entry	donor^a^	acceptor	rt^c^	product (%, α/β)
1^b^	**1a**	**3a**	16 h	**4a** (82, 1:1.4)
2	**1a**	**3a**	15 s	**4a** (84, 1:1.4)
3	**1b**	**3a**	15 s	**4b** (81, β)
4	**1c**	**3a**	15 s	**4c** (85, β)
5	**1d**^a^	**3a**	15 s	**4d** (72, β)
6	**1a**	**3b**	15 s	**5a** (90, 1:2.3)
7	**1b**	**3b**	15 s	**5b** (87, β)
8	**1c**	**3b**	15 s	**5c** (75, β)
9	**1e**	**3b**	15 s	**5e** (80, β)
10	**2a**	**3b**	60 s	**6a** (95, α)
11	**2b**	**3b**	60 s	**6b** (83, α)

a Reactions run with 2 equiv donor with the exception of **1d**, which required 3 equiv.

b Reaction in batch.

c Residence time.

To explore whether three reaction steps, including a functional group deprotection, could be telescoped using our system, C-6 silyl ether protected **1b** was glycosylated with HO-ITag1 **3a** under the optimized microflow conditions to give **4b** in just 15 s. The product was directly subjected to silyl ether deprotection in the collection flask using a mixture of 1.25 M HCl in MeOH, confirming through MS complete conversion into glycosyl acceptor **7** in 60 min (Scheme 1). Following ICROS purification as described before, compound **7** was subjected to microflow glycosylation conditions using glycosyl donor **1a** to give disaccharide **8** after 15 s in 80% overall yield and 1:2.5 α/β ratio.

**Scheme 1:**
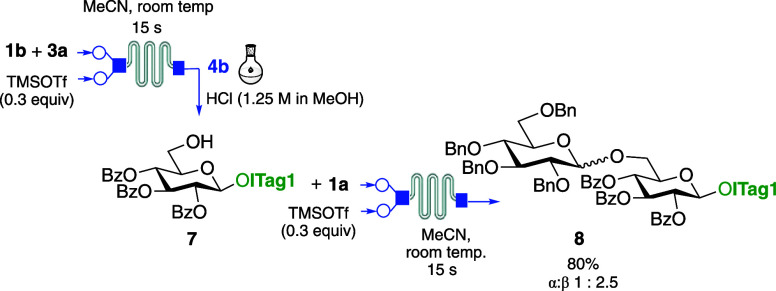
Microflow Synthesis of Disaccharide **8**

Having shown the feasibility of the approach, we wanted to demonstrate the versatility of the ICROS-microflow strategy in the preparation of α(1 → 6) trimannoside (Scheme 2), a critical component of the outer membrane of the *Mycobacterium tuberculosis* cell wall and key mediators of host–pathogen interactions.^28^ For this purpose, orthogonally protected trichloroacetimidate mannosyl donor **2a**, bearing an acetate group at C-2 to ensure α-selectivity, a Fmoc ester at position C-6, which can be orthogonally removed to allow glycoside extension, and benzyl ether protecting groups at C-3 and C-4 were chosen as the optimal starting building block. **3b** (HO-ITag2), which in addition to providing an MS reporter/purification handle, can be removed by catalytic hydrogenolysis at the end of the synthesis to release the product, was subjected to microflow glycosylation with **2a** to afford quantitatively **6a** in 1 min as determined by TLC-MS. Following purification of the dried reaction mixture via washes using an Et_2_O/H_2_O mixture, Fmoc deprotection was carried out in a vessel using a 10% solution of piperidine to give **9** after 20 min. Following purification via trituration using an Et_2_O/hexane mixture, acceptor **9** was submitted to two more cycles of the same microflow glycosylation conditions/batch deprotection/chromatography-free purification as before to provide trisaccharide **13**. Finally the ITag and OBn moieties were removed from **13** by catalytic hydrogenolysis, and the resulting trisaccharide was peracetylated and purified by flash silica gel column chromatography providing the protected α(1 → 6) trimannoside **14** in 11% overall yield after 8 steps. All reactions were monitored by MS to enable quick optimization of each reaction step. It is worth noting that the second and third glycosylation steps required longer reaction times in order to improve the conversion (2 and 4 min, respectively). Moreover, the target trimannoside **14** was prepared within one working day owing to the very fast reaction times and purification strategy. These results offer an improvement over previous syntheses of trimannoside derivatives using imidazolium-supported strategies, in which each glycosylation step required an overnight reaction.^12^

**Scheme 2:**
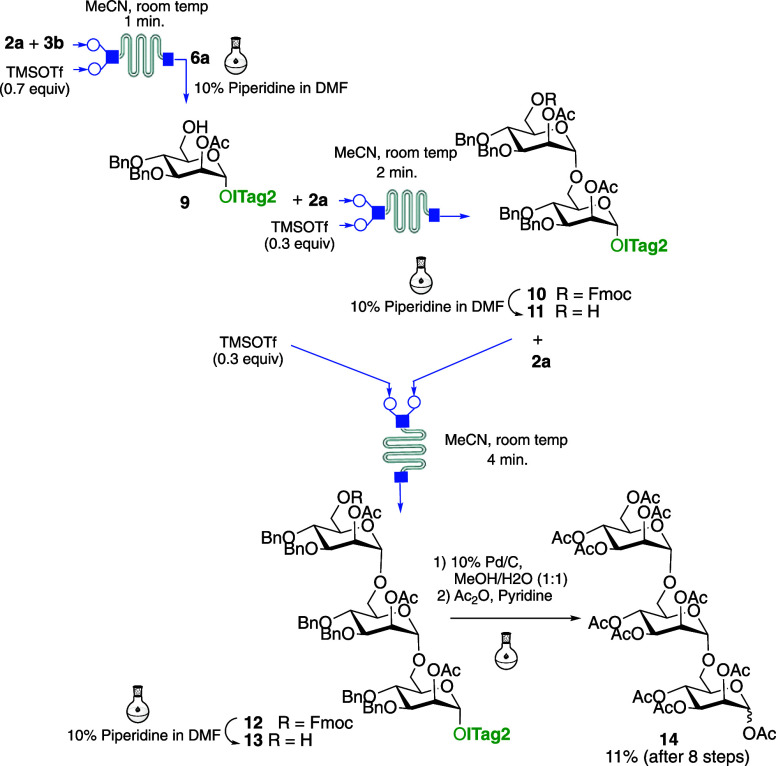
ICROS-Microflow Synthesis of Trimannoside **13**

Finally, we decided to further investigate the synthesis of a more complex and branched oligosaccharide, namely, Man_5_ oligomannoside, using the versatile and efficient ICROS-microflow strategy. This oligomannoside is a fragment of the relevant high-mannose oligosaccharide,^23^ the natural ligand of DC-SIGN receptor, which is involved in pathogen infection and immunomodulation processes.^29^ Toward this end, Man_5_ oligosaccharide **18** was prepared by a microflow glycosylation conditions/batch deprotection/chromatography-free purification strategy with a final silica gel purification step, as depicted in Scheme 3. Trichloroacetimidate mannosyl donor **2b** (see Supporting Information for synthetic details) was subjected to glycosylation with **3b** (2.5 equiv) under the optimized microflow conditions to give quantitively **6b** in 60 s as determined by MS. Following purification of the crude dried reaction via washing using an Et_2_O/H_2_O mixture, the levulinyl groups at C-3 and C-6 positions were orthogonally deprotected using a solution of hydrazine acetate and Py/AcOH in DCM in 2 h, as confirmed by MS to afford mannosyl acceptor **15**. Following ICROS-type purification as before, intermediate **15** was subjected to a [2 + 1] glycosylation with disaccharide donor **16**(30) to provide pentasaccharide **17** after 60 s with α-selectivity, as expected. It is worth noting that all glycosylation steps, including single and double glycosylation reactions, required shorter reaction times (60 s) with a total degree of conversion than the synthesis of previous trimannoside, where longer reaction times are required for each glycosylation step. Finally, cleavage of ITag **17** was tested using hydrogenolysis under H_2_ catalyzed by Pd/C (1 and 4 atm), PtO_2_, or Pt with various solvents, including acidic medium, which failed to remove the ionic tag, recovering the starting material. However, the ITag moiety was satisfactorily removed from dried crude containing compound **17** by transfer hydrogenolysis using resin-supported ammonium formate and Pd/C under microwave heating and purified by flash silica gel column chromatography, providing the **18** in 19% yield over 4 steps. In comparison with conventional synthesis (batch conditions) of this kind of complex of oligosaccharides, our methodology involves very fast reaction times and purification, and we were able to provide the target pentasaccharide **18** within one working day.

**Scheme 3:**
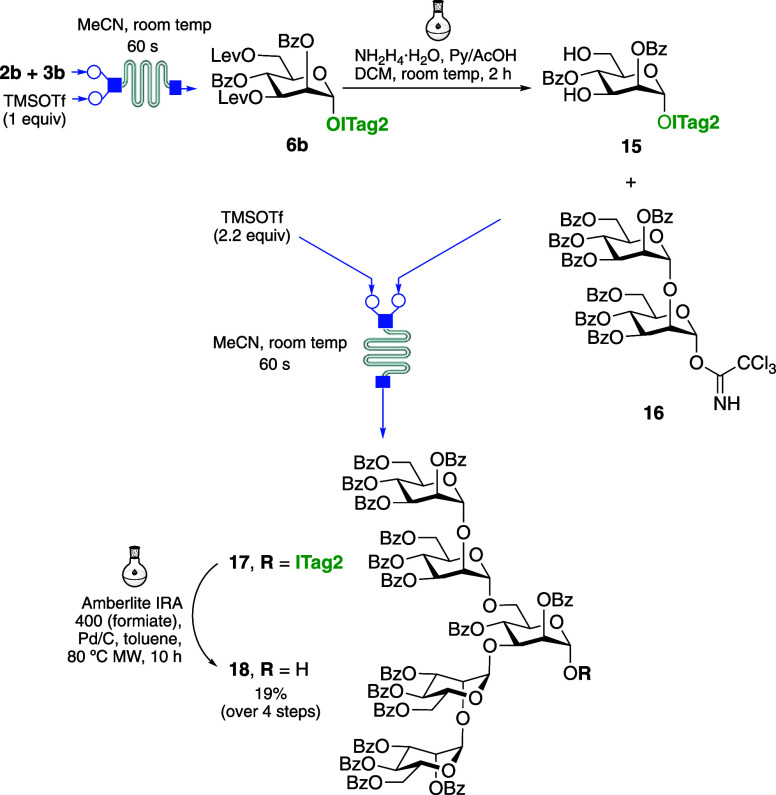
ICROS-Microflow Synthesis of Man_5_ Oligosaccharide **18**

## Conclusions

In conclusion, we have developed a continuous microfluidic glycosylation strategy that eliminates the need for chromatography between steps, significantly expediting the glycosylation chemistry. This approach leverages the benefits of imidazolium-based chromatography-free purification and in situ MS reaction monitoring, combined with continuous flow chemistry, to achieve shorter reaction times (ranging from 15 s to 4 min) and rapid reaction optimization. The reaction setup does not require expensive equipment and should be accessible to most laboratory environments. Our results demonstrate compatibility with the use of various orthogonal protecting groups and efficiency in the synthesis of a series of glycoside targets. Notably, we demonstrated that reactions can be telescoped and successfully synthesized an α-(1,6)-trimannoside and a branched Man_5_ oligomannoside, using an 8-step sequential glycosylation strategy or a 4-step [2 + 1] approach in 11% and 19%, respectively, within less than 24 h, demonstrating the flexibility and versatility of the system. While glycosylation yields are comparable per coupling step, our approach offers several advantages when comparing to literature reports of analogous structures prepared using traditional approaches in batch,^12,30,31^ such as longer reaction times, difficulty in monitoring reaction progress in situ, and need for silica gel chromatography after each step, making those strategies less expedient overall.

## Data Availability

The data supporting this article have been included as part of the Supporting Information. This includes synthetic protocols and characterization data for all compounds, including NMR spectra.

## Supporting Information Available

Synthetic protocols and microfluidics setup and characterization data for all compounds, including NMR spectra (PDF)
