# Correction to “Microfluidics-Based Ionic Catch and Release Oligosaccharide Synthesis (ICROS-Microflow) to Expedite Glycosylation Chemistry”

**DOI:** 10.1021/jacsau.5c01295

**Published:** 2025-10-08

**Authors:** Yao-Yao Zhang, Mattia Ghirardello, Ryan Williams, Adrian Silva Diaz, Javier Rojo, Josef Voglemeir, Javier Ramos-Soriano, M. Carmen Galan

We apologize that in the acknowledgments section of the original submission the EPSRC grant code EP/T020288/1 was listed incorrectly. Please find below the corrected list.

