# Supplementary material for: Microfluidics-Based Ionic Catch and Release Oligosaccharide Synthesis (ICROS-Microflow) to Expedite Glycosylation Chemistry
Source: JACS Au. 2024 Oct 21;4(11):4328–33. doi: 10.1021/jacsau.4c00686 (PMC11600195; doi:10.1021/jacsau.4c00686)

Supporting Information

Microfluidics-based Ionic Catch and Release Oligosaccharide Synthesis (ICROS-microflow) to expedite glycosylation chemistry.

Yao-Yao Zhang,[a,c]† Mattia Ghirardello,[a] Ryan Williams,[a] Adrian Silva Diaz,[b] Javier Rojo,[b] Josef Voglemeir,[c] Javier Ramos-Soriano,[b] M. Carmen Galan*[a]

[a]School of Chemistry, Cantock's Close, University of Bristol, BS8 1TS, UK; [b]Instituto de Investigaciones Químicas (IIQ), CSIC - Universidad de Sevilla, Av. Américo Vespucio 49, Seville 41092, Spain; [c] Glycomics and Glycan Bioengineering Research Center (GGBRC), College of Food Science and Technology, Nanjing Agricultural University, 1 Weigang, 210095 Nanjing (China)

*E-mail: m.c.galan@bristol.ac.uk

Experimental Procedures

General. Chemicals were purchased and used without further purification except for benzyl galactal which was passed through a plug of silica before use. Dry solvents were obtained by distillation using standard procedures or by passage through a column of anhydrous alumina using equipment from Anhydrous Engineering (University of Bristol) based on the Grubbs' design. Reactions requiring anhydrous conditions were performed under nitrogen; glassware and needles were either flame dried immediately prior to use or placed in an oven (150 °C) for at least 2 hours and allowed to cool either in a desiccator or under reduced pressure; liquid reagents, solutions or solvents were added *via* syringe through rubber septa; solid reagents were added *via* Schlenk type adapters. Teflon rings were used between the joints of the condensers and round bottom flasks. Reactions were monitored by TLC on Kieselgel 60 F254 (Merck). Detection was by examination under UV light (254 nm) and by charring with 10% sulfuric acid in ethanol. Flash column chromatography was performed using silica gel [Merck, 230−400 mesh (40−63 μm)]. Extracts were concentrated *in vacuo* using both a Büchi rotary evaporator (bath temperatures up to 40 °C) at a pressure of either 15 mmHg (diaphragm pump) or 0.1 mmHg (oil pump), as appropriate, and a high vacuum line at room temperature. Mass spectra were obtained by the University of Bristol Mass Spectrometry Service using an ESI (Bruker Daltonics micrOTOF II), Nanospray (Waters Synapt G2S), or MALDI (Bruker ultrafleXtreme 2) spectrometer. *X*-ray crystallography was performed by the University of Bristol Crystallography Service using a Bruker Kappa Apex II diffractometer. Optical rotations were recorded on an ADP220 polarimeter (Bellingham and Stanley) using the sodium D line (λ = 589 nm). MS detection for routine in situ reaction monitoring was carried out on Advion's expression® Compact Mass Spectrometer (CMS) creating a technique known as TLC/CMS. ^{1}H NMR and ^{13}C NMR spectra were measured in the solvent stated at 400 or 500 MHz. Chemical shifts are quoted in parts per million from residual solvent peak ($CDCl_3$: ^{1}H - 7.26 ppm and ^{13}C - 77.16 ppm) and coupling constants (*J*) given in Hertz. Multiplicities are abbreviated as: b (broad), s (singlet), d (doublet), t (triplet), q (quartet), m (multiplet) or combinations thereof. The units of the

specific rotation, (deg·mL)/(g·dm), are implicit and are not included with the reported value. Concentration *c* is given in g/100 mL.

General microfluidics reaction set up. The flow reactor was purchased from Micronit Microfludics. The microreactor setup consist of a microfluidic borosilicate glass slide with a channel etched into it, for a total internal volume of 18.7 µL (Figure S1). The microreactor chip features two inlet ports and a single outlet port, where PTFE tubing can be affixed using ferrules. The PTFE tubing used in this system has a much a smaller internal diameter (0.03 cm) than for the coil reactor tubing, in line with the smaller dimensions of the channels in the reactor chip. The inlet tubing pieces are connected to syringes, whilst the outlet tubing piece leads to a receiving flask. The small dimensions of this microreactor chip maximise the rapid mixing and heat transfer that make flow chemistry advantageous, whilst proving well suited to very short residence times, or to small quantities of material. NE-300 Syringe infusion pumps were used. 2-5 mL standard plastic syringes were used for these experiments depending on scale.

Residence time (rt) is defined as the time the reagents are in the microfluidic channel (and outlet tubing) divided by the in flow rate. For example, since the internal volume of the chamber and outlet tubing is 32.8 µL, a reaction flow rate of 131.20 µL/min, given by the combined flow of 65.6 µL/min per syringe, will lead to a 0.25 min (= 15 s) residence time.

Microfluidic reaction optimization. Reactions were run as described above and the reactants collected in the receiving flask. A small aliquot of the mixture was then taken for MS analysis. For TLS-MS analysis: 1 µL of sample was deposited on a TLC plate and analysed directly using an Advion Plate Express module attached to an Advion Expression ion CMS machine. Samples were also analysed using MALDI-TOF or ESI to obtain high resolution MS characterization data. See Figure S1 for an example of a TLC-MS spectra of glycosylation reaction (**2a** + **11**) showing incomplete conversion. Incomplete reactions, will be resubjected to reaction conditions and changes to flow rate (residence time), concentration of reagents (e.g. equiv of donor and/or lewis acid), reaction solvent, reaction temperature will be modified systematically for optimum results.

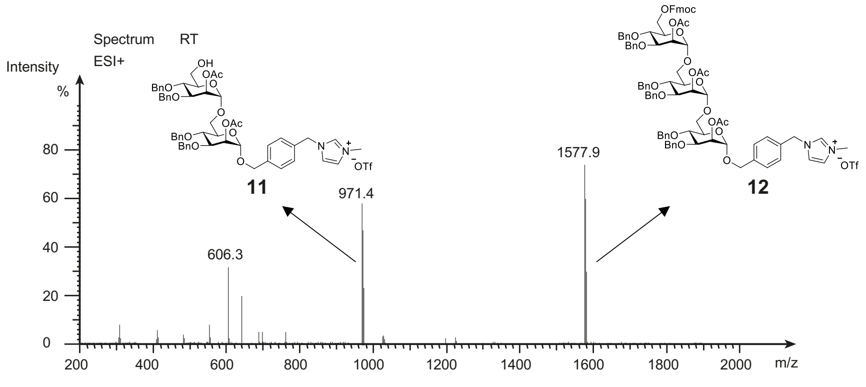

Figure S1. TLC-MS spectra of reaction between **2a** and **11** to yield **12.** Incomplete reaction shown as monitored by MS *in situ*.

A)

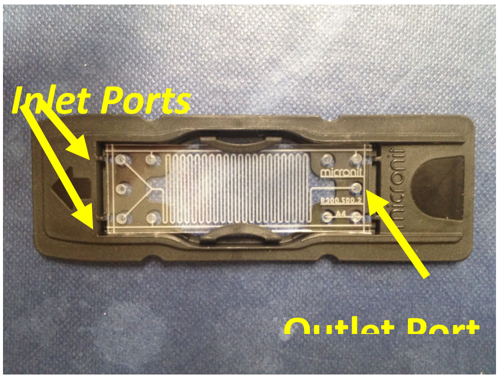

B)

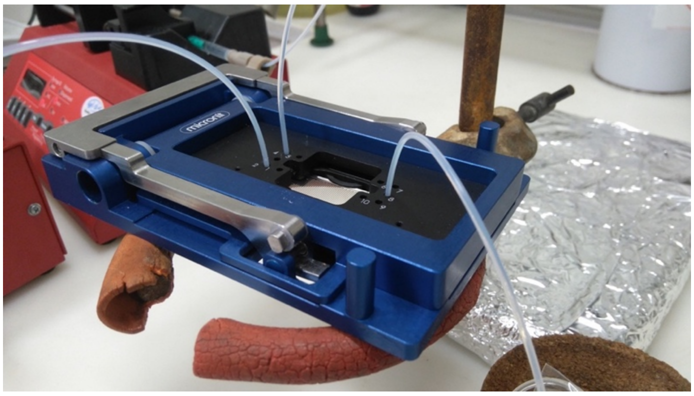

C)

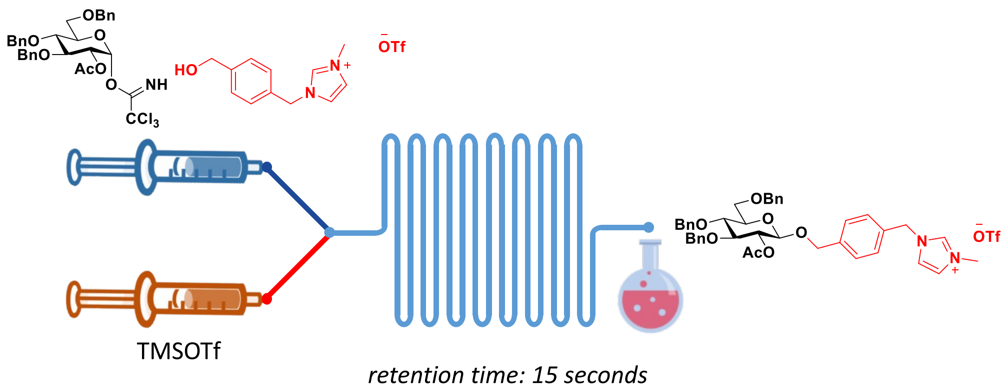

Figure S2. Microreactor chip used for flow reactions. A) Microchip used in the study with the inlet and outlet ports highlighted. B) Picture of the set up with microchip fixed in its cradle, and tubing attached to pumps. C) Schematic of typical reaction set up with donor and acceptor injected into one of the channels and the Lewis acid in the other prior to microflow mixing.

Experimental details

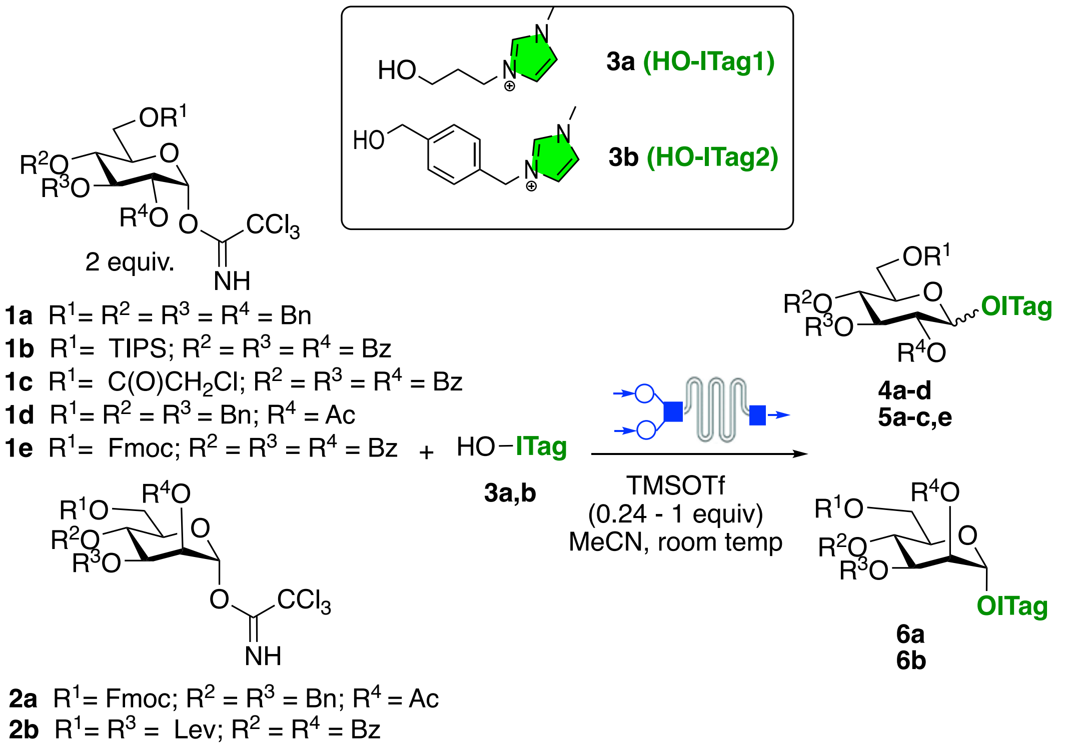

Scheme S1

1-(3-Hydroxypropyl)-3-methyl-1H-imidazol-3-ium trifluoromethanesulfonate (3a)

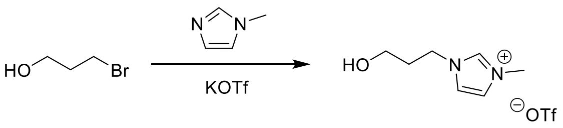

3-Bromopropanol (5 mL, 55.40 mmol), 1-methylimidazole (13.3 mL, 166.20 mmol), potassium trifluoromethanesulfonate (20.9 g, 110.80 mmol) and anhydrous acetonitrile (100 mL) were added to an oven-dried flask. The resulting mixture was heated under reflux at 80 ℃ under a N_2 atmosphere for 21 h. ^{1}H NMR analysis of the crude material revealed that the alcohol has been converted to the product quantitatively. The reaction mixture was filtered and the solvent was removed under reduced pressure. The crude product was heated to 80 ℃ under vacuum (0.6 mbar) overnight and subsequently triturated five times (Et_2O:DCM, 13:7). To remove final traces of 1-methylimidazole, a 0.50 g portion of the product was purified by reverse phase HPLC (H_2O:MeCN) and freeze-dried, to afford the title compound **3a** as a colourless oil (0.40 g, 80 %). ^{1}H NMR (400 MHz, $CDCl_3$) δ 8.48 (1 H, s, NC*H*N), 7.41

(1 H, t, J = 1.8 Hz, NCHCHN), 7.35 (1 H, t, J = 1.8 Hz, NCHCHN), 4.26 (2 H, t, J = 7.0 Hz, CH_2), 3.84 (3 H, s, CH_3), 3.54 (2 H, t, J = 5.9 Hz, CH_2), 2.41 (1 H, bs, OH), 2.05 – 1.98 (2 H, m, CH_2); ^{13}C NMR (101 MHz, $CDCl_3$) δ 137.2 (NCHN), 124.6, 123.5 (2 x NCHCHN), 58.5, 47.7, 36.8, 33.1; m/z (ESI-MS+) $C_7H_{13}N_2O_7^+$ [M - OTf]$^+$ calcd: 141.1; found 141.1. Data in agreement with reported literature.[1]

3-(3-Methylimidazolium)-1-propyl 2,3,4,6-tetra-O-benzyl-D-glucopyranoside trifluoromethanesulfonate (4a)

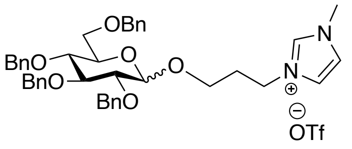

A solution containing ITag acceptor **3a** (58 mg, 0.2 mmol) and glycosyl donor **1a** (280 mg, 0.4 mmol) in 1.0 mL of anhydrous MeCN was loaded on a syringe. A second syringe was loaded with trimethylsilyl trifluoromethanesulfonate (16.3 µL, 0.090 mmol) in 1.5 mL of anhydrous MeCN. An overall flow rate of 131.2 µL/min was set (each pump was set to a rate of 65.6 µL/min). The reaction solution was collected in a flask containing triethylamine in DCM to quench the reaction. The crude product was washed with water (3 mL), then the water was extracted with DCM (2 x 15 mL). The dried residue was washed with Hexane:Et_2O 1:1 (3 x 5 mL) then dried under reduced pressure to yield **4a** as an oil (136 mg, 84 %, α:β 1 : 1.4). ^{1}H NMR (500 MHz, MeCN-d_3) δ 8.45 (1 H, s, NCHN α), 8.40 (1 H, s, NCHN β), 7.41 – 7.18 (m, H_{Ar}), 4.93 – 4.87 (m, H-1 α), 4.87 – 4.65 (m, PhCH_2), 4.59 – 4.47 (m, PhCH_2), 4.42 (1 H, d, J = 7.8 Hz, H-1 β), 4.31 – 4.16 (m, CH_2), 3.84 – 3.60 (m), 3.71 (3 H, s, NCH_3 β), 3.63 (3 H, s, NCH_3 α), 3.55 – 3.48 (m), 3.43 (1 H, dt, J = 10.3, 6.1 Hz, C-1OCH_2 α), 3.29 (1 H, t, J = 8.5 Hz , H-2 β), 2.11 (4 H, dt, J = 9.7, 6.0 Hz, $CH_2C\underline{H}_2CH_2$); ^{13}C NMR (126 MHz, MeCN-d_3) δ 140.1, 139.9, 139.8, 139.6, 139.5, 139.5, 139.4, 139.3, 137.3 (NCHN β), 137.2 (NCHN α), 129.4, 129.4, 129.3, 129.3, 129.2, 129.0, 129.0, 128.9, 128.9, 128.8, 128.8, 128.7, 128.6, 128.6, 128.5, 128.4, 124.4, 124.3, 123.6, 123.5, 104.0 (C-1 β), 97.6 (C-1 α), 85.3, 83.0 (C-2 β), 82.6, 81.1, 78.9, 78.9, 76.0, 75.9, 75.7, 75.5, 75.2, 75.2, 73.8, 73.8, 73.6, 71.5, 70.0, 69.9, 66.6, 65.3 (C-1OCH$_2$), 48.1, 47.8, 36.7 (NCH_3), 36.6 (NCH_3), 30.7 ($CH_2\mathit{C}H_2CH_2$), 30.2 ($CH_2\mathit{C}H_2CH_2$); m/z (TLC-MS$^+$) $C_{41}H_{47}N_2O_6^+$ [M - OTf]$^+$ calcd: 663.3; found 663.9. Data in agreement with literature.[2]

***3-(3-Methylimidazolium)-1-propyl 2,3,4-tri-O-benzoyl-6-O-triisopropylsilyl-β-D-glucopyranoside trifluoromethanesulfonate* (4b)**

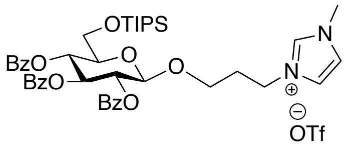

A solution containing ITag acceptor **3a** (58 mg, 0.2 mmol) and glycosyl donor **1b** (317 mg, 0.4 mmol) in 1.0 mL of anhydrous MeCN was loaded on a syringe. A second syringe was loaded with trimethylsilyl trifluoromethanesulfonate (8.7 μL, 0.048 mmol) in 1.5 mL of anhydrous MeCN. An overall flow rate of 131.2 μL/min was set (each pump was set to a rate of 65.6 μL/min). The reaction solution was collected in a flask containing triethylamine in DCM to quench the reaction. The crude product was washed with water (3 mL), then the water was extracted with DCM (2 x 15 mL). The dried residue was washed with Hexane:Et_2O 1:1 (3 x 5 mL) then dried under reduced pressure to yield **4b** as an oil (149 mg, 81 %, β). ^1H NMR (400 MHz, $CDCl_3$) δ 9.13 (s, 1H, NCHN), 7.97-7.16 (m, 17H, 15H (Ph) + 2H (imidazolium), 5.86 (t, 1H, $J_{3,4}$ = $J_{3,2}$ = 9.5 Hz, H-3), 5.60 (t, 1H, $J_{4,3}$ = $J_{4,5}$ = 9.5 Hz, H-4), 5.38 (dd, 1H, $J_{2,1}$ = 8.0 Hz, $J_{2,3}$ = 9.5 Hz, H-2), 4.80 (d, 1H, $J_{1,2}$ = 8.0 Hz, H-1), 4.31-4.25 (m, 2H, CH_2N), 3.97-3.87 (m, 7H in which s at 3.93, CH_3N, H-5, H-6a, H-6b, OCH_2), 3.42-3.31 (m, 1H, OCH_2), 2.19-2.15 (m, 2H, $OCH_2C\underline{H}_2$), 1.08-0.95 (m, 21H, TIPS); ^{13}C NMR (100 MHz, $CDCl_3$) δ 165.8, 165.4, 165.1, 137.0 (N*C*HN), 133.7, 133.3, 129.7, 129.1, 129.0, 128.9, 128.8, 128.7, 128.6, 128.4, 128.3, 128.2, 125.3, 122.9, 122.8, 100.9 (C-1), 75.5 (C-5), 73.0 (C-3), 72.2 (C-2), 69.1 (C-4), 65.7 (OCH_2), 62.5 (C-6), 47.2 (CH_2N), 36.4 (CH_3N), 29.9 ($OCH_2\mathit{C}H_2$), 17.8, 11.9 (TIPS); m/z (ESI-HRMS) for $C_{42}H_{52}N_2O_9Si^+$ $[M - OTf]^+$, calcd: 756.3437, found: 756.3431. Data in agreement with reported literature.[3]

***3-(3-Methylimidazolium)-1-propyl 2,3,4-tri-O-benzoyl-6-O-chloroacetyl-β-D-glucopyranoside trifluoromethanesulfonate* (4c)**

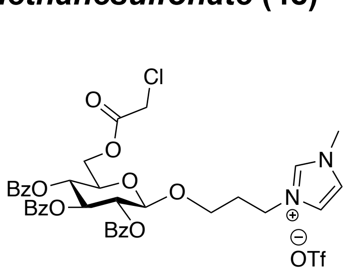

A solution containing ITag acceptor **3a** (116 mg, 0.4 mmol) and glycosyl donor **1c** (571 mg, 0.8 mmol) in 2.0 mL of anhydrous MeCN was loaded on a syringe. A

second syringe was loaded with trimethylsilyl trifluoromethanesulfonate (32.6 μL, 0.18 mmol) in 3 mL of anhydrous MeCN. An overall flow rate of 131.2 μL/min was set (each pump was set to a rate of 65.6 μL/min). The reaction solution was collected in a flask containing triethylamine in DCM to quench the reaction. The crude product was washed with water (5 mL), then the water was extracted with DCM (2 x 20 mL). The dried residue was washed with Hexane:Et_2O 1:1 (3 x 10 mL) then dried under reduced pressure to yield **4c** as an oil (282 mg, 85 %, β). ^{1}HNMR (400 MHz, MeCN-*d*3) *δ* 8.31 (1 H, s, NCHN), 7.96 – 7.88 (4 H, m, H_{Ar}), 7.78 – 7.74 (2 H, m, H_{Ar}), 7.60 (2 H, m, H_{Ar}), 7.52 (1 H, ddt, *J* = 7.6, 6.7, 1.3 Hz, H_{Ar}), 7.45 (4 H, td, *J* = 7.7, 3.4 Hz, H_{Ar}), 7.39 – 7.33 (2 H, m, H_{Ar}), 7.28 (1 H, t, *J* = 1.7 Hz, NC*H*CHN), 7.26 (1 H, t, *J* = 1.7 Hz, (NCHC*H*N), 5.89 (1 H, t, *J* = 9.6 Hz, H-3), 5.57 (1 H, t, *J* = 9.7 Hz, H-4), 5.39 (1 H, dd, *J* = 9.7, 8.0 Hz, H-2), 4.97 (1 H, d, *J* = 8.0 Hz, H-1), 4.46 (1 H, dd, *J* = 12.3, 4.9 Hz, H-6a), 4.40 (1 H, dd, *J* = 12.4, 2.6 Hz, H-6b), 4.21 (2 H, s, CH_2Cl), 4.23 – 4.18 (1 H, m, H-5), 4.12 (2 H, td, *J* = 6.8, 3.5 Hz, $OCH_2CH_2C\mathit{H}_2N$), 3.87 (1 H, ddd, *J* = 11.0, 7.1, 4.8 Hz, $OC\mathit{H}HCH_2CH_2N$), 3.81 (3 H, s, NCH_3), 3.68 (1 H, ddd, *J* = 10.7, 6.2, 5.0 Hz, $OCH\mathit{H}CH_2CH_2N$), 2.11 – 1.99 (2 H, m, $OCH_2C\mathit{H}_2CH_2N$); ^{13}C NMR (101 MHz, MeCN-*d*3) *δ* 168.1, 166.3, 166.1, 166.0, 137.0 (NCHN), 134.6, 134.5, 130.3, 130.1, 129.9, 129.8, 129.7, 129.5 (C_{Ar}), 124.4 (N*C*HCHN), 123.3 (NCH*C*HN), 101.2 (C-1), 74.2 (C-3), 72.8 (C-2), 72.5 (C-5), 70.0 (C-4), 66.9 ($O\mathit{C}H_2CH_2CH_2N$), 64.2 (C-6), 47.5 ($OCH_2CH_2\mathit{C}H_2N$), 41.9 (CH_2Cl), 36.8 (NCH_3), 30.4 ($OCH_2\mathit{C}H_2CH_2N$); m/z (ESI-HRMS) for $C_{36}H_{36}ClN_2O_{10}^+$ [M - OTf]$^+$ calcd: 691.2053; found: 691.2077.

3-(3-Methylimidazolium)-1-propyl 2-O-acetyl-3,4,6-tri-O-benzyl-β-D-glucopyranoside trifluoromethanesulfonate (4d)

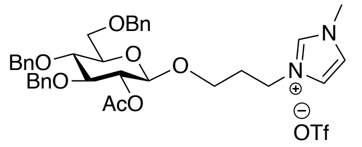

A solution containing ITag acceptor **3a** (12 mg, 0.040 mmol) and glycosyl donor **1d** (86 mg, 0.120 mmol) in 0.2 mL of anhydrous MeCN was loaded on a syringe. A second syringe was loaded with trimethylsilyl trifluoromethanesulfonate (2.27 μL, 0.012 mmol) in 0.4 mL of anhydrous MeCN. An overall flow rate of 131.2 μL/min was set (each pump was set to a rate of 65.6 μL/min). The reaction solution was collected in a flask containing triethylamine in DCM to quench the reaction. The crude product was washed with water (2 x 2 mL), then the water was extracted with DCM (3 x 5 mL). The

dried residue was washed with hexane (3 x 2 mL) and hexane:Et_2O 1:1 (3 x 2 mL) then dried under reduced pressure to yield **4d** as an oil (22 mg, 72 %, β); ^{1}H NMR (500 MHz, $CDCl_3$) δ 9.04 (1 H, s, NC*H*N), 7.38 – 7.22 (14 H, m, H_{Ar}), 7.20 (2 H, dd, *J* = 7.4, 1.9 Hz, H_{Ar}), 6.89 (1 H, s, NC*H*CHN), 4.84 – 4.73 (3 H, m, 2 $PhCH_2$, H-2), 4.66 (1 H, d, *J* = 11.3 Hz, PhC*H*H), 4.57 (2 H, app d, *J* = 10.9 Hz, $PhCH_2$), 4.50 (1 H, d, *J* = 11.6 Hz, PhCH*H*), 4.33 (1 H, d, *J* = 8.1 Hz, H-1), 4.31 – 4.26 (2 H, m, $OCH_2CH_2CH_2N$), 3.80 (3 H, s, NCH_3), 3.78 – 3.59 (6 H, m, H-3, H-4, H-6a, H-6b, $OCH_2CH_2CH_2N$), 3.53 – 3.45 (1 H, m, H-5), 2.13 (2 H, d, *J* = 7.4 Hz, $OCH_2CH_2CH_2N$), 1.95 (3 H, s, $COCH_3$); ^{13}C NMR (126 MHz, $CDCl_3$) δ 169.98, 138.1, 137.9, 137.8, 137.5 (N*C*HN), 128.7, 128.6, 128.6, 128.1, 128.1, 128.0, 127.9, 123.3 (NCH*C*HN), 122.8 (N*C*HCHN), 100.9 (C-1), 82.8 , 77.9 (C-3, C-4), 75.5 ($PhCH_2$), 75.2 ($PhCH_2$), 74.8 (C-5), 73.6 ($PhCH_2$), 73.2 (C-2), 68.8 (C-6), 66.1 ($OCH_2CH_2CH_2N$), 47.7 ($OCH_2CH_2CH_2N$), 36.5 (NCH_3), 30.1 ($OCH_2CH_2CH_2N$), 21.1 ($COCH_3$); *m/z* (ESI-HRMS) for $C_{36}H_{43}N_2O_7^+$ [M - OTf]$^+$ calcd: 615.3065; found 615.3070.

4-(1-Methyl-3-methyleneimidazolium)benzyl 2,3,4,6-tetra-O-benzyl-D-glucopyranoside trifluoromethanesulfonate (5a)

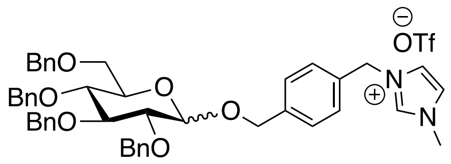

A solution containing ITag acceptor **3b**[2] (71 mg, 0.2 mmol) and glycosyl donor **1d** (411 mg, 0.6 mmol) in 2.0 mL of anhydrous MeCN was loaded on a syringe. A second syringe was loaded with trimethylsilyl trifluoromethanesulfonate (32.5 µL, 0.18 mmol) in 1.5 mL of anhydrous MeCN. An overall flow rate of 131.2 µL/min was set (each pump was set to a rate of 65.6 µL/min). The reaction solution was collected in a flask containing triethylamine in DCM to quench the reaction. The crude product was washed with water (2 x 2.5 mL), then the water was extracted with DCM (3 x 5 mL). The dried residue was washed with hexane (3 x 5 mL) and hexane:Et_2O 1:1 (6 x 5 mL) then dried under reduced pressure to yield **5a** as an oil (158 mg, 90 %, α:β 1 : 2). ^{1}H NMR (500 MHz, MeCN-d_3) δ 8.56 (2 H, bs, NC*H*N α and β), 7.48 – 7.20 (m, H_{Ar}), 5.03 (1 H, d, *J* = 3.5 Hz, H-1 α), 4.92 – 4.46 (m), 3.81 (3 H, s, NCH_3 β), 3.80 (3 H, s, NCH_3 α), 3.86 – 3.46 (m), 3.40 (1 H, dd, *J* = 8.5, 7.9 Hz, H-2 β); ^{13}C NMR (126 MHz, MeCN-d_3) δ 140.0, 139.9, 139.8, 139.7, 139.6, 139.5, 139.5, 139.4, 137.1 (N*C*HN α and β), 134.2, 134.1, 129.7, 129.6, 129.5, 129.5, 129.3, 129.3, 129.2, 129.2, 129.1, 128.9, 128.8, 128.8, 128.8, 128.8, 128.7, 128.7, 128.7, 128.6, 128.5, 128.5, 128.5, 128.4,

128.4, 128.3, 128.2, 125.0 (N*C*HCHN), 124.9 (N*C*HCHN), 123.1 (N*C*HCHN), 103.3 (C-1 β), 97.0 (C-1 α), 85.3, 83.1 (C-2 β), 82.6, 81.1, 78.8, 78.8, 76.0, 75.9, 75.5, 75.4, 75.4, 75.2, 73.8, 73.8, 73.2, 71.5, 71.0, 69.9, 69.8, 69.5, 36.9 (N*C*H_3), 36.9 (N*C*H_3); m/z (ESI-HRMS) $C_{46}H_{49}N_2O_6^+$ [M - OTf]$^+$ calcd: 725.3585; found 725.3583.

***4-(1-Methyl-3-methyleneimidazolium)-benzyl 2,3,4-tri-O-benzoyl-6-O-triisopropylsilyl-β-D-glucopyranoside trifluoromethanesulfonate* (5b)**

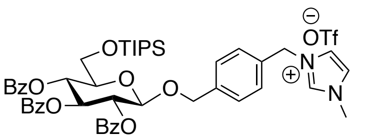

A solution containing ITag acceptor **3b** (71 mg, 0.2 mmol) and glycosyl donor **1b** (476 mg, 0.6 mmol) in 2.0 mL of anhydrous MeCN was loaded on a syringe. A second syringe was loaded with trimethylsilyl trifluoromethanesulfonate (32.5 μL, 0.18 mmol) in 1.5 mL of anhydrous MeCN. An overall flow rate of 131.2 μL/min (each pump was set to a rate of 65.6 μL/min) was set. The reaction solution was collected in a flask containing triethylamine in DCM to quench the reaction. The crude product was washed with water (2 x 2.5 mL), then the water was extracted with DCM (3 x 5 mL). The dried residue was washed with hexane (3 x 5 mL) and hexane:Et_2O 1:1 (6 x 5 mL) then dried under reduced pressure to yield **5b** as an oil (171 mg, 87 %, β). ^{1}H NMR (400 MHz, $CDCl_3$) *δ* 9.20 (s, 1H, NC*H*N), 7.94-7.91 (m, 4H, H_{Ar}), 7.84-7.82 (m, 2H, H_{Ar}), 7.57-7.51 (m, 2H, H_{Ar}), 7.45-7.36 (m, 5H, H_{Ar}), 7.31-7.23 (m, 7H, H_{Ar}), 7.17 (t, 1H, *J* = 1.5 Hz, NC*H*CHN), 5.86 (t, 1H, $J_{3,4}$ = $J_{3,2}$ = 9.5 Hz, H-3), 5.59 (t, 1H, $J_{4,3}$ = $J_{4,5}$ = 9.5 Hz, H-4), 5.55 (dd, 1H, $J_{2,1}$ = 8.0 Hz, $J_{2,3}$ = 9.5 Hz, H-2), 5.31 (bs, 2H, CH_2N), 4.95 (d, 1H, *J* = 12.0 Hz, OCH_2), 4.87 (d, 1H, $J_{1,2}$ = 8.0 Hz, H-1), 4.70 (d, 1H, *J* = 12.0 Hz, OCH_2), 3.99-3.96 (m, 2H, H-6a, H-6b), 3.93 (s, 3H, NCH_3), 3.93-3.88 (m, 1H, H-5), 1.09-1.03 (m, 21H, TIPS); ^{13}C NMR (100 MHz, $CDCl_3$) *δ* 165.8, 165.2, 165.1, 138.6, 137.0, 133.4, 133.3, 133.2, 132.1, 129.7, 129.6, 129.2, 129.1, 129.0, 128.9, 128.7, 128.4, 128.3, 128.2, 123.5, 121.8(NCHN), 99.7 (C-1), 75.6 (C-5), 73.2 (C-3), 72.0 (C-2), 69.7 (OCH_2), 69.4 (C-4), 62.7 (C-6), 53.1 (CH_2N), 36.4 (NCH_3), 17.8, 11.9 (TIPS); *m/z* ESI-HRMS [M$^+$] for $C_{48}H_{57}N_2O_9Si^+$ calcd: 833.3828; found: 833.3828. Data in agreement with reported literature.[3]

4-(1-Methyl-3-methyleneimidazolium)benzyl 2,3,4-tri-O-benzoyl-6-O-chloroacetyl-β-D-glucopyranoside trifluoromethanesulfonate (5c)

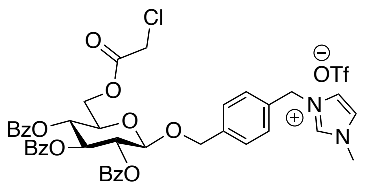

A solution containing ITag acceptor **3b** (56 mg, 0.16 mmol) and glycosyl donor **1b** (228 mg, 0.320 mmol) in 0.8 mL of anhydrous MeCN was loaded on a syringe. A second syringe was loaded with trimethylsilyl trifluoromethanesulfonate (17 µL, 0.09 mmol) in 1.5 mL of anhydrous MeCN. A flow rate of 131.2 µL/min was set. The reaction solution was collected in a flask containing triethylamine in DCM to quench the reaction. The crude product was dissolved in DCM (5 mL) and washed with water (5 mL), then the water was extracted with further portions of DCM (2 x 5 mL). The dried residue was washed with Et_2O (2 x 6 mL), then washed with DCM:Et_2O 5:95 (3 x 6 mL) and DCM:Et_2O 1:9 (4 x 6 mL) then dried under reduced pressure to yield the title compound **5c** as a syrup (108 mg, 75 %, β). ^{1}H NMR (500 MHz, $CDCl_3$) δ 9.16 (1 H, s, NCHN), 7.93 – 7.87 (4 H, m, H_{Ar}), 7.81 – 7.78 (2 H, m, H_{Ar}), 7.56 – 7.48 (2 H, m, H_{Ar}), 7.44 – 7.33 (5 H, m, H_{Ar}), 7.29 – 7.21 (7 H, m, H_{Ar}, NC*H*CHN), 7.17 (1 H, t, *J* = 1.8 Hz, NCHC*H*N), 5.86 (1 H, t, *J* = 9.7 Hz, H-3), 5.58 (1 H, t, *J* = 9.7 Hz, H-4), 5.55 (1 H, dd, *J* = 9.8, 7.9 Hz, H-2), 5.28 (2 H, s, NCH_2), 4.92 – 4.88 (2 H, m, H-1, (C-1)OC*H*H), 4.69 (1 H, d, *J* = 12.6 Hz, (C-1)OCH*H*), 4.43 – 4.40 (2 H, m, H-6a, H-6b), 4.10 (2 H, s, CH^2Cl), 4.09 – 4.03 (1 H, m, H-5), 3.90 (3 H, s, NCH_3); ^{13}C NMR (100 MHz, $CDCl_3$) δ 167.2 (CO (ClAc)), 165.8, 165.4, 165.3 (3 C=O (Bz)), 138.6, 137.1 (NCHN), 133.8, 133.7, 133.5, 132.3, 129.9, 129.9, 129.8, 129.2, 129.2, 128.8, 128.8, 128.7, 128.7, 128.6, 128.5, 123.7 (N*C*HCHN), 122.0 (NCH*C*HN), 100.2 (C-1), 72.8 (C-3), 72.1 (C-5), 71.9 (C-2), 70.5 ((C-1)O*C*H_2), 69.3 (C-4), 63.9 (C-6), 53.3 (NCH_2), 40.9 (CH_2Cl), 36.6 (NCH_3); *m/z* (ESI-HRMS) for $C_{41}H_{38}ClN_2O_{10}^+$ [M - OTf]$^+$ calcd: 753.2209; found: 7533.2208.

4-(1-Methyl-3-methyleneimidazolium)benzyl 2,3,4-tri-O-benzoyl-6-O-(9-fluorenylmethoxycarbonyl)-β-D-glucopyranoside trifluoromethanesulfonate (5e)

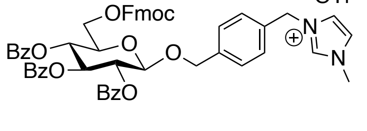

A solution containing ITag acceptor **3b** (29 mg, 0.08 mmol) and glycosyl donor **1b** (140 mg, 0.16 mmol) in 0.41 mL of anhydrous MeCN was loaded on a syringe. A

second syringe was loaded with trimethylsilyl trifluoromethanesulfonate (8.9 µL, 0.05 mmol) in 1.5 mL of anhydrous MeCN. An overall flow rate of 131.2 µL/min was set (each pump was set to a rate of 65.6 µL/min). The reaction solution was collected in a flask containing triethylamine in DCM to quench the reaction. The crude product was dissolved in DCM (5 mL) and washed with water (5 mL), then the water was extracted with further portions of DCM (2 x 5 mL). The dried residue was washed with hexane (5 mL), hexane:Et_2O 1:1 (2 x 5 mL), hexane:Et_2O 1:3 (2 x 5 mL), and Et_2O (3 x 5 mL), then dried under reduced pressure to yield the title compound **5e** as a syrup (69 mg, 80 %, β); ^{1}H NMR (500 MHz, $CDCl_3$) *δ* 9.19 (1 H, s, NH), 7.95 – 7.90 (4 H, m, H_{Ar}), 7.82 (2 H, app dd, *J* = 8.4, 1.4 Hz, H_{Ar}), 7.75 (2 H, app d, *J* = 7.5 Hz, H_{Ar}), 7.64 – 7.46 (4 H, m, H_{Ar}), 7.47 – 7.16 (15 H, m, H_{Ar}), 7.16 (1 H, s, NC*H*CHN), 7.07 (1 H, s, NCHC*H*N), 5.88 (1 H, t, *J* = 9.6 Hz, H-3), 5.62 – 5.53 (2 H, m, H-2, H-4), 5.25 (2 H, s, NCH_2), 4.94 – 4.86 (2 H, m, H-1, (C-1)OC*H*H), 4.67 (1 H, d, *J* = 12.5 Hz, (C-1)OCH*H*), 4.47 – 4.33 (4 H, m, H-6a, H-6b, OC*H*$_2$CH(Fmoc)), 4.22 (1 H, t, *J* = 7.3 Hz, OCH_2C*H*(Fmoc)), 4.08 (1 H, dt, *J* = 9.5, 4.0 Hz, H-5), 3.89 (3 H, s, NCH_3); ^{13}C NMR (100 MHz, $CDCl_3$) *δ* 165.9, 165.4, 165.3 (3 x CO (Bz)), 154.94 (CO (Fmoc)), 143.4, 143.3, 141.4, 141.4, 138.6 (C_{Ar}), 137.33 (NCHN), 132.1, 130.0, 129.9, 129.9, 129.2, 129.2, 128.9, 128.7, 128.6, 128.5, 128.1, 127.4, 125.4, 125.3 (C_{Ar}), 123.6 (N*C*HCHN), 121.9 (NCH*C*HN), 120.2 (C_{Ar}), 100.2 (C-1), 72.9 (C-3), 72.4 (C-5), 71.9, 70.4, 70.3, 69.6, 66.3, 53.4, 46.8, 36.7; *m/z* (ESI-HRMS) $C_{54}H_{47}N_2O_{11}^+$ $[M - OTf]^+$ calcd: 899.3174; found: 899.3183.

4-(1-Methyl-3-methyleneimidazolium)benzyl 2-O-Acetyl-3,4-Di-O-benzyl-6-O-fluorenylmethoxycarbony-b-D-mannopyranoside trifluoromethanesulfonate (6a)

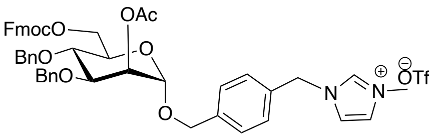

A solution of ITag acceptor **3b** (50 mg, 0.14 mmol) and glycosyl donor **2a** (221.3 mg, 0.28 mmol) in 1.5 mL of anhydrous MeCN was prepared in a vial which had been dried under vacuum, resulting in a solution of 0.095 M (acceptor) and 0.19 M (donor). Another vial was prepared with distilled trimethylsilyl trifluoromethanesulfonate (23 µL, 0.13 mmol) in 2.4 mL of anhydrous MeCN to make a 0.05 M solution of catalyst. The donor/acceptor solution and TMSOTf solution were each taken up in a syringe and installed onto syringe pumps connected to the microreactor chip and set to a 16.4

μL/min flow rate for each pump (overall flow rate was 32.8 μL/min). The reaction mixture was collected for a total of 94 min in a flask containing DCM and monitored by TLC-MS. After which time the reaction mixture solvent was removed under reduced pressure and the crude product was washed with hexane:Et_2O 1:1 (5 x 3 mL) then dried under reduced pressure. TLC-MS confirmed completion and then the solvent was evaporated under reduced pressure to yield **6a** as an oil (123 mg, 95%, a). ^{1}H NMR (400 MHz, $CDCl_3$) δ 9.20 (s, 1H, H_{Ar}), 7.40 – 7.26 (m, 11H, H_{Ar}), 5.42-5.39 (m, 2H, -CH_2), 4.95-4.51 (m, 6H), 4.02 (dd, J = 7.7, 3.3 Hz, 1H), 3.97 (d, J = 4.4 Hz, 3H), 3.54 – 3.46 (m, 1H), 3.18 (dt, J = 7.6, 4.5 Hz, 3H), 2.21 – 2.05 (m, 3H). ^{13}C HSQC NMR (101 MHz, $CDCl_3$) selected signals: δ 137.2, 129.1, 128.3, 128.3, 128.2, 128.2, 127.8, 123.3, 121.9, 97.3 (C-1), 77.8, 75.2, 74.1, 72.2, 71.7, 71.7, 68.8, 68.6, 62.0, 53.3, 50.7, 36.4, 20.9. HR-MS MALDI-TOF MS (ESI) calcd. for $C_{47}H_{47}N_2O_8^+$ [M - OTf]$^+$: 767.3327; found: 767.3320.

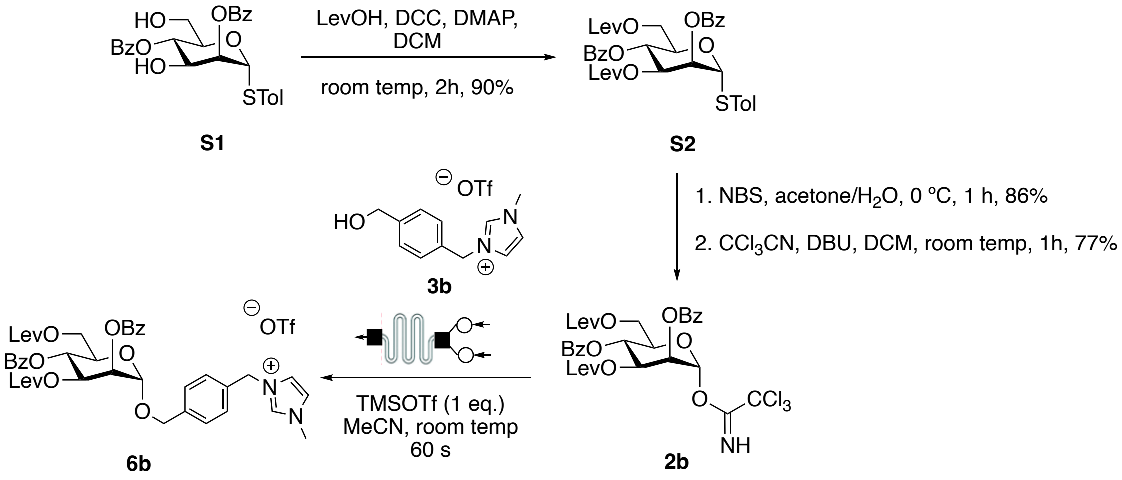

Scheme S3. Synthesis of 6b

***p*-Methylphenyl 2,4-di-*O*-benzoyl-3,6-di-*O*-levulinoyl-α-D-mannopyranoside (S2)**

To a solution of 1,3-dicyclohexylcarbodiimide (3.85 g, 18.45 mmol) in DCM (51 mL) cooled to 0 ºC, levulinic acid (3.8 mL, 36.37 mmol) was added. After stirring for 5 min at room temperature, the mixture was cooled to 0 ºC and the resulting solid generated was filtered and washed with DCM (10 mL). To the resulting solution, glycosyl thiol **S1**[4] (1.52 g, 3.08 mmol) and DMAP (20 mg, 0.16 mmol) were added. The reaction mixture was stirred at room temperature for 2 h, diluted with DCM (50 mL), and washed with sat. $NaHCO_3$ aq. soln. (100 mL) and H_2O (100 mL). The organic phase was dried over anh. $MgSO_4$, filtered and concentrated. The crude was purified by silica gel colun chromatography (toluene/acetone 20:1), giving monosaccharide **S2** as a white foam

(1.92 g, 90%). ^{1}H NMR (400 MHz, $CDCl_3$) δ 8.10 (d, J = 8.0 Hz, 2H, H_{Ar}), 8.03 (d, J = 8.0 Hz, 2H, H_{Ar}), 7.65 – 7.57 (m, 2H, H_{Ar}), 7.53 – 7.41 (m, 6H, H_{Ar}), 7.14 (d, J = 8.1 Hz, 2H, H), 5.81 – 5.78 (m, 1H, H-2), 5.75 (t, $^{3}J_{H4-H3}$ = $^{3}J_{H4-H5}$ = 10.1 Hz, 1H, H-4), 5.64 – 5.59 (m, 2H, H-3, H-1), 4.76 (ddd, $^{3}J_{H5-H4}$ = 10.0 Hz, $^{3}J_{H5-H6a}$ = 5.7 Hz, $^{3}J_{H5-H6b}$ = 2.5 Hz, 1H, H-5), 4.35 (dd, $^{3}J_{H6a-H6b}$ = 12.1 Hz, $^{3}J_{H6a-H5}$ = 5.6 Hz, 1H, H-6a), 4.25 (dd, $^{3}J_{H6b-H6a}$ = 12.2 Hz, $^{3}J_{H6b-H5}$ = 2.4 Hz, 1H, H-6b), 2.79 – 2.65 (m, 2H, $CH_{2,Lev}$), 2.63 – 2.46 (m, 4H, 2 x $CH_{2,Lev}$), 2.43 – 2.35 (m, 2H, $CH_{2,Lev}$), 2.33 (s, 3H, $CH_{3,Tol}$), 2.14 (s, 3H, $CH_{3,Lev}$), 1.94 (s, 3H, $CH_{3,Lev}$); ^{13}C NMR (101 MHz, $CDCl_3$) δ 206.4, 205.9, 172.4, 171.8, 165.7, 165.5 (C=O), 138.7 (C_{Ar}), 133.8 (C_{Ar}), 133.8 (C_{Ar}), 132.9 (C_{Ar}), 130.2, 130.1, 130.1, 129.4, 129.1, 129.0, 128.8, 128.7 (C_{Ar}), 86.3 (C-1), 71.7 (C-2), 69.9 (C-3), 69.7 (C-5), 67.3 (C-4), 63.1 (C-6), 38.0 ($CH_{2,Lev}$), 37.9 ($CH_{2,Lev}$), 30.0 ($CH_{3,Lev}$), 29.6 ($CH_{3,Lev}$), 28.0 ($CH_{2,Lev}$), 27.9 ($CH_{2,Lev}$), 21.3 ($CH_{3,Tol}$); MALDI-TOF MS (ESI) calcd. for $C_{20}H_{17}O_5\,Na^+$ $[M+Na]^+$: 360.09; found: 360.11

2,4-Di-O-benzoyl-3,6-di-O-levulinoyl-α-D-mannopyranosyl trichloroacetimidate (2b)

To a solution of glycosyl thiol **S2** (804 mg, 1.16 mmol) in a mixture of acetone/H_2O (9:1, 8 mL) cooled to 0ºC, NBS (1.03 g, 5.73 mmol) was added. After 1 h of stirring at 0ºC, the mixture was diluted with EtOAc (50 mL) and washed with sat. $NaHCO_3$ aq. soln. (50 mL). The aqueous phase was extracted with EtOAc (2 x 50 mL). The combined organic phases were dried over anh. $MgSO_4$, filtered and concentrated. The crude was purified by silica gel column chromatography (DCM/MeOH 100:1 → 60:1), giving the corresponding hemiacetal (585 mg, 86%) as a yellow oil. The hemiacetal was dissolved in dry DCM (6 mL) and the solution was cooled to 0ºC. Trichloroacetonitrile (0.6 mL, 5.86 mmol) and DBU (70 µL, 0.47 mmol) were added, and the reaction mixture was stirred at room temperature for 1 h. After solvent removal, the crude was purified by silica gel column chromatography (*n*-hexane/EtOAc 3:2) to yield trichloroacetimidate **2b** as a white foam (568 mg, 77%). ^{1}H NMR (500 MHz, $CDCl_3$) δ 8.84 (s, 1H, NH), 8.16 – 8.12 (m, 2H, H_{Ar}), 8.03 – 7.99 (m, 2H, H_{Ar}), 7.66 (t, J = 7.5 Hz, 1H, H_{Ar}), 7.59 (t, J = 7.5 Hz, 1H, H_{Ar}), 7.54 (t, J = 8.0 Hz, 2H, H_{Ar}), 7.46 (t, J = 8.0 Hz, 2H, H_{Ar}), 6.47 (d, $^{3}J_{H1-H2}$ = 2.0 Hz, 1H, H-1), 5.82 (t, $^{3}J_{H3-H4}$ = $^{3}J_{H4-H5}$ = 10.1 Hz, 1H, H-4), 5.76 (dd, $^{3}J_{H2-H3}$ = 3.3 Hz, $^{3}J_{H2-H1}$ = 2.0 Hz, 1H, H-2), 5.71 (dd, $^{3}J_{H3-H4}$ = 10.1 Hz, $^{3}J_{H3-H2}$ = 3.3 Hz, 1H, H-3), 4.43 – 4.37 (m, 1H, H-5), 4.32 – 4.29 (m, 2H, H-6), 2.81 – 2.66 (m, 2H, $CH_{2,Lev}$), 2.61 – 2.46 (m, 4H, 2 x $CH_{2,Lev}$), 2.43 – 2.36 (m, 2H, $CH_{2,Lev}$), 2.15 (s, 3H, $CH_{3,Lev}$), 1.93 (s, 3H, $CH_{3,Lev}$); ^{13}C NMR (126 MHz, $CDCl_3$) δ

206.4 (CO_{Lev}), 205.9 (CO_{Lev}), 172.4 (CO_{Lev}), 171.8 (CO_{Lev}), 165.6 (CO_{Bz}), 165.3 (CO_{Bz}), 160.0 (CN), 134.0, 133.8, 130.1, 130.1, 129.0, 128.9, 128.9, 128.7 (C_{Ar}), 94.6 (C-1), 90.7 (CCl_3), 71.4 (C-5), 69.2 (C-3), 68.6 (C-2), 66.3 (C-4), 62.7 (C-6), 38.0 ($CH_{2,Lev}$), 37.8 ($CH_{2,Lev}$), 29.9 ($CH_{3,Lev}$), 29.5 ($CH_{3,Lev}$), 28.0 ($CH_{2,Lev}$), 27.9 ($CH_{2,Lev}$); HRMS (ESI) calcd. for $C_{32}H_{32}NCl_3O_{12}Na$ $[M+Na]^+$: 750.0888; found: 750.0898.

4-(1-Methyl-3-methyleneimidazolium)benzyl 2,4-di-O-Benzoyl-3,6-di-O-levulonyl-b-D-mannopyranoside trifluoromethanesulfonate (6b)

A solution containing ITag acceptor **3b** (5.7 mg, 16.18 µmol) and trichloroacetimidate **2b** (27.4 mg, 37.59 µmol) in dry MeCN (0.25 mL) was loaded onto a syringe. A second syringe with TMSOTf (2.9 µL, 15.82 µmol) in MeCN (0.25 mL) was connected to the system. 16.4 µL/min flow rate for each pump (overall flow rate was 32.8 µL/min) was set, and the reaction mixture was collected in a flask with wet MeCN (1 mL) and evaporated. The crude was dissolved in H_2O (1 mL), washed with Et_2O (5 x 1 mL) and the aqueous phase was evaporated, to give **6b** as a brown syrup (12.3 mg, 83%, a). ^1H NMR (400 MHz, CD_3OD) δ 8.97 (s, 1H, H_{Ar}), 8.14 – 8.10 (m, 2H, H_{Ar}), 8.01 – 7.97 (m, 2H, H_{Ar}), 7.72 – 7.36 (m, 12H, H_{Ar}), 5.73 (t, $^3J_{H4-H3}$ = $^3J_{H4-H5}$ = 10.0 Hz, 1H, H-4), 5.58 (dd, $^3J_{H3-H4}$ = 10.1 Hz, $^3J_{H3-H2}$ = 3.3 Hz, 1H, H-3), 5.51 (dd, $^3J_{H2-H3}$ = 3.4 Hz, $^3J_{H2-H1}$ = 1.8 Hz, 1H, H-2), 5.43 (s, 2H, Ph-CH_2-Imidazole), 5.12 (d, $^3J_{H2-H1}$ = 1.7 Hz, 1H, H-1), under D_2O (1H, C*H*H-Ph), 4.74 (d, $^2J_{H-Hgem}$ = 12.4 Hz, 1H, CH*H*-Ph), 4.31 – 4.06 (m, 3H, H-5, H-6), 3.92 (s, 3H, CH_3), 2.82 – 2.76 (m, 2H, $CH_{2,Lev}$), 2.61 – 2.51 (m, 4H, $CH_{2,Lev}$), 2.34 – 2.28 (m, 2H, $CH_{2,Lev}$), 2.13 (s, 3H, $CH_{3,Lev}$), 1.84 (s, 3H, $CH_{3,Lev}$); ^{13}C NMR (126 MHz, CD_3OD) δ 209.3 (CO_{Lev}), 208.6 (CO_{Lev}), 174.0 (CO_{Lev}), 173.2 (CO_{Lev}), 167.0 (CO_{Bz}), 166.9 (CO_{Bz}), 139.7, 138.0, 135.2, 135.1, 134.8, 130.9, 130.8, 130.7, 130.6, 130.4, 130.3, 130.0, 130.0, 125.3, 123.8 (C_{Ar}), 98.5 (C-1), 71.5 (C-2), 71.0 (C-3), 70.7 (CH_2-Ph), 70.1 (C-5), 68.1 (C-4), 63.6 (C-6), 53.8 (Ph-CH_2-Imidazole), 38.6 ($CH_{2,Lev}$), 38.3 ($CH_{2,Lev}$), 36.5 (N^+-CH_3), 30.3 ($CH_{2,Lev}$), 29.0 ($CH_{2,Lev}$), 29.6 ($CH_{3,Lev}$), 29.4 ($CH_{3,Lev}$); *m/z* (ESI-HRMS) for $C_{42}H_{45}N_2O_{12}^+$ [M - OTf]$^+$ calcd: 769.2972; found: 769.2963.

***3-(3-Methylimidazolium)-1-propyl 2,3,4-O-benzoylester-2-deoxy-2-(2,2,2-trichloroethoxycarbonylamino)-β-D-glucopyranoside trifluoromethanesulfonate* (7).**

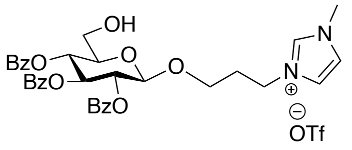

To a solution of **4b** (220 mg, 0.29 mmol) in CH_2Cl_2 (10 mL) was added HCl (1.25 M in MeOH, 20 eq) and left stirring at room temperature for 18 h. MALDI-TOF analysis showed completion of the reaction. The mixture was co-evaporated with toluene. CH_2Cl_2 was added to the residue and washed with H_2O (2x2 mL), dried over Na_2SO_4, filtered, concentrated under reduced pressure and dried under vacuum for 2 h to give **7** as a syrup (208 mg, 94%). ^{1}H NMR (400 MHz, $CDCl_3$) δ 8.45 (s, 1H), 7.97 – 7.87 (m, 4H, Ar), 7.79 – 7.74 (m, 2H, Ar), 7.62 – 7.56 (m, 2H, Ar), 7.55 – 7.49 (m, 1H, Ar), 7.49 – 7.41 (m, 4H, Ar), 7.40 – 7.33 (m, 2H, Ar), 7.29 (d, *J* = 1.6 Hz, 2H, Ar), 5.87 (t, 1H, $J_{3,4}$ = $J_{3,2}$ = 9.5 Hz, H-3), 5.53 (t,1H, $J_{4,3}$ = $J_{4,5}$ = 9.5 Hz, H-4), 5.37 (dd, 1H, $J_{2,1}$ = 8.0 Hz, $J_{2,3}$ = 9.5 Hz, H-2), 4.96 (d,1H, $J_{1,2}$ = 8.0 Hz, H-1), 4.25 – 4.10 (m, 2H, CH_2N), 3.96 (ddd, *J* = 10.0, 5.0, 2.4 Hz, 1H, H-5), 3.88 (ddd, *J* = 10.5, 7.3, 4.8 Hz, 1H, H-6a), 3.83 (s, 3H, NCH_3), 3.77 (dd, *J* = 12.5, 2.3 Hz, 1H, OCH_2CH_2), 3.75 – 3.66 (m, 2H, CH_2-6b, OCH_2CH_2), 2.28 (br, 1H, OH), 2.06 (qt, *J* = 6.5, 4.9 Hz, 2H, OCH_2CH_2). ^{13}C NMR (100 MHz, $CDCl_3$) *δ* 165.9, 165.6, 165., 137.1 (NCHN), 133.6, 133.4, 129.9, 129.7, 129.6, 129.0, 128.8, 128.5, 128.4, 128.3, 128.2, 125.3, 123.4, 122.6, 100.6 (C-1), 74.6 (C-5), 72.8 (C-3), 71.9 (C-2), 69.5 (C-4), 65.8 (C-6), 60.5 (OCH_2), 47.0 (CH_2N), 36.4 (NCH_3), 29.9 (OCH_2CH_2); *m/z* (HRMS-ESI) for $C_{34}H_{35}N_2O_9^+$ [M - OTf]$^+$ calcd: 615.2336; found: 615.2337.

3-(3-Methylimidazolium)-propyl 2,3,4-tri-O-benzoyl-6-O-(2,3,4,6-tetra-O-benzyl-D-glucopyranosyl)-β-D-glucopyranoside trifluoromethanesulfonate (8)

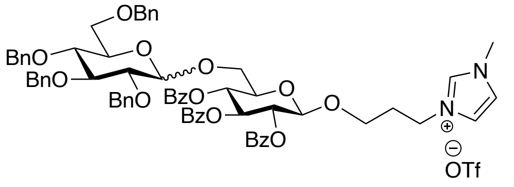

A solution containing ITag acceptor **7** (153 mg, 0.2 mmol) and glycosyl donor **1a** (274 mg, 0.4 mmol) in 1.0 mL of anhydrous MeCN was loaded on a syringe. A second syringe was loaded with trimethylsilyl trifluoromethanesulfonate (16.3 µL, 0.09 mmol) in 1.5 mL of anhydrous MeCN. An overall flow rate of 131.2 µL/min was set (each pump was set to a rate of 65.6 µL/min). The reaction solution was collected in a flask

containing triethylamine in DCM to quench the reaction. The crude product was washed with water (3 mL), then the water was extracted with DCM (2 x 15 mL). The dried residue was washed with Hexane:Et_2O 1:1 (3 x 5 mL) then dried under reduced pressure, before being passed through a silica plug with DCM:MeOH 93:7, yielding the title compound **8** as a solid (206 mg, 80 %, α:β 1 : 2.5). ^{1}H NMR (500 MHz, MeCN-*d*$_3$) *δ* 8.27 (1 H, s, NC*H*N β), 8.13 (1 H, s, NC*H*N α), 7.93 – 7.86 (m, H_{Ar}), 7.75 (m, H_{Ar}), 7.62 – 7.49 (m, H_{Ar}), 7.47 – 7.19 (m, H_{Ar}), 7.19 – 7.17 (m, 1H, NCHC*H*N β), 7.10 (t, 1H, *J* = 1.8 Hz, NCHC*H*N α), 5.86 (m, 2H), 5.62 – 5.52 (m, 2H), 5.33 (dd, 1H, *J* = 9.8, 8.0 Hz, H-2 β), 5.23 (dd, 1H, *J* = 9.7, 8.0 Hz, H-2 α), 4.99 – 4.94 (m, 2H, H-1' α), 4.92 – 4.87 (m, 2H, H-1 β, H-1 α), 4.86 – 4.73 (m, 5H), 4.72 – 4.65 (m, 2H), 4.54 (d, 1H, *J* = 10.9 Hz), 4.51 (d, 1H, *J* = 7.8 Hz, H-1' β), 4.50 – 4.44 (m, 4H), 4.39 (d, 1H, *J* = 11.9 Hz), 4.34 (d, 1H, *J* = 11.9 Hz), 4.22 – 4.13 (m, 4H), 4.09 (dd, 1H, *J* = 11.5, 2.5 Hz), 4.06 – 3.95 (m, 4H), 3.95 – 3.80 (m, 5H), 3.79 – 3.74 (m, 3H), 3.76 (s, 3H, NCH_3 β), 3.75 (s, 3H, NCH_3 α), 3.72 – 3.43 (m, 13H), 3.39 (dd, 1H, *J* = 10.9, 2.0 Hz), 3.32 (dd, 1H, *J* = 9.0, 7.8 Hz, H-2' β), 1.92 – 1.86 (m, 2H, $CH_2CH_2CH_2$); ^{13}C NMR (126 MHz, MeCN-*d*$_3$) *δ* 166.4, 166.3, 166.1, 166.0, 166.0, 139.8, 139.6, 139.5, 139.5, 139.4, 139.3, 136.9 (N*C*HN β), 136.9 (N*C*HN α), 134.7, 134.7, 134.6, 130.5, 130.4, 130.3, 130.1, 130.1, 130.1, 123.0, 129.9, 129.9, 129.9, 129.7, 129.6, 129.6, 129.5, 129.3, 129.3, 129.2, 129.2, 129.2, 129.0, 128.9, 128.8, 128.7, 128.7, 128.6, 128.5, 128.5, 128.5, 128.4, 124.4 (N*C*HCHN β), 124.3 (N*C*HCHN α), 123.4 (NCH*C*HN β), 123.3 (NCH*C*HN α), 104.5 (C-1' β), 101.2 (C-1 β, C-1 α), 97.4 (C-1' α), 85.1, 83.0, 82.4, 81.1, 79.0, 78.7, 76.0, 75.9, 75.6, 75.4, 75.4, 75.1, 74.5, 74.5, 74.0, 73.7, 73.7, 73.5, 73.3, 73.0, 73.0, 71.2, 70.6, 70.3, 70.0, 69.7, 68.8, 67.3, 66.9, 48.0, 47.7, 36.8 (NCH_3), 30.4 ($CH_2CH_2CH_2$); m/z (ESI-HRMS) $C_{68}H_{69}N_2O_{14}^+$ [M - OTf]$^+$ calcd: 1137.4743; found 1137.4731.

Microflow synthesis of trisaccharide 14

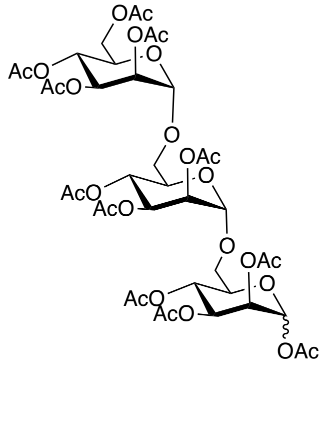

A solution of acceptor OH-**ITag2** **3b** (15 mg, 0.04 mmol) and glycosyl donor **2a** (67 mg, 0.08 mmol) in 0.75 mL of anhydrous MeCN was prepared in a vial which had been dried under vacuum. Another vial was prepared with distilled trimethylsilyl trifluoromethanesulfonate (7 µL, 0.038 mmol) in 1 mL of anhydrous MeCN. The donor/acceptor solution and TMSOTf solution were each taken up in a syringe and installed onto syringe pumps connected to the microreactor chip and set to a 16.4 µL/min flow rate for each pump (overall flow rate will be 32.8 µL/min). The reaction mixture was collected for a total of 94 min in a flask containing DCM and monitored by TLC-MS. After which time the reaction mixture solvent was removed under reduced pressure and the crude product was washed with hexane:Et_2O 1:1 (5 x 3 mL) then dried under reduced pressure to give **6a** as an light yellow oil. Without further purification, the dried mixture was redissolved in DMF (4 mL) and 10% of piperidine (0.4 mL) were added to the mixture and the reaction was stirred at room temperature for 20 min, after which time the solvent was removed under reduced pressure to quench the reaction, then the crude compound **9** was washed with toluene (2 x 5 mL) and then checked by TLC-MS and NMR before concentrating under reduced pressure. Without further purification, asolution of ITag-glycosyl acceptor **9** (~22 mg, ~0.03 mmol) and trichloroacetimidate **2a** (46 mg, 0.06 mmol) in anhydrous MeCN (1.6 mL) were loaded onto a syringe. A second syringe with distilled trimethylsilyl trifluoromethanesulfonate in DCM (4.9 µL, 0.03 mmol). A flow rate of 8.2 µL/min was set for each pump (overall rate will be 16.4 µL/min), and the reaction mixture was collected in a flask with DCM (1 mL) and monitored by TLC-MS before the solvent was evaporated and the crude product was washed with hexane:Et_2O 1:1 (5 x 1 mL) then dried with vacuum to yield ITagged-disaccharide **10** as an light yellow syrup. Without further purification, DMF (3.6 mL), 10% of piperidine (0.4 mL) were added to the crude dry mixture containing **10**, and the reaction was stirred at room temperature for 20 min, after which time the solvent was removed under reduced pressure to quench the reaction, then the crude compound **11** was washed with toluene (2 x 5 mL) and then checked by TLC-MS. The dried mixture containing **11** and glycosyl donor **3b** (46 mg, 0.06 mmol) and 1.6 mL of anhydrous DCM were loaded onto a syringe. Another syringe with distilled trimethylsilyl trifluoromethanesulfonate (4.8 µL, 0.03 mmol) was loaded onto a second syringe to make a 0.05 M solution in MeCN. A flow rate of 4.1 µL/min was set for each pump (8.2 µL/min overall). Formation of **12** was confirmed by TLC-MS and Maldi-TOF. The reaction mixture solvent was removed under reduced pressure and the crude product was washed with hexane:Et_2O 1:1 (5 x 3 mL) then dried with vacuum to yield **12** as an light yellow oil. Without further purification, the dried mixture containing **12** (23 mg, 0.01 mmol) was dissolved in DMF (270 µL) and

10% of piperidine (30 µL) was added. The reaction was stirred at room temperature for 20 min, after which time the solvent was removed under reduced pressure. The dried mixture was washed with toluene 2x times. TLC-MS and MALDI-TOF-MS confirmed the formation of **13**. The crude compound was filtered through a silica gel plug in a DCM:MeOH (95:5, v:v) mixture prior to ITag removal. I-Tagged trisaccharide **13** (~15 mg, 0.01 mmol) was dissolved in a mixture of MeOH/H_2O (1:1, v:v) and Pd/C (10% weight, 10 mg) was added. The reaction was stirred at room temperature under hydrogen atmosphere for 18 h. The mixture was then filtered through a nylon filter and concentrated under reduced pressure. The resulting dried mixture was then dissolved in a solution of pyridine (210 µL) and acetic anhydride (210 µL) and stirred at room temperature for another 16 h. The reaction was concentrated under reduced pressure and the product purified over silica gel column chromatography with a gradient with Hexane/EtOAc (10:0, v:v to 6:4, v:v) to yield **14** (4.4 mg, 11% yield over 8 steps). ^{1}H NMR (600 MHz, $CDCl_3$) *δ* 6.11 (d, *J* = 1.9 Hz, 2H, H-1), 5.89 (d, *J* = 1.2 Hz, 1H, H-1), 5.51 (dd, *J* = 3.3, 1.2 Hz, 2H, H-2), 5.39 – 5.37 (m, 4H, H-4), 5.32 (dd, *J* = 9.9 Hz, 2H, H-4), 5.29 (t, *J* = 2.3 Hz, 2H, H-2), 5.16 (dd, *J* = 9.9, 3.3 Hz, 2H, H-3), 4.32 (ddd, *J* = 14.8, 12.4, 5.1 Hz, 4H, H-6), 4.20 – 4.05 (m, 10H, H-6, H-5), 3.83 (ddd, *J* = 9.9, 5.3, 2.3 Hz, 2H, H.5), 2.24 (s, 3H, Ac), 2.21 (s, 3H, Ac), 2.20 (s, 3H, Ac), 2.13 (s, 3H, Ac), 2.12 (s, 6H, Ac), 2.08 (s, 6H, Ac), 2.07 (s, 3H, Ac), 2.04 – 2.03 (m, 6H, Ac); ^{13}C NMR (151 MHz, $CDCl_3$) *δ* 171.2, 170.7, 170.2, 170.0, 90.6, 90.4 (C1, C1’, C1’’), 78.2, 73.3, 70.7, 70.6, 68.8, 68.4, 68.2, 65.6, 65.4, 62.1, 62.1, 60.4, 29.7, 22.7, 22.7, 21.1, 20.9, 20.8, 20.8, 20.7, 20.7, 20.7, 20.7, 20.6, 14.2, 14.1. HRMS (ESI) m/z: calcd for $C_{40}H_{54}O_{27}Na^+$ $[M+Na]^+$ 966.2852, found 966.2848.

Microflow synthesis of pentasaccharide 18

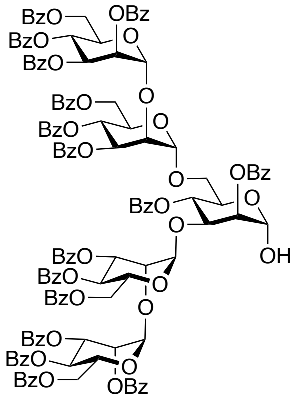

A solution containing ITag-glycosyl acceptor **3b** (13.9 mg, 39.45 µmol) and trichloroacetimidate **2b** (74.2 mg, 101.79 µmol) in dry MeCN (0.55 mL) was loaded onto a syringe. A second syringe with TMSOTf (7.2 µL, 39.38 µmol) in MeCN (0.55 mL) was connected to the system. A flow rate of 16.4 µL/min was set for each pump (32.8 µL/min overall flow rate), and the reaction mixture was collected in a flask with MeCN (1 mL) before the solvent was evaporated. The crude was redissolved in H_2O (1 mL), washed with Et_2O (5 x 1 mL) and the aqueous phase was evaporated. To a solution of the resulting crude in DCM (0.8 mL), a $N_2H_4 \cdot H_2O$ solution in pyridine/AcOH 3:2 (0.36 mL, 0.5 M) was added. The reaction mixture was stirred at room temperature for 2 h and concentrated *in vacuo*. The crude was dissolved in H_2O (1 mL), washed with Et_2O (5 x 1 mL) and the aqueous phase was evaporated. Without further purification, the crude mixture containing **15**, as confimed by TLC-MS and disaccharide **16**[4] (254 mg, 0.21 mmol) were dissolved in dry MeCN (0.9 mL) and loaded onto a syringe. A second syringe with TMSOTf (16 µL, 87.52 µmol) in dry MeCN (0.9 mL) was connected to the system. A flow rate of 16.4 µL/min was set for each pump (32.8 µL/min overall flow rate), and the reaction mixture was collected in a flask with wet MeCN (1 mL) before the solvent was evaporated and washed with Et_2O (5 x 1 mL). The dried mixture was then dissolved in toluene (14 mL) and added to a microwave (MW) vial containing Pd/C (232 mg) and an ammonium formate resin prepared as described by Gillard *et al.*[5] (5.6 g). The reaction mixture was stirred at 80ºC for 10 h under MW irradiation, filtered over Celite and concentrated. The crude was purified by silica gel column chromatography (toluene/EtOAc 14:1), yielding pentasaccharide **14** as a white foam (19 mg, 7.31 µmol, 19%, over 4 steps). ^{1}H NMR (400 MHz, $CDCl_3$) δ 8.24 – 8.21 (m, 2H, H_{Ar}), 8.14 – 8.10 (m, 4H, H_{Ar}), 8.04 – 7.99 (m, 6H, H_{Ar}), 7.97 – 7.93 (m, 7H, H_{Ar}), 7.93 – 7.84 (m, 9H, H_{Ar}), 7.84 – 7.77 (m, 4H, H_{Ar}), 7.60 – 7.27 (m, 47H, H_{Ar}), 7.19 – 7.13 (m, 3H, H_{Ar}), 6.96 (t, J = 7.7 Hz, 2H, H_{Ar}), 6.80 (t, J = 7.5 Hz, 1H, H_{Ar}), 6.09 (t, $^3J_{H4\text{-}H3}$ = $^3J_{H4\text{-}H}$ = 10.1 Hz, 1H, H-4), 6.04 – 5.89 (m, 5H), 5.88 – 5.80 (m, 3H), 5.75 (dd, $^3J_{H2\text{-}H3}$ = 3.5 Hz, $^3J_{H2\text{-}H1}$ = 1.6 Hz, 1H, H-2), 5.65 (dd, $^3J_{H3\text{-}H4}$ = 10.0 Hz, $^3J_{H3\text{-}H2}$ = 3.4 Hz, 1H, H-3), 5.56 (dd, $^3J_{H2\text{-}H3}$ = 3.2 Hz, $^3J_{H2\text{-}H1}$ = 2.0 Hz, 1H, H-2), 5.46 (d, $^3J_{H1\text{-}H2}$ = 1.5 Hz, 1H, H-1), 5.21 (d, $^3J_{H1\text{-}H2}$ = 1.5 Hz, 1H, H-1), 5.18 (d, $^3J_{H1\text{-}H2}$ = 1.5 Hz, 1H, H-1), 5.09 (d, $^3J_{H1\text{-}H2}$ = 1.6 Hz, 1H, H-1), 4.87 (d, J = 11.9 Hz, 1H, H-6), 4.71 – 4.64 (m, 2H), 4.64 – 4.37 (m, 9H, H-1), 4.36 – 4.30 (m, 2H), 4.25 – 4.18 (m, 2H), 4.03 – 3.96 (m, 2H), 3.63 (dd, J = 10.9, 2.5 Hz, H-6); ^{13}C NMR (101 MHz, $CDCl_3$) δ 166.5, 166.4, 166.3, 166.1, 166.1, 165.7, 165.7, 165.5, 165.5, 165.4, 165.2, 165.1, 165.1, 165.0, 165.0, 164.7, 138.1, 133.7 – 133.5, 133.4, 133.3, 133.2, 133.1, 133.0, 130.3, 130.2 – 129.8, 129.8, 129.5, 129.4, 129.4, 129.3, 129.2,

129.2, 129.0, 128.9, 128.8 – 128.3, 125.4, 100.2(C-1), 99.9(C-1), 99.6 (C-1), 98.5 (C-1), 96.7 (C-1), 78.5, 77.7, 75.6, 72.1, 70.8, 70.3, 70.1, 69.9, 69.9 – 69.7, 69.6, 69.6, 69.5, 69.4, 69.0, 67.4, 67.3, 66.8, 66.7, 66.5, 63.7, 62.9, 62.8; *m/z* (HRMS-ESI) for $C_{142}H_{116}O_{42}Na$ $[M+Na]^+$ calcd: 2515.6839; found: 2515.6824

References

[1] M. D. Bermejo, A. J. Kotlewska, L. J. Florusse, M. J. Cocero, F. van Rantwijk, C. J. Peters, *Green Chemistry* **2008**, *10*, 1049-1054.

[2] M. C. Galan, A. T. Tran, C. Bernard, *Chem Commun* **2010**, *46*, 8968-8970.

[3] A. T. Tran, R. Burden, D. T. Racys, M. C. Galan, *Chem Commun* **2011**, *47*, 4526-4528.

[4] J. Ramos-Soriano, M. C. de la Fuente, N. de la Cruz, R. C. Figueiredo, J. Rojo, J. J. Reina, *Org Biomol Chem* **2017**, *15*, 8877-8882.

[5] L. Gillard, A. T. Tran, F. D. Boyer, J. M. Beau, *Eur. J. Org. Chem.* **2016**, 1103–1109.

3-(3-Methylimidazolium)-1-propyl 2,3,4,6-tetra-O-benzyl-D-glucopyranoside trifluoromethanesulfonate (4a)

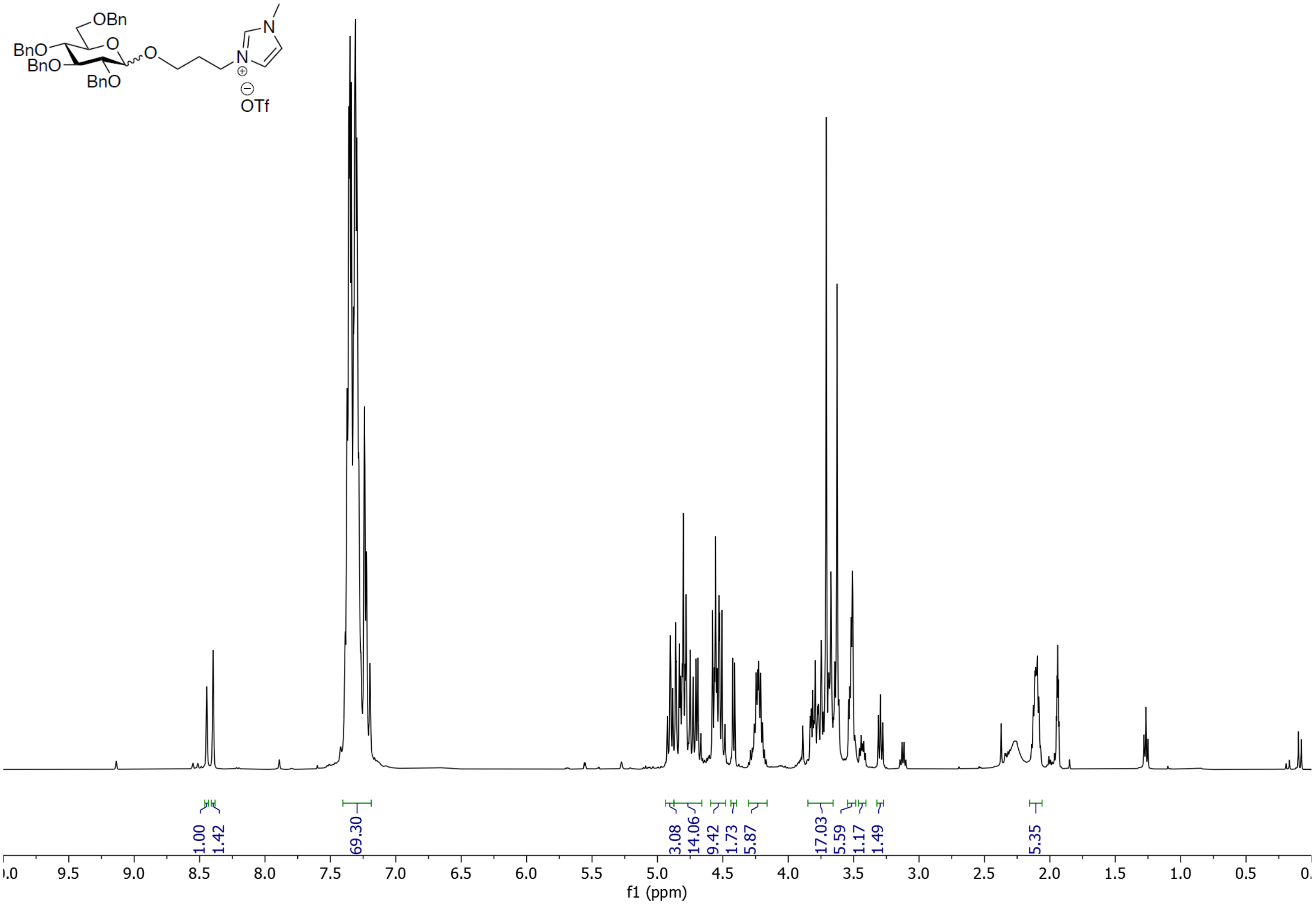

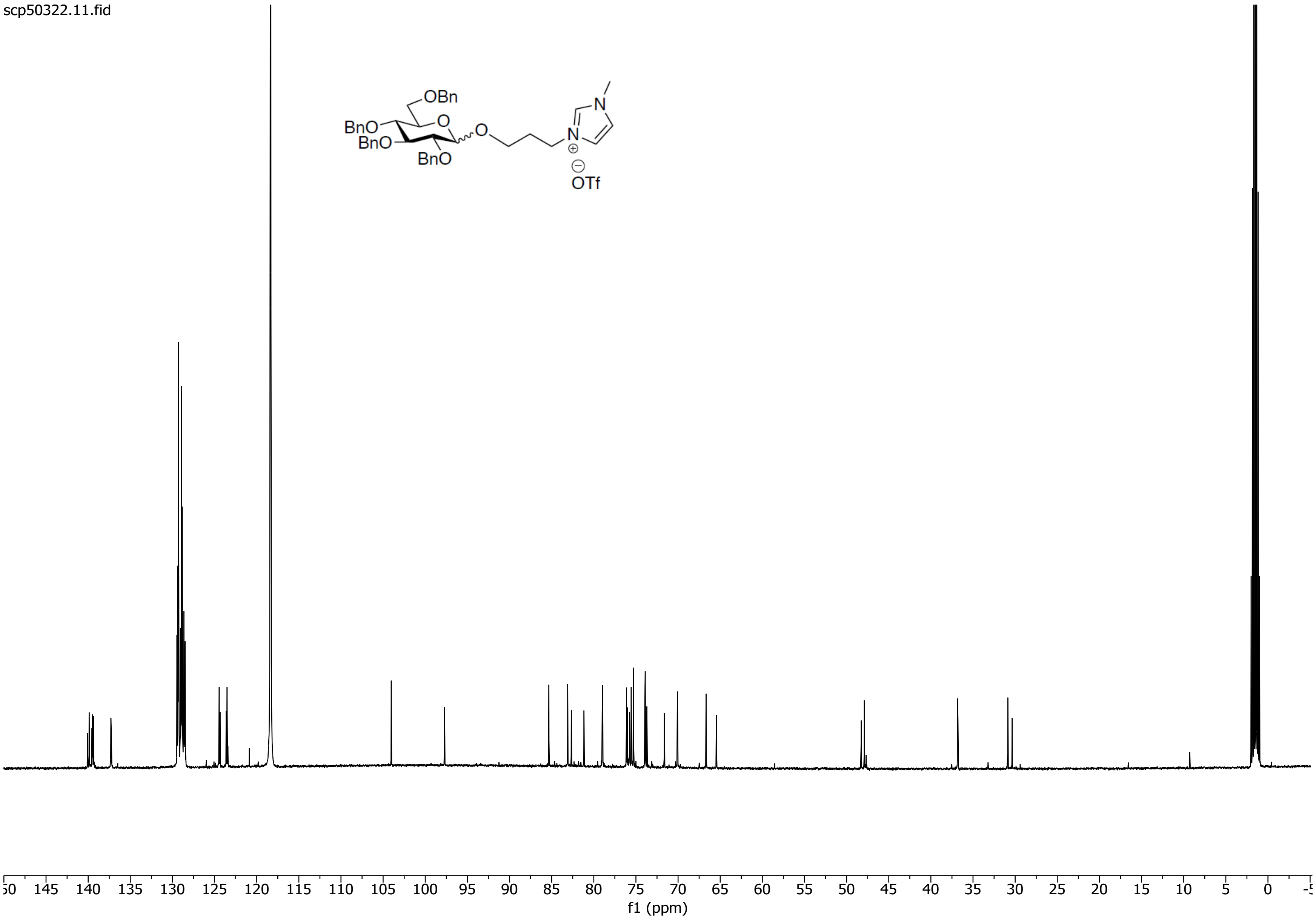
scp50322.11.fid
OBn
BnO
BnO
BnO
O
O
N
N
⊕
⊖
OTf
50 145 140 135 130 125 120 115 110 105 100 95 90 85 80 75 70 65 60 55 50 45 40 35 30 25 20 15 10 5 0 -5
f1 (ppm)

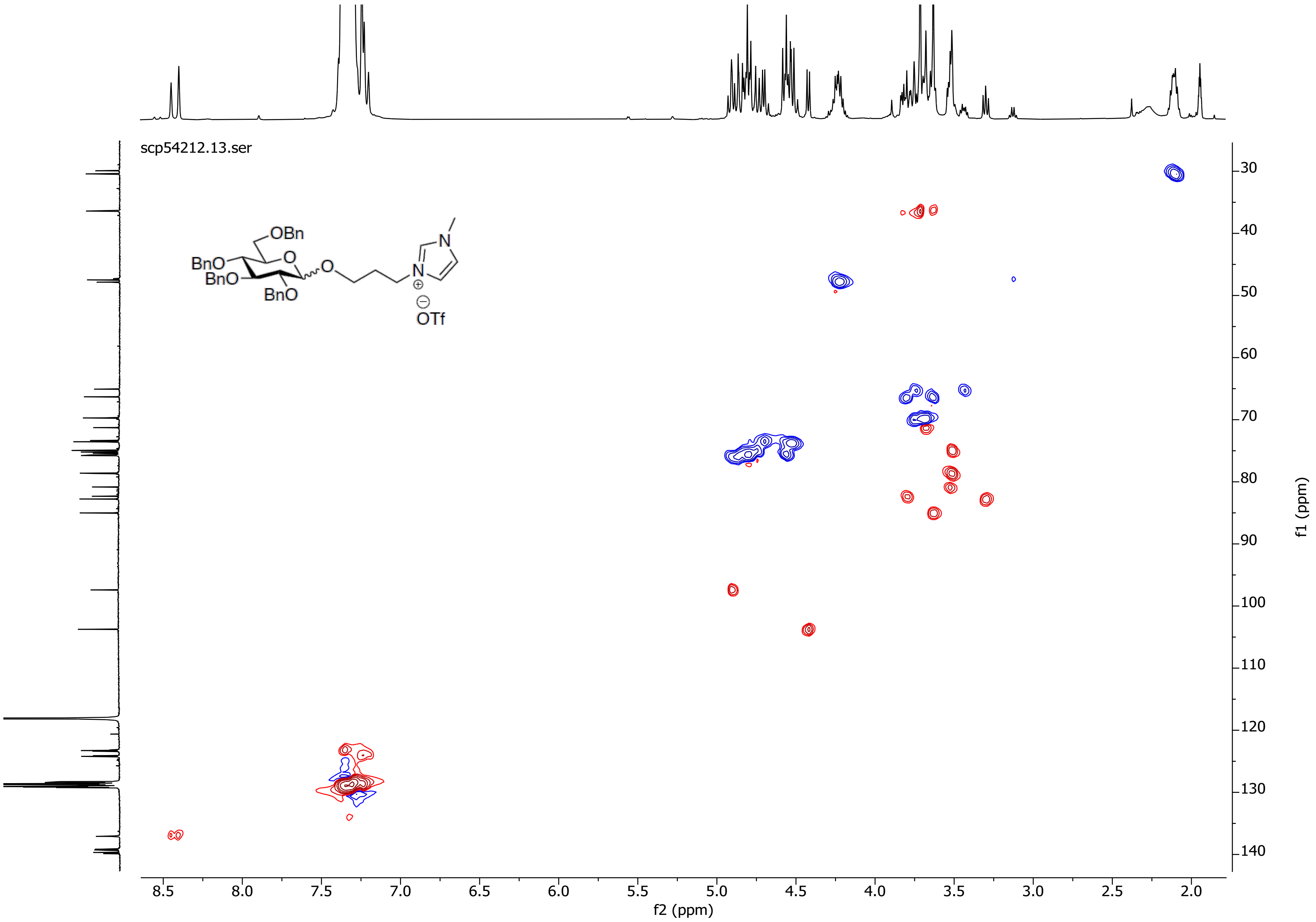
scp54212.13.ser
OBn
BnO
BnO
BnO
O
O
N
N
⊕
⊖
OTf
f2 (ppm)
f1 (ppm)
8.5
8.0
7.5
7.0
6.5
6.0
5.5
5.0
4.5
4.0
3.5
3.0
2.5
2.0
30
40
50
60
70
80
90
100
110
120
130
140

3-(3-Methylimidazolium)-1-propyl 6-O-chloroacetyl-2,3,4-tri-O-benzyl-β-D-glucopyranoside trifluoromethanesulfonate (4c)

cg/rw75789 RW-265-1st-ii
single_pulse

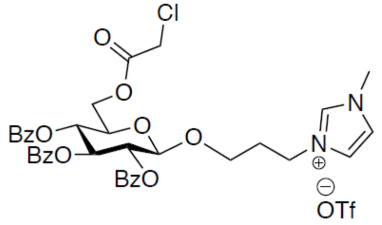

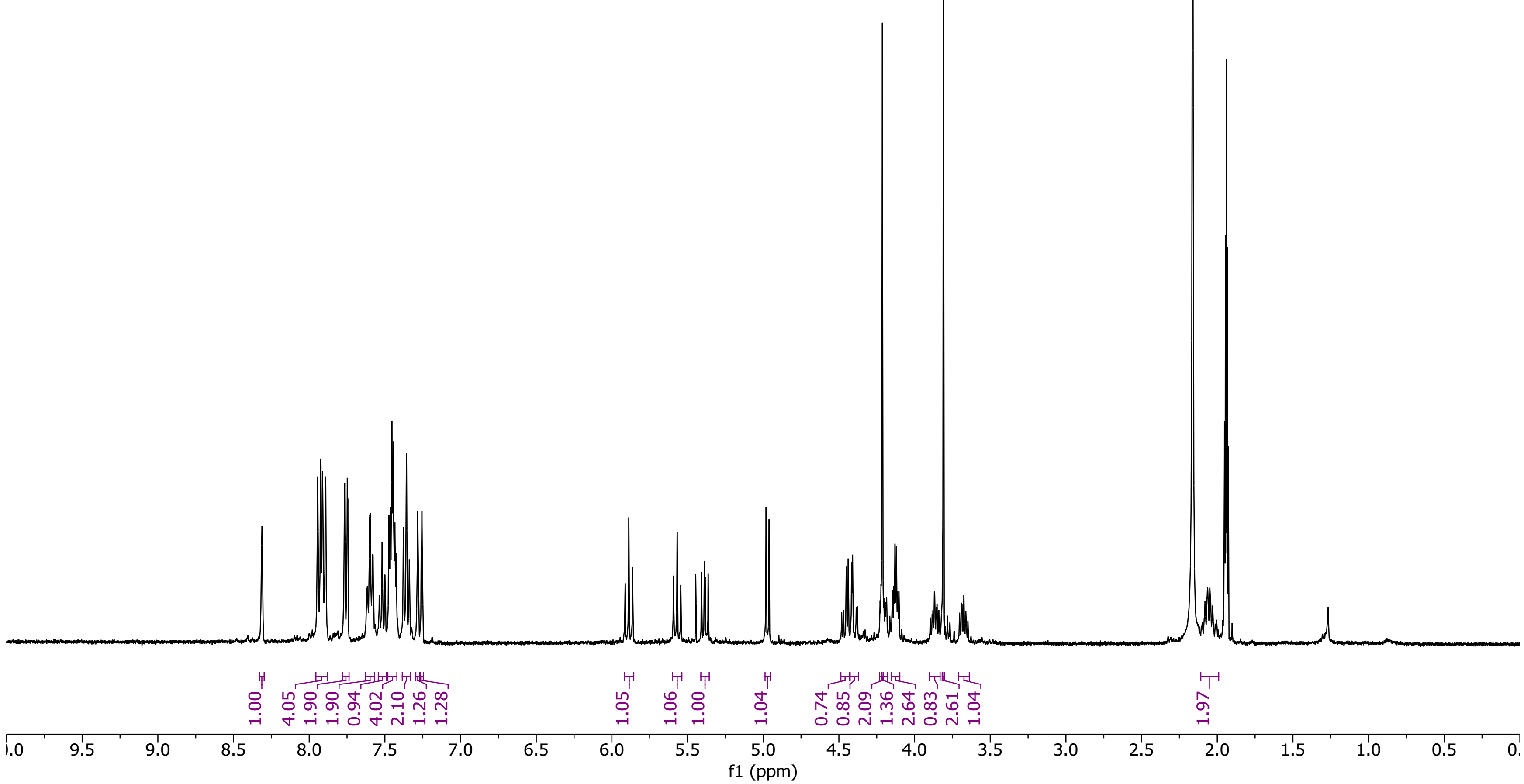

rw37889_RW-265-2nd-i_CARBON_01

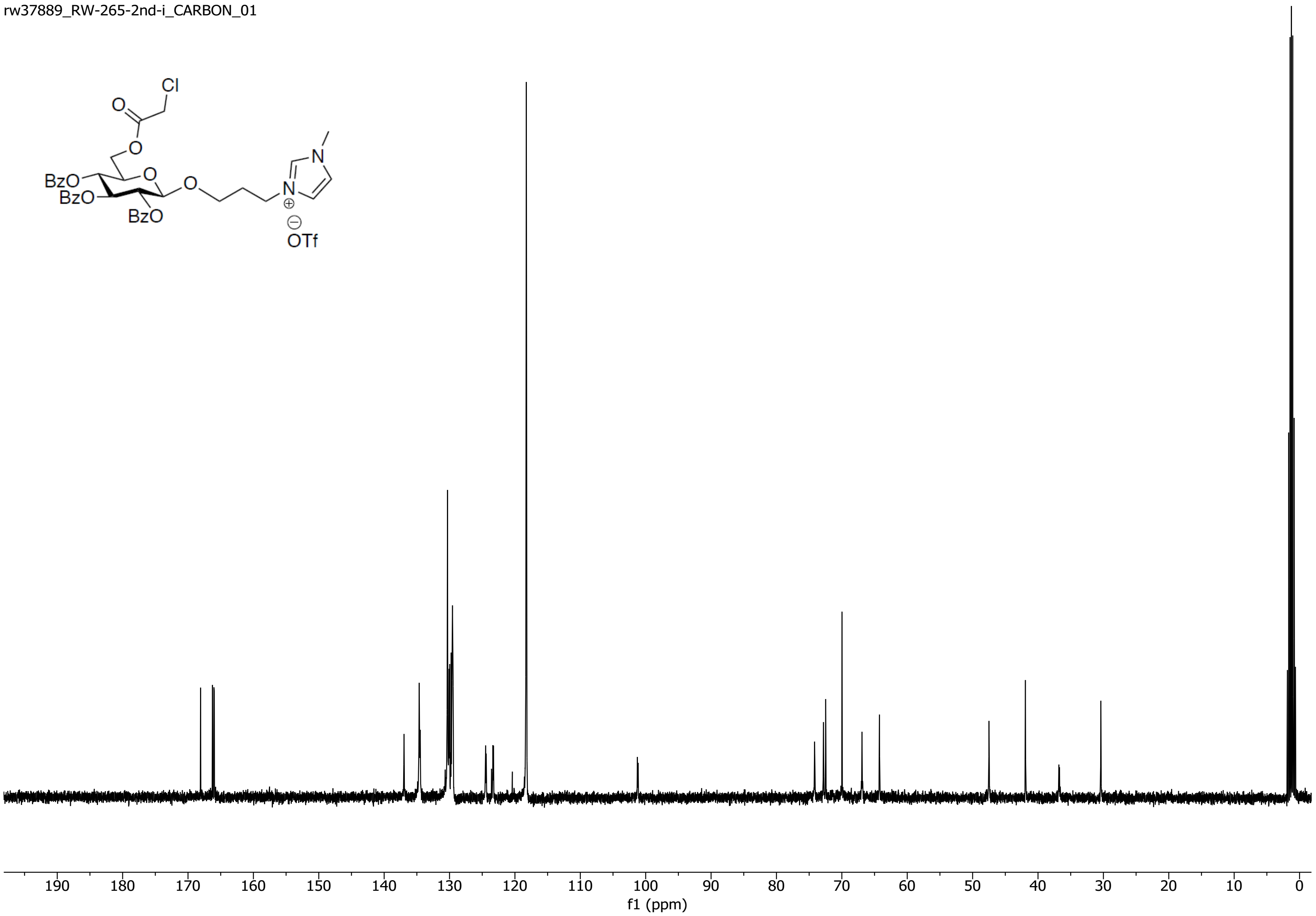

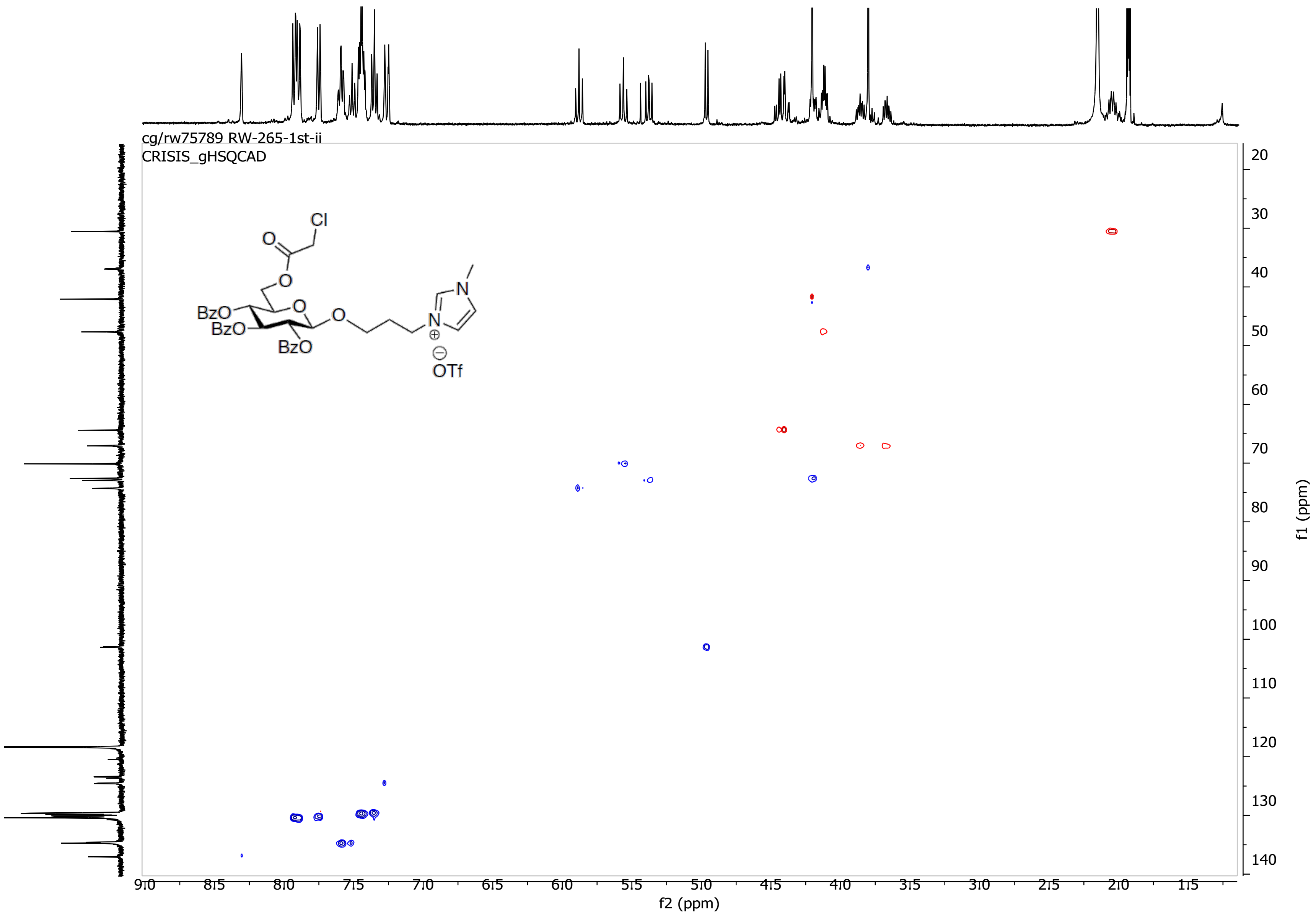

cg/rw75789 RW-265-1st-ii
CRISIS_gHSQCAD
Cl
O
O
BzO
BzO
BzO
O
O
N
N
OTf
f2 (ppm)
f1 (ppm)

3-(3-Methylimidazolium)-1-propyl 2-O-acetyl-3,4,6-tri-O-benzyl-β-D-glucopyranoside trifluoromethanesulfonate (4d)

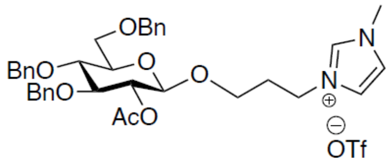

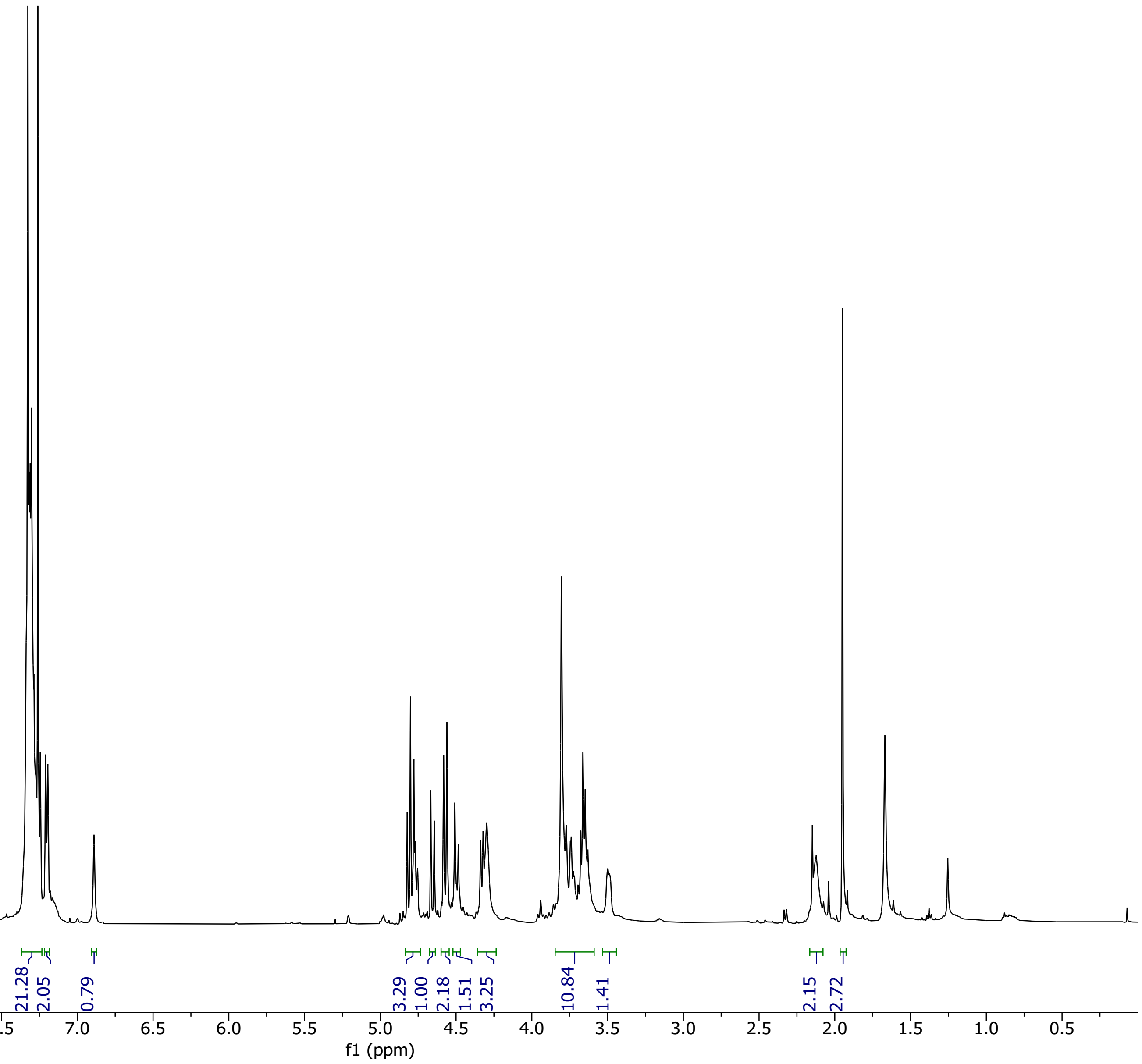

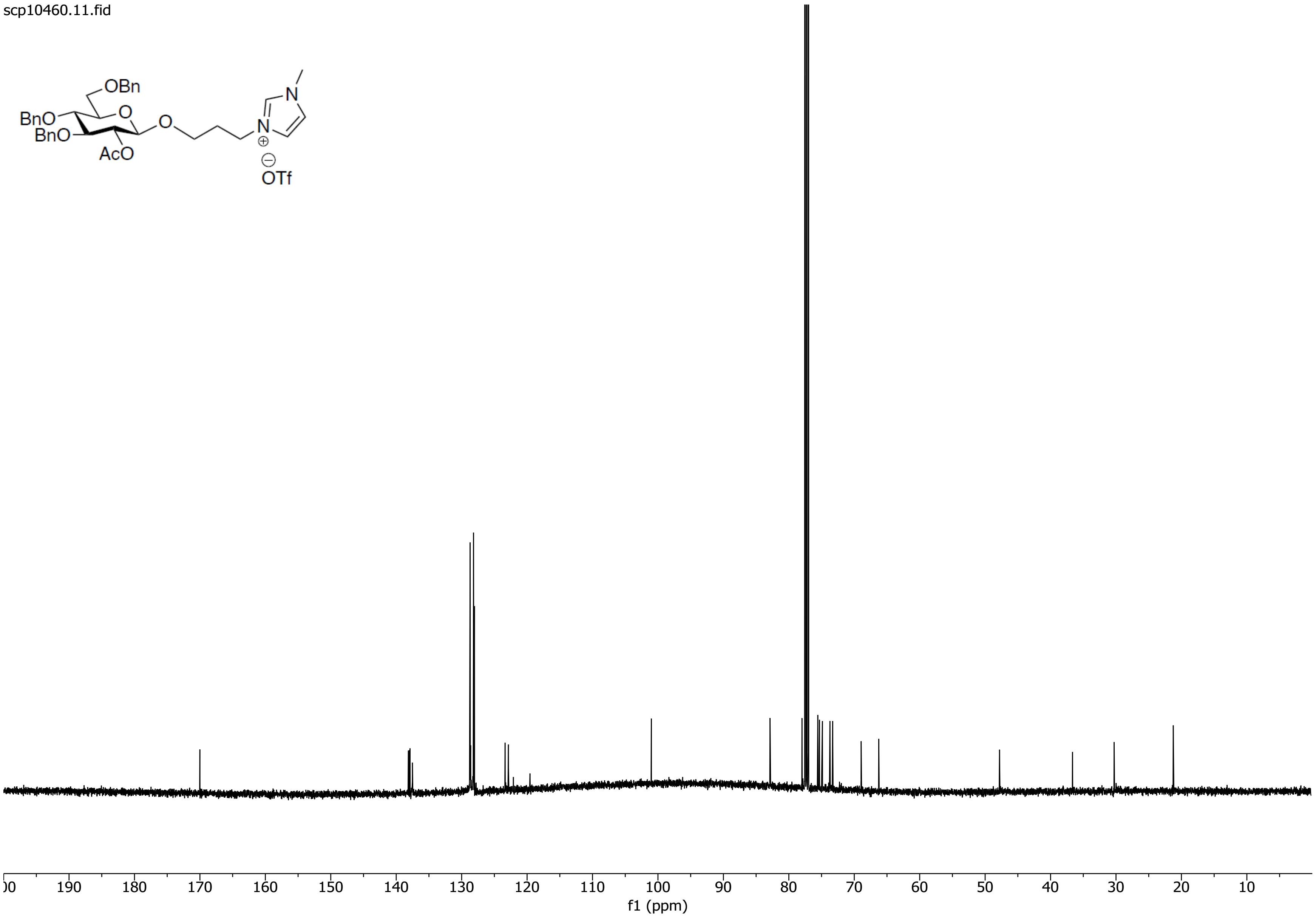
scp10460.11.fid
OBn
BnO
BnO
O
O
AcO
N
N
⊕
⊖
OTf
190 180 170 160 150 140 130 120 110 100 90 80 70 60 50 40 30 20 10
f1 (ppm)

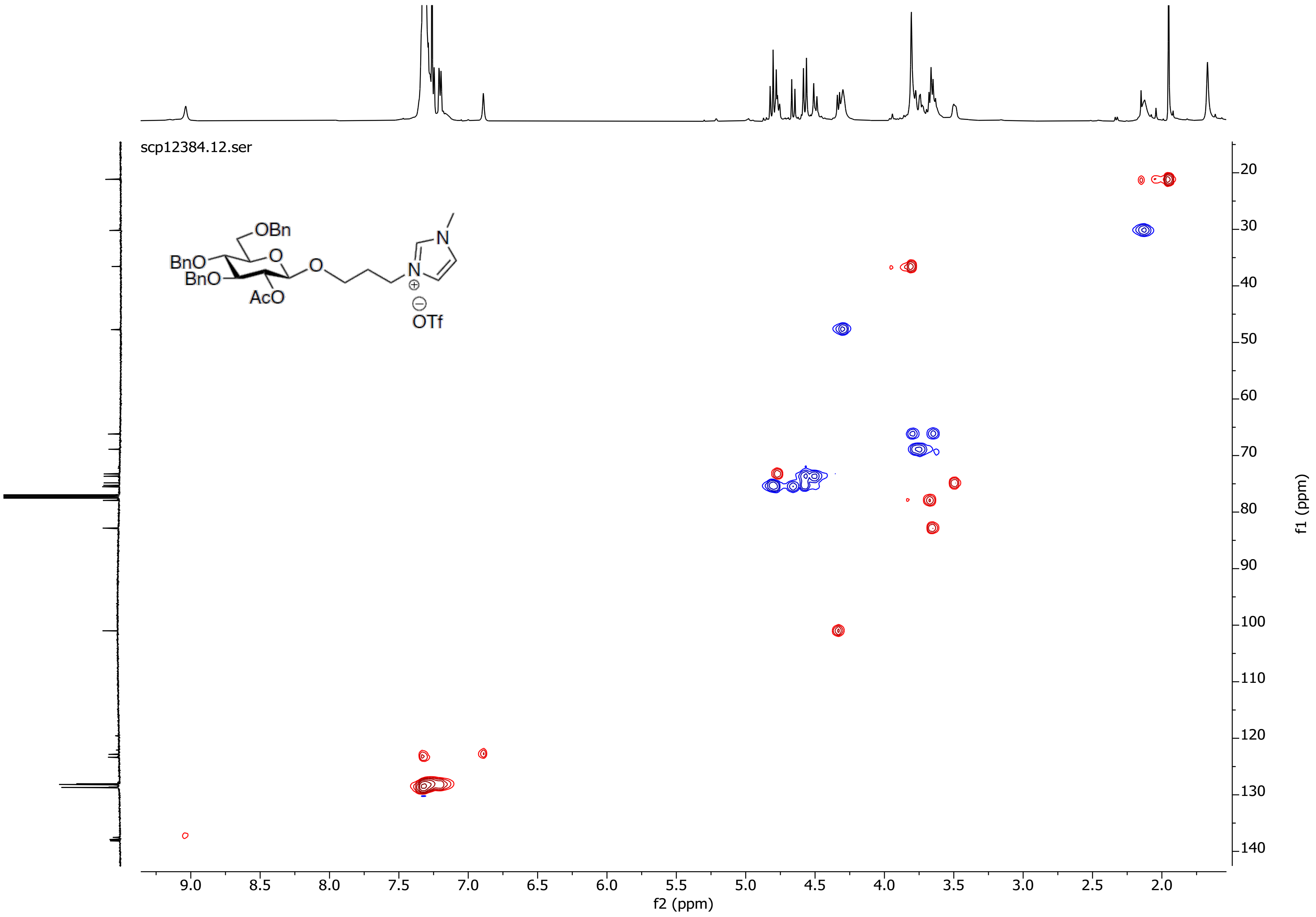

scp12384.12.ser
OBn
BnO
BnO
O
O
AcO
N
N
OTf
f2 (ppm)
f1 (ppm)

4-(1-Methyl-3-methyleneimidazolium)benzyl 2,3,4,6-tetra-O-benzyl-D-glucopyranoside trifluoromethanesulfonate (5a)

RW-221-1st-ii_PROTON_001

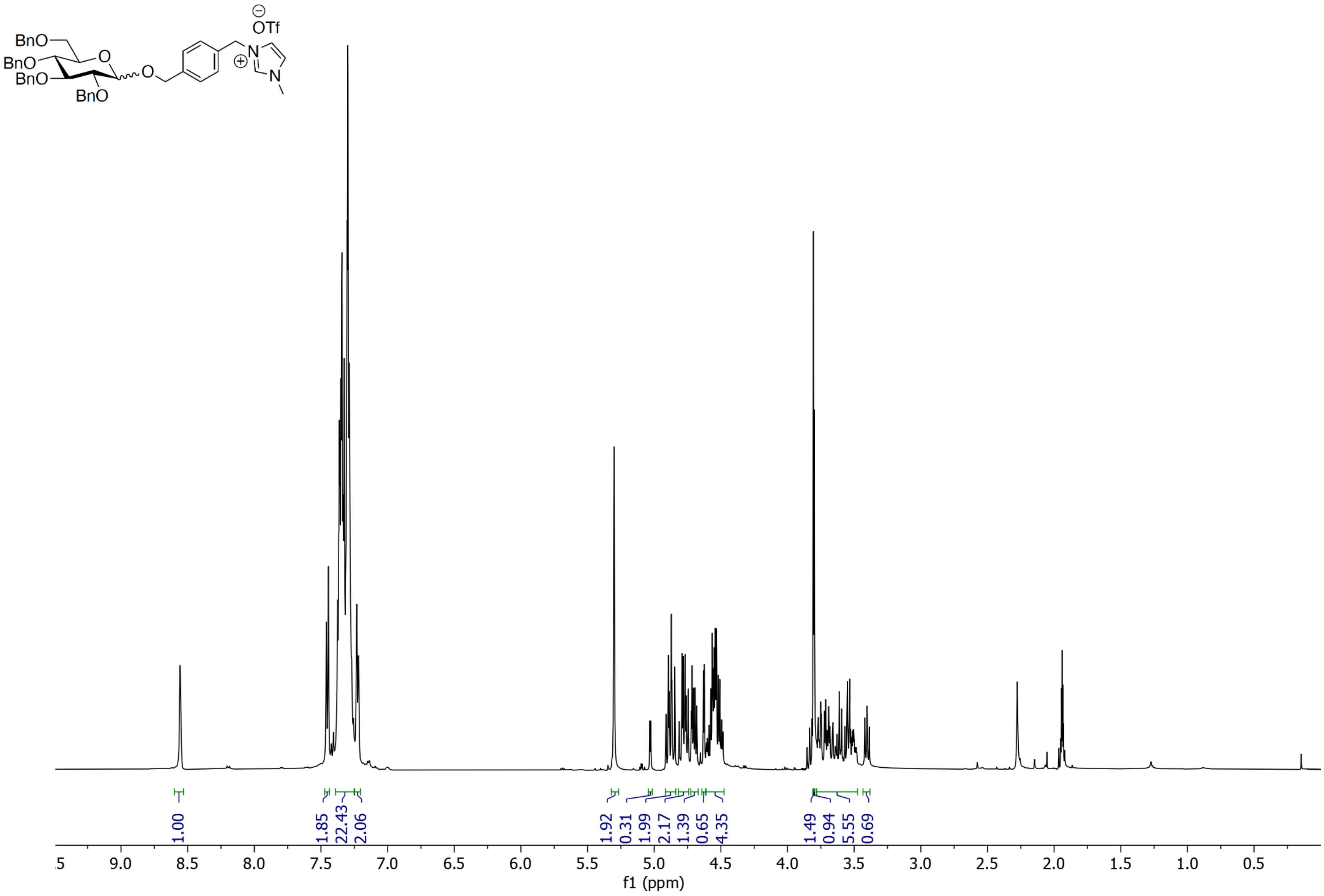

rw25657_RW-221-1st-ii_CARBON_001

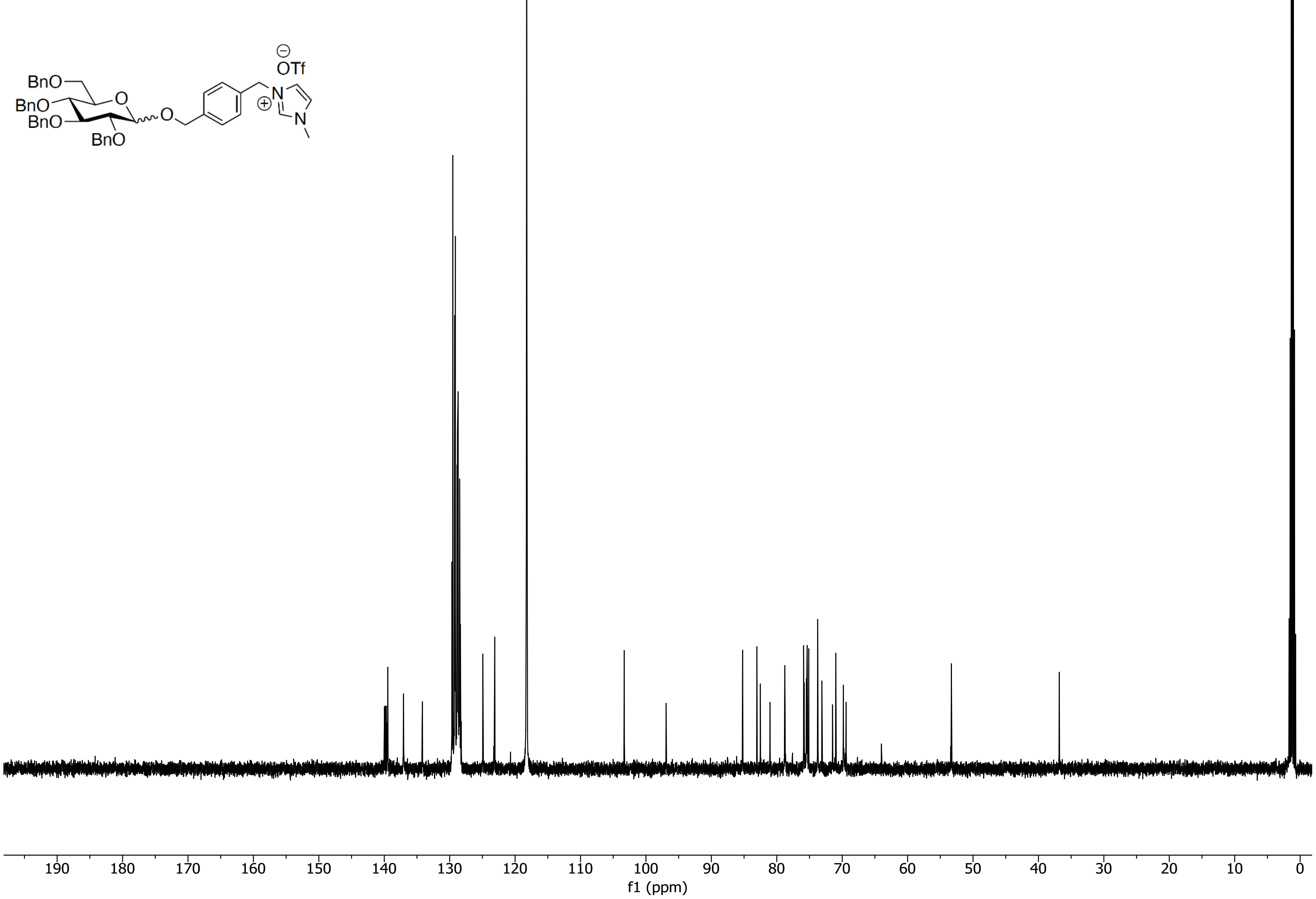

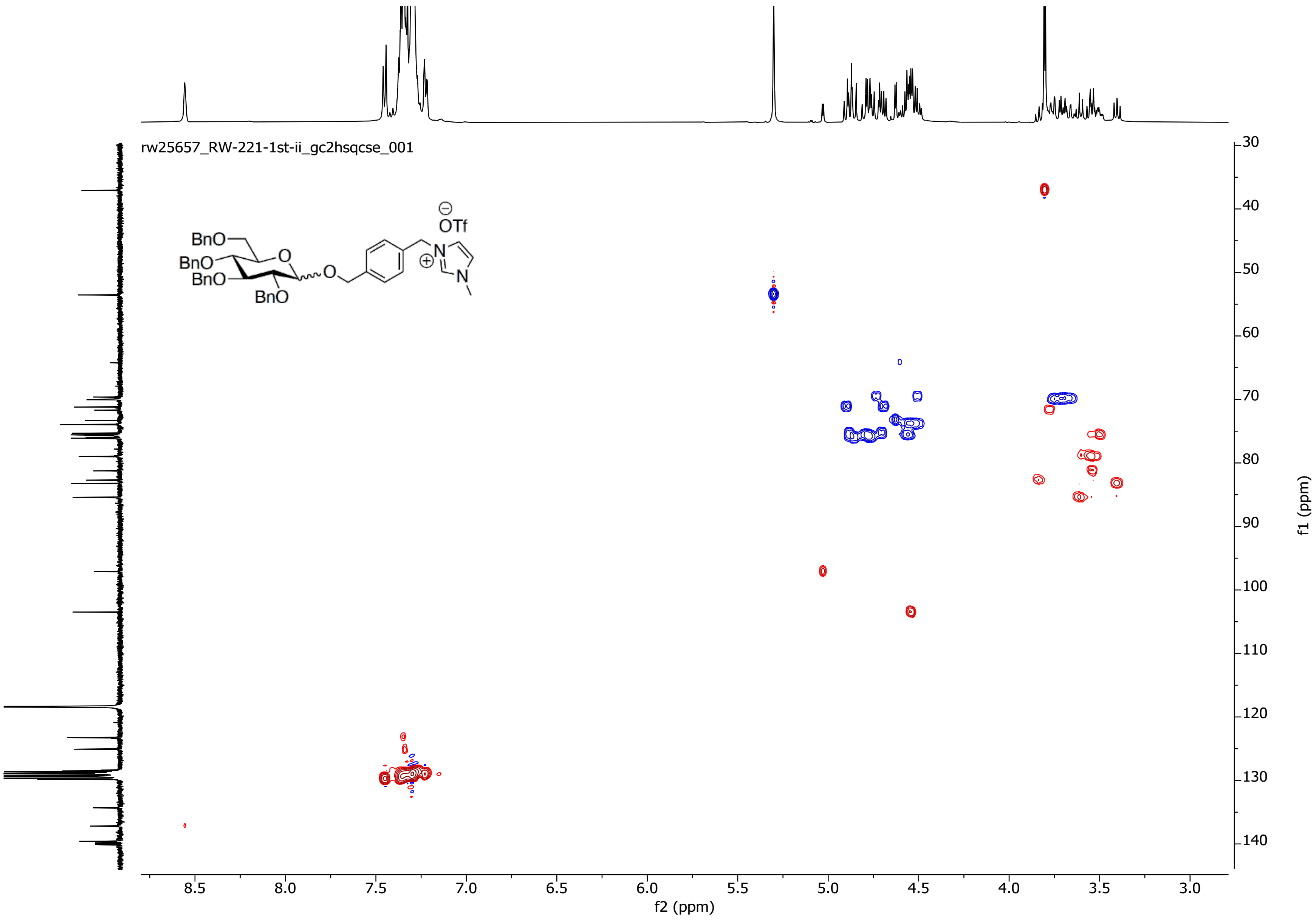
rw25657_RW-221-1st-ii_gc2hsqcse_001
BnO
BnO
BnO
BnO
O
O
N
N
OTf
f2 (ppm)
f1 (ppm)

4-(1-Methyl-3-methyleneimidazolium)benzyl 2,3,4-tri-O-benzoyl-6-O-chloroacetyl-β-D-glucopyranoside trifluoromethanesulfonate (5c)

rw17744_RW-282-9th-i_PROTON01

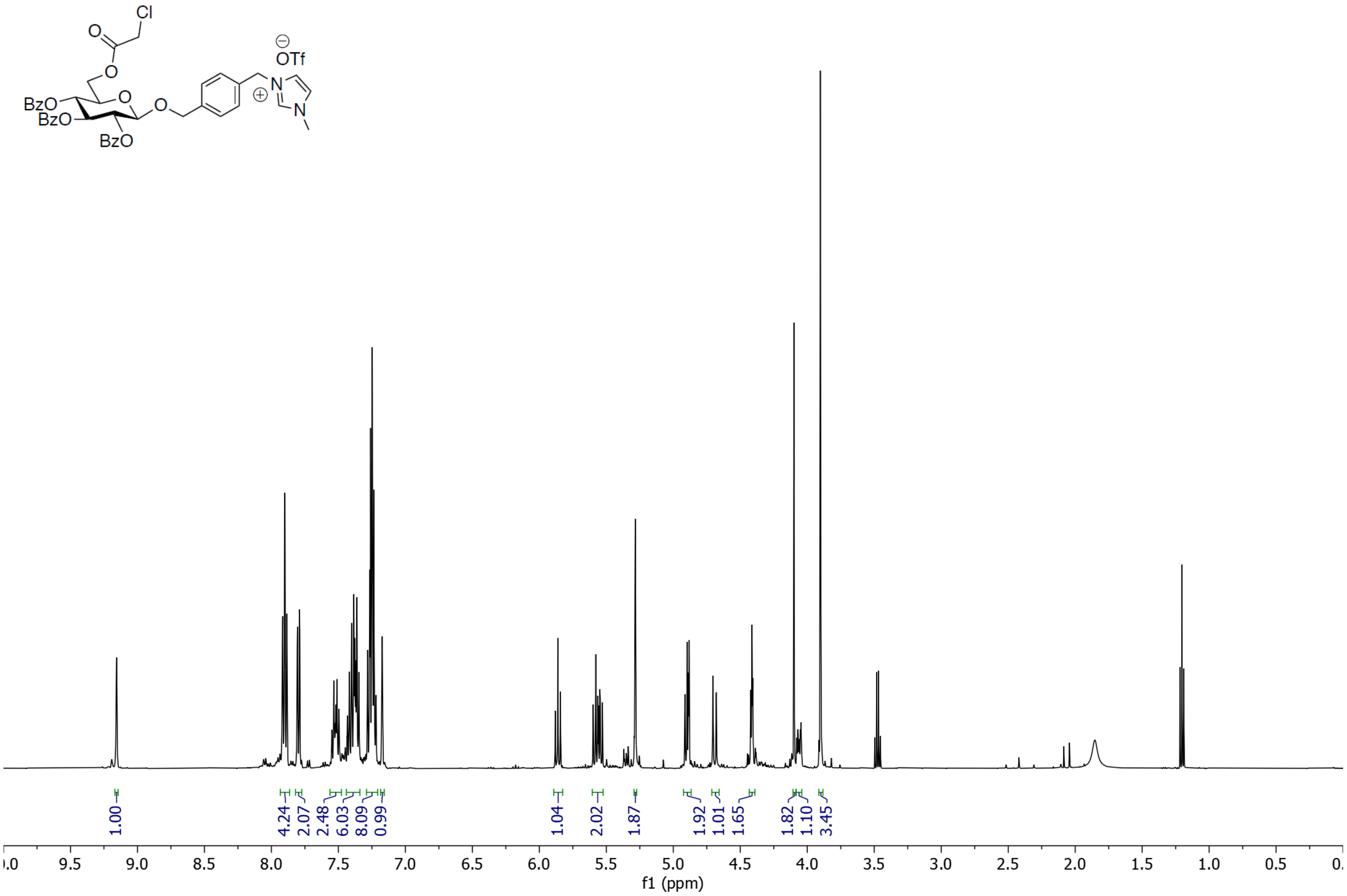

rw17744_RW-282-9th-i_CARBON01

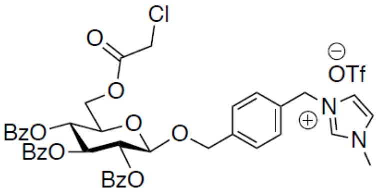

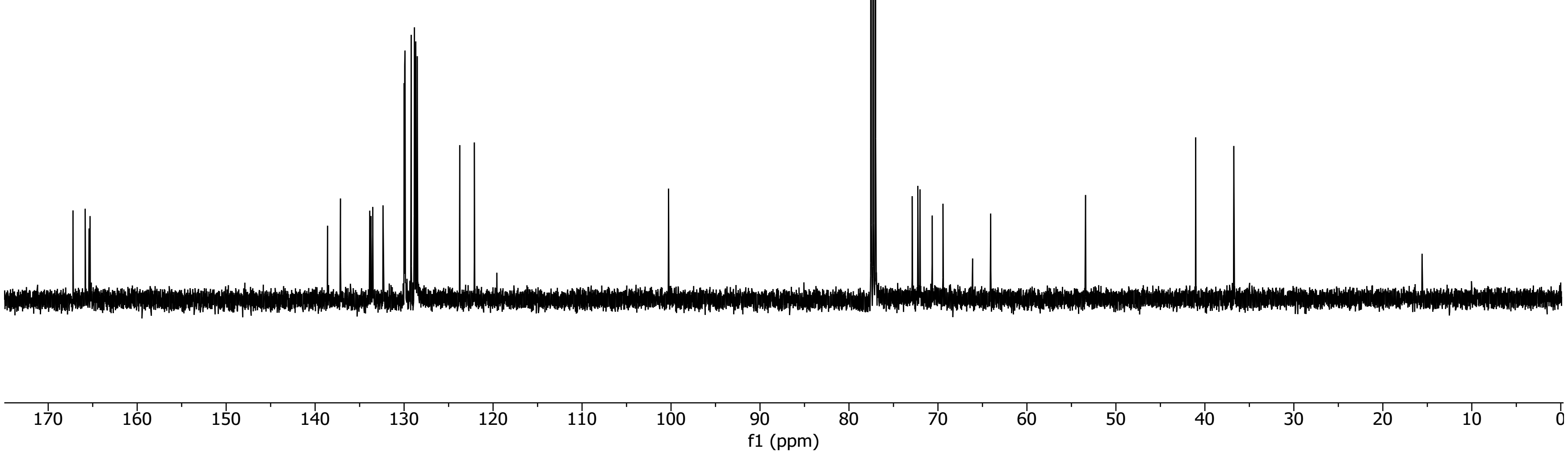

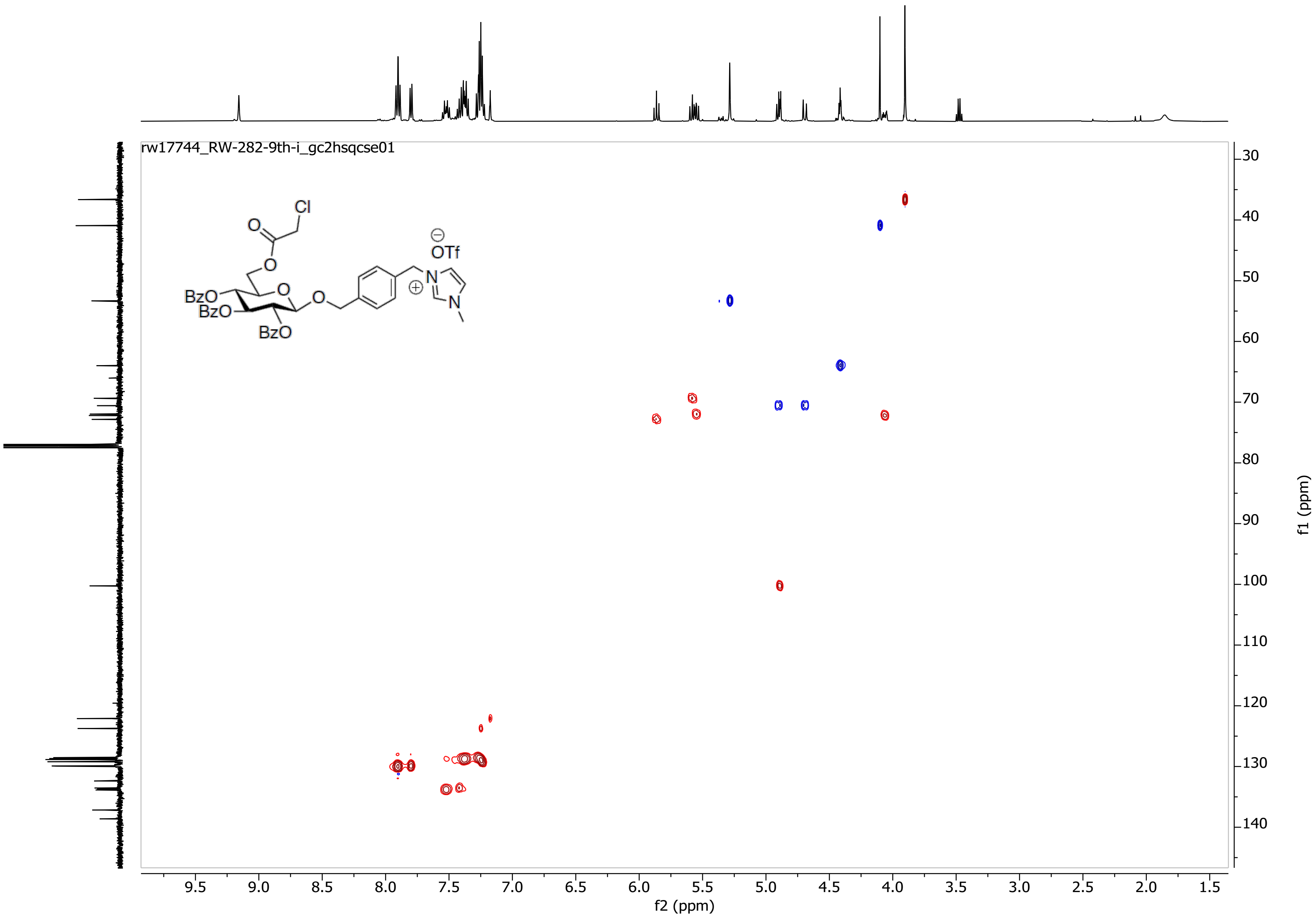
rw17744_RW-282-9th-i_gc2hsqcse01
Cl
O
O
⊖
OTf
BzO
O
O
N
BzO
BzO
⊕
N
f2 (ppm)
f1 (ppm)
9.5
9.0
8.5
8.0
7.5
7.0
6.5
6.0
5.5
5.0
4.5
4.0
3.5
3.0
2.5
2.0
1.5
30
40
50
60
70
80
90
100
110
120
130
140

4-(1-Methyl-3-methyleneimidazolium)benzyl 2,3,4-tri-*O*-benzoyl-6-*O*-(9-fluorenylmethoxycarbonyl)-β-D-glucopyranoside trifluoromethanesulfonate (5d)

rw17755_RW-333-1st-i_PROTON01

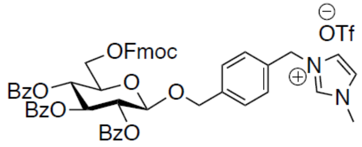

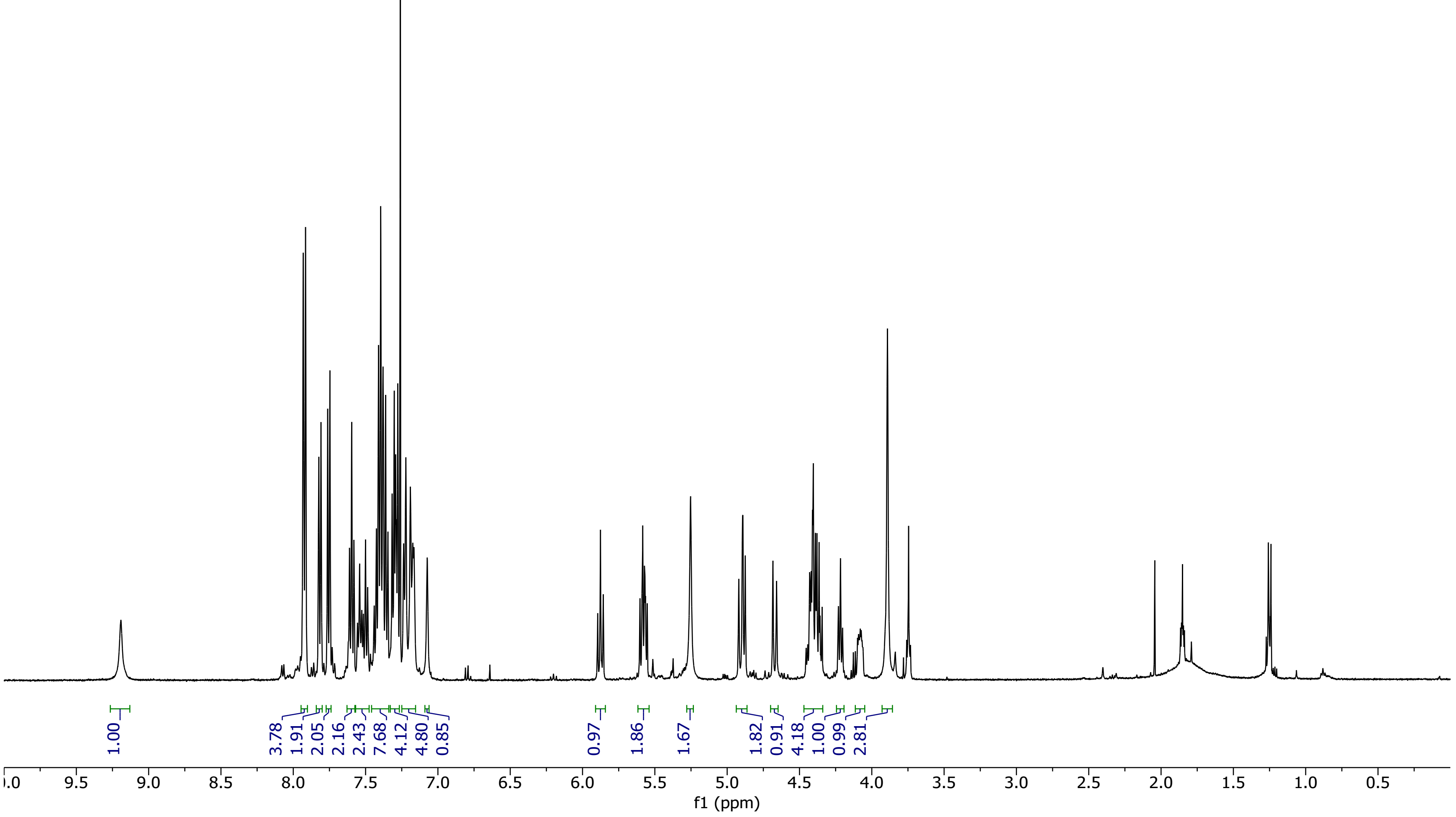

rw17755_RW-333-1st-i_CARBON01

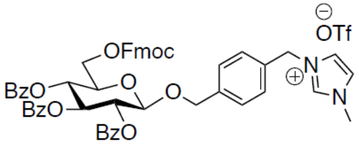

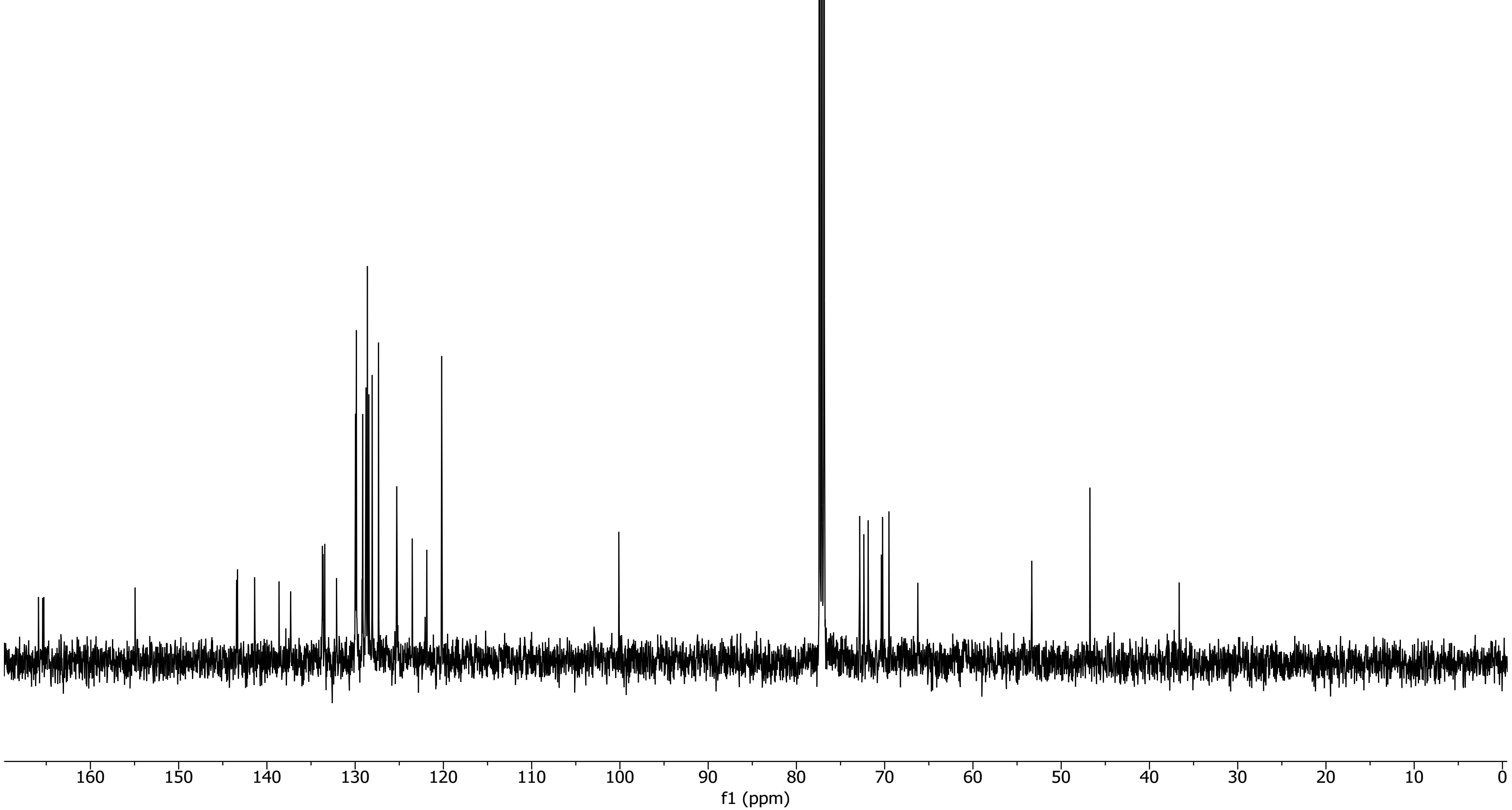

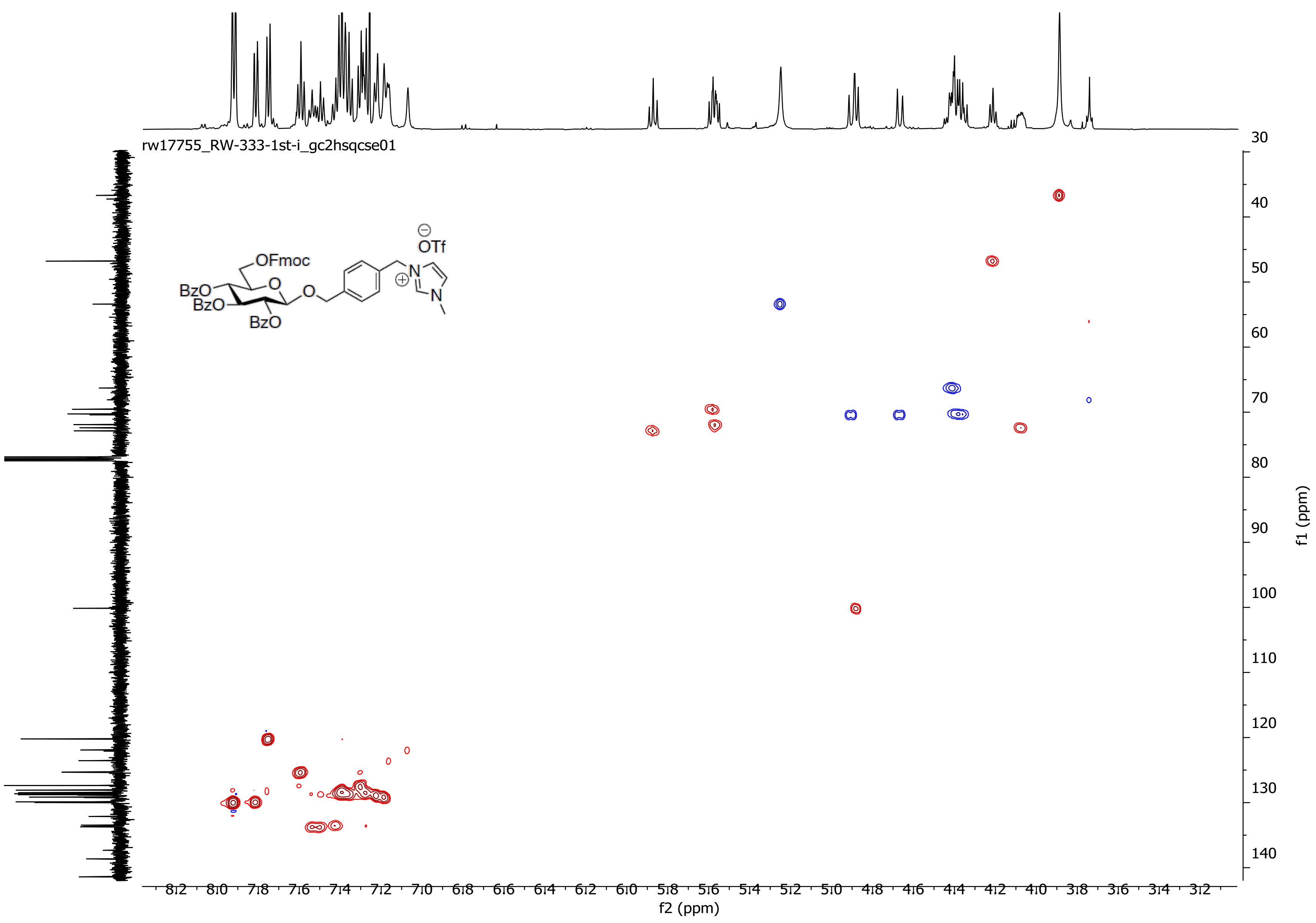
rw17755_RW-333-1st-i_gc2hsqcse01
OFmoc
BzO
BzO
BzO
O
O
N
N
OTf
f2 (ppm)
f1 (ppm)
8.2 8.0 7.8 7.6 7.4 7.2 7.0 6.8 6.6 6.4 6.2 6.0 5.8 5.6 5.4 5.2 5.0 4.8 4.6 4.4 4.2 4.0 3.8 3.6 3.4 3.2
30 40 50 60 70 80 90 100 110 120 130 140

4-(1-Methyl-3-methyleneimidazolium)benzyl 2-*O*-Acetyl-3,4-Di-*O*-benzyl-6-*O*-fluorenylmethoxycarbony-*β*-D-mannopyranoside trifluoromethanesulfonate (6a)

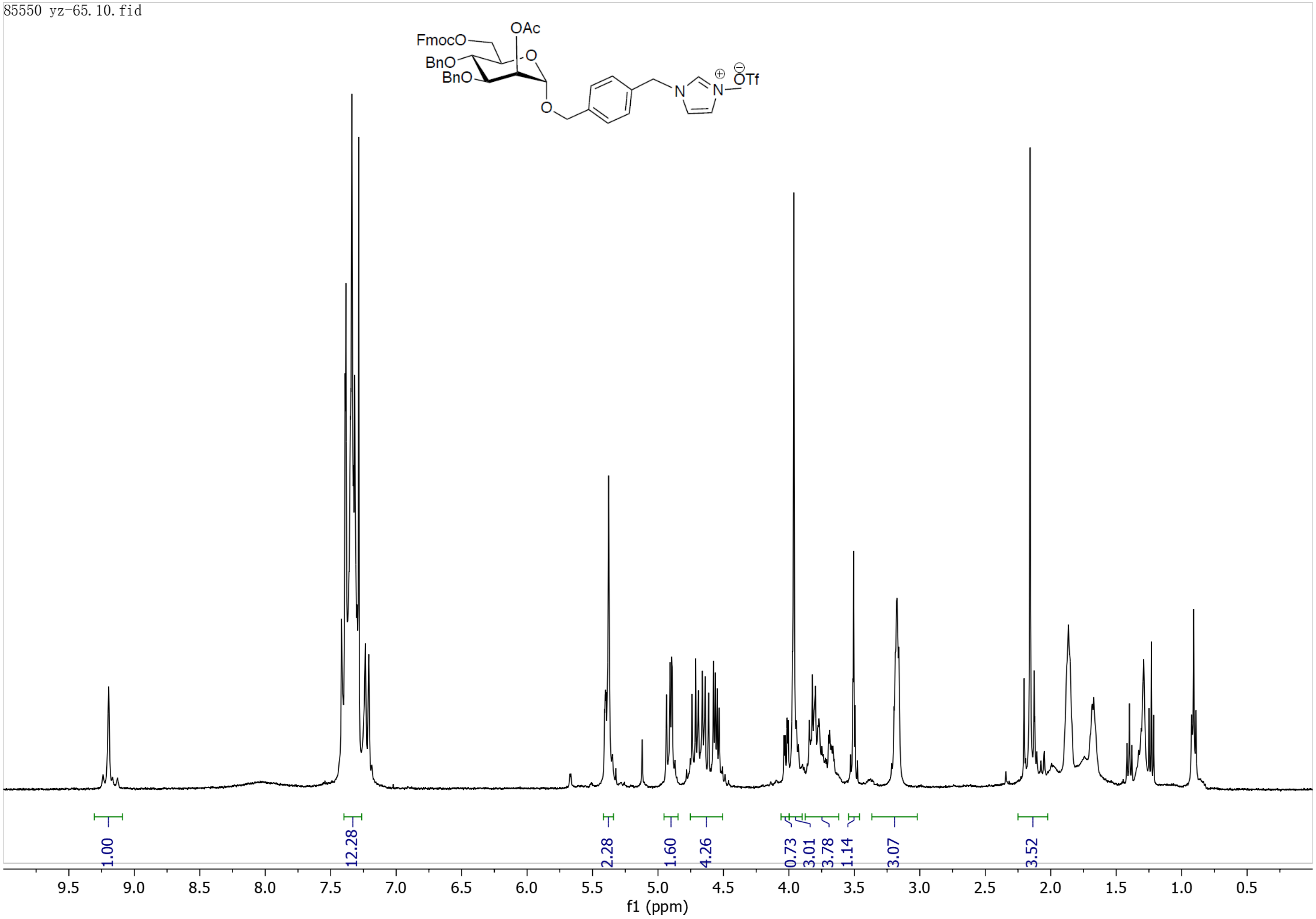

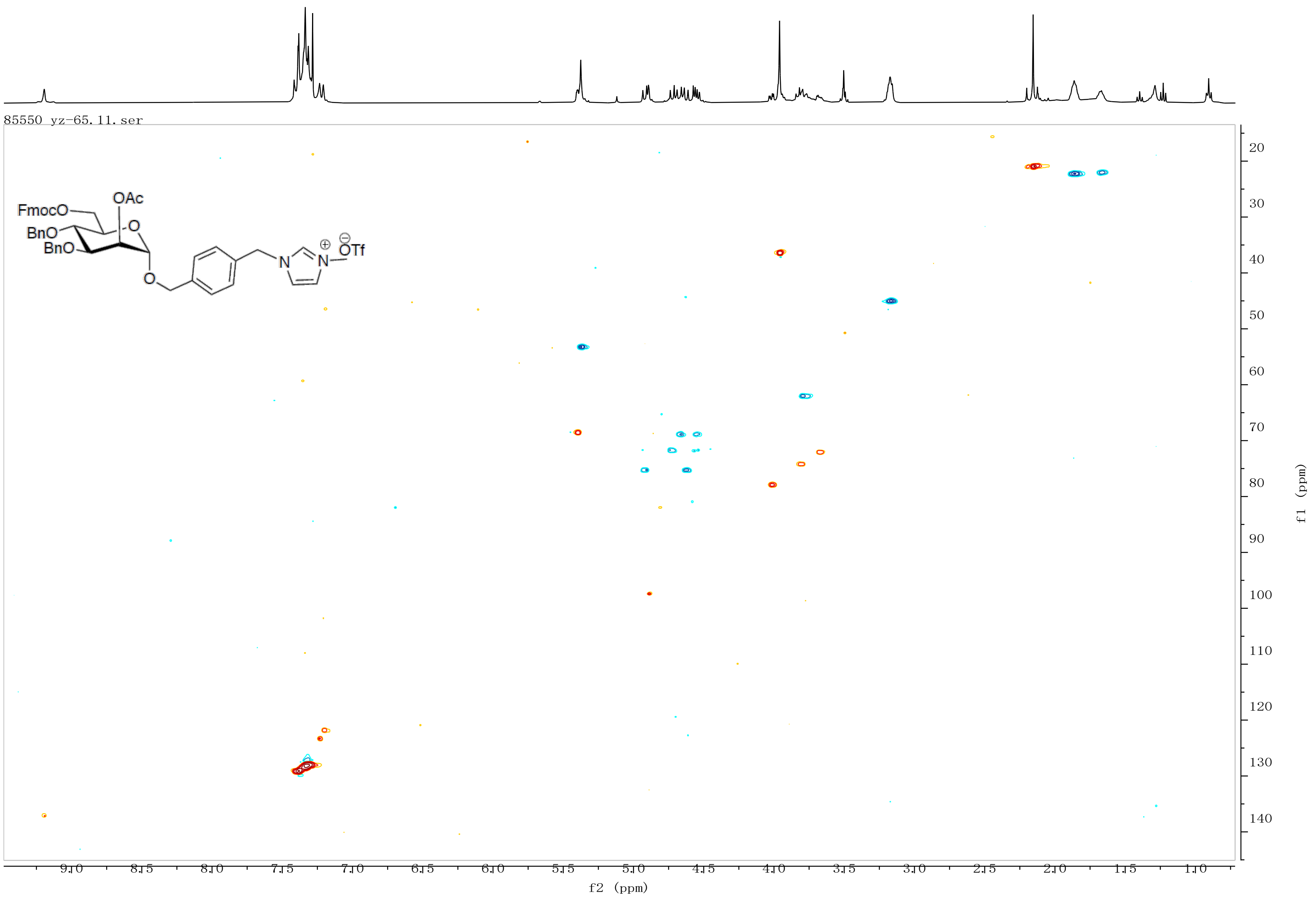
85550 yz-65.11.ser
FmocO
OAc
O
BnO
BnO
O
N
N
OTf
f2 (ppm)
f1 (ppm)

_p_-Methylphenyl 2,4-di-_O_-benzoyl-3,6-di-_O_-levulinoyl-α-D-mannopyranoside (S2)

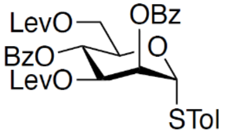

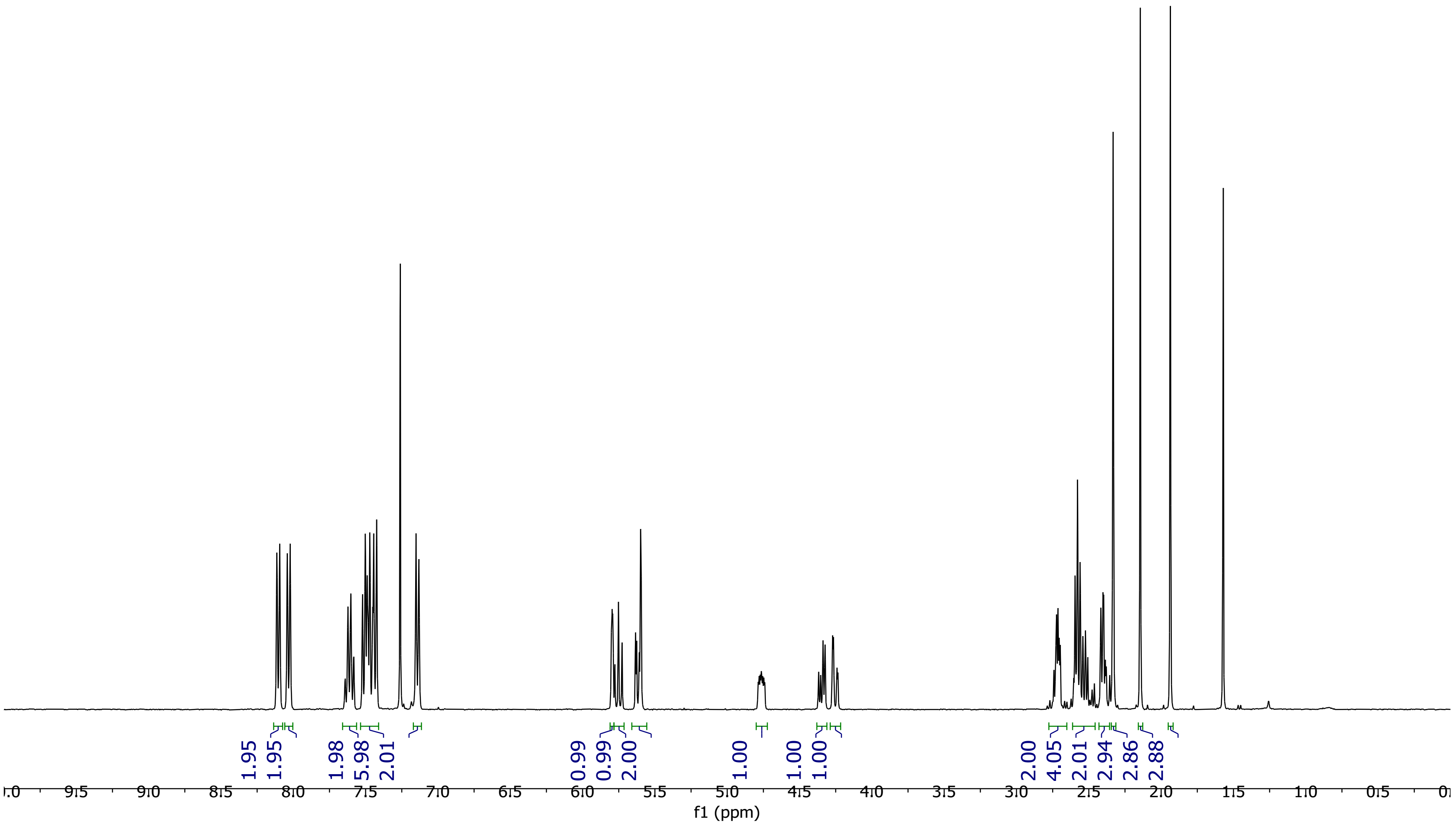

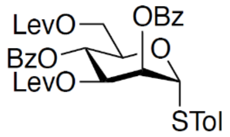
LevO
OBz
O
BzO
LevO
STol

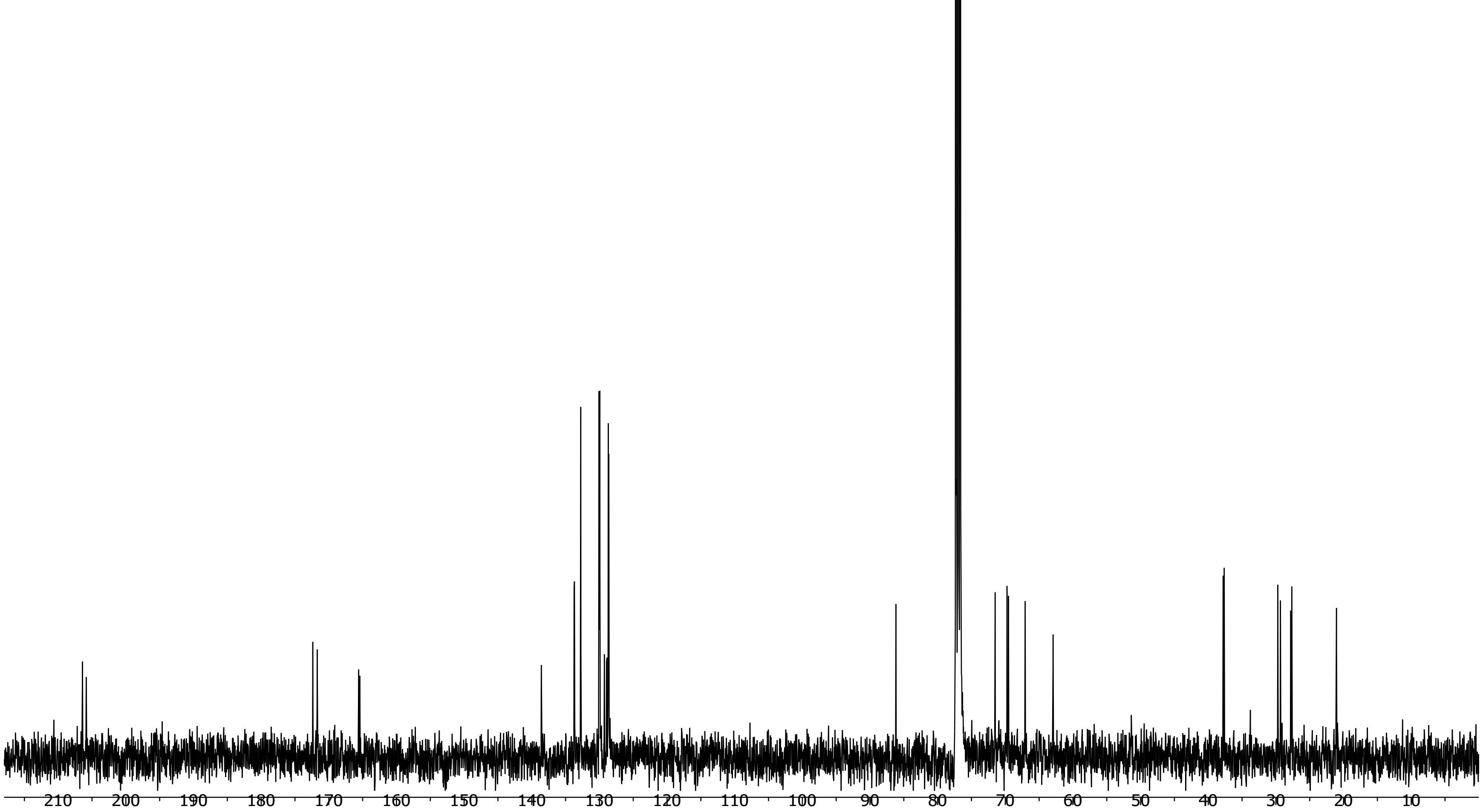
210
200
190
180
170
160
150
140
130
120
110
100
90
80
70
60
50
40
30
20
10

2,4-Di-*O*-benzoyl-3,6-di-*O*-levulinoyl-α-D-mannopyranosyl trichloroacetimidate (2b)

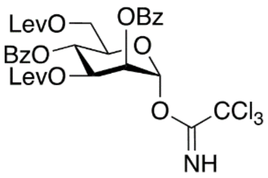

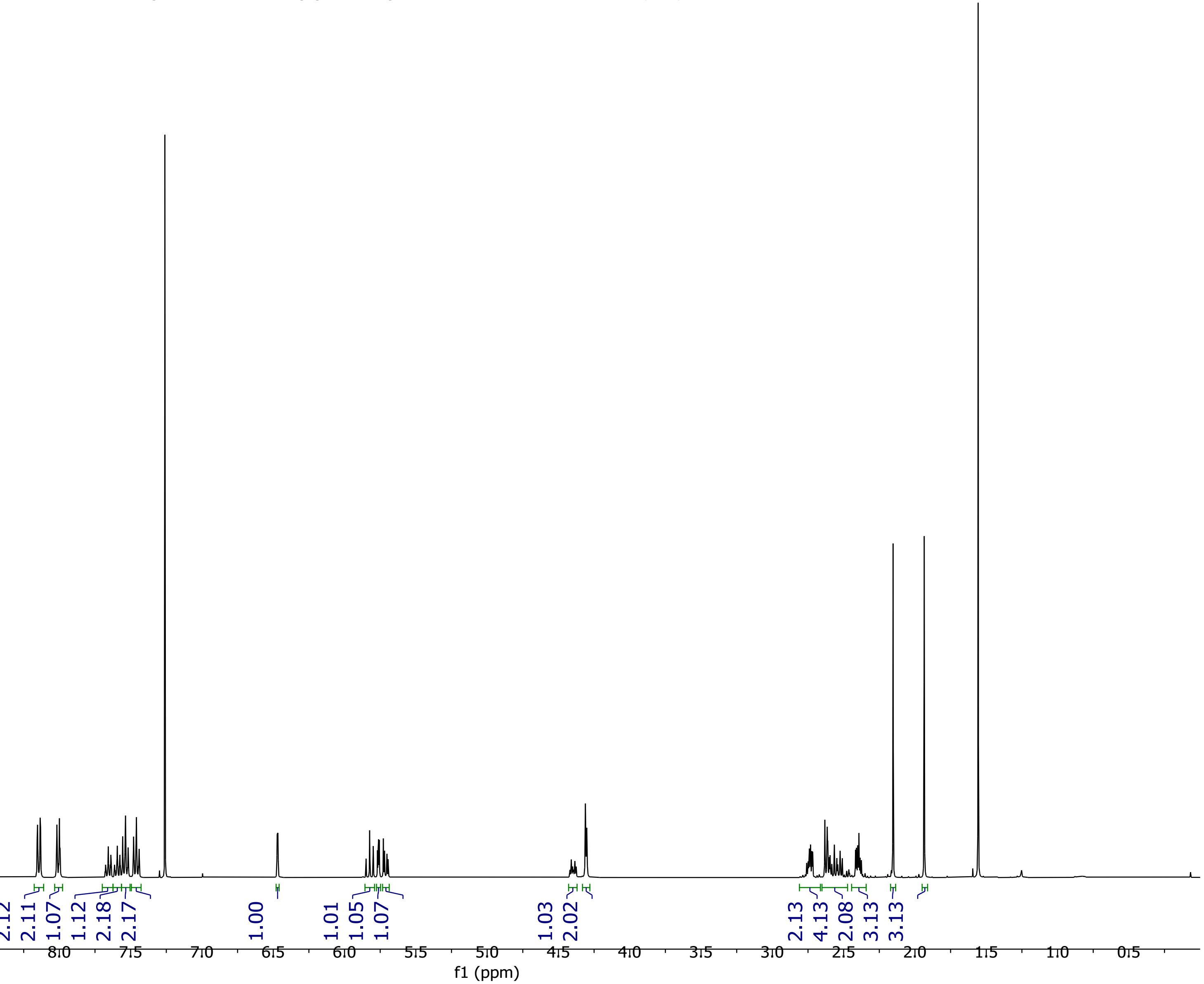

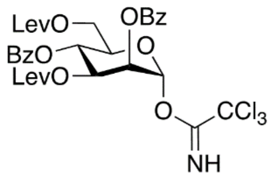

LevO
OBz
BzO
LevO
O
O
CCl3
NH

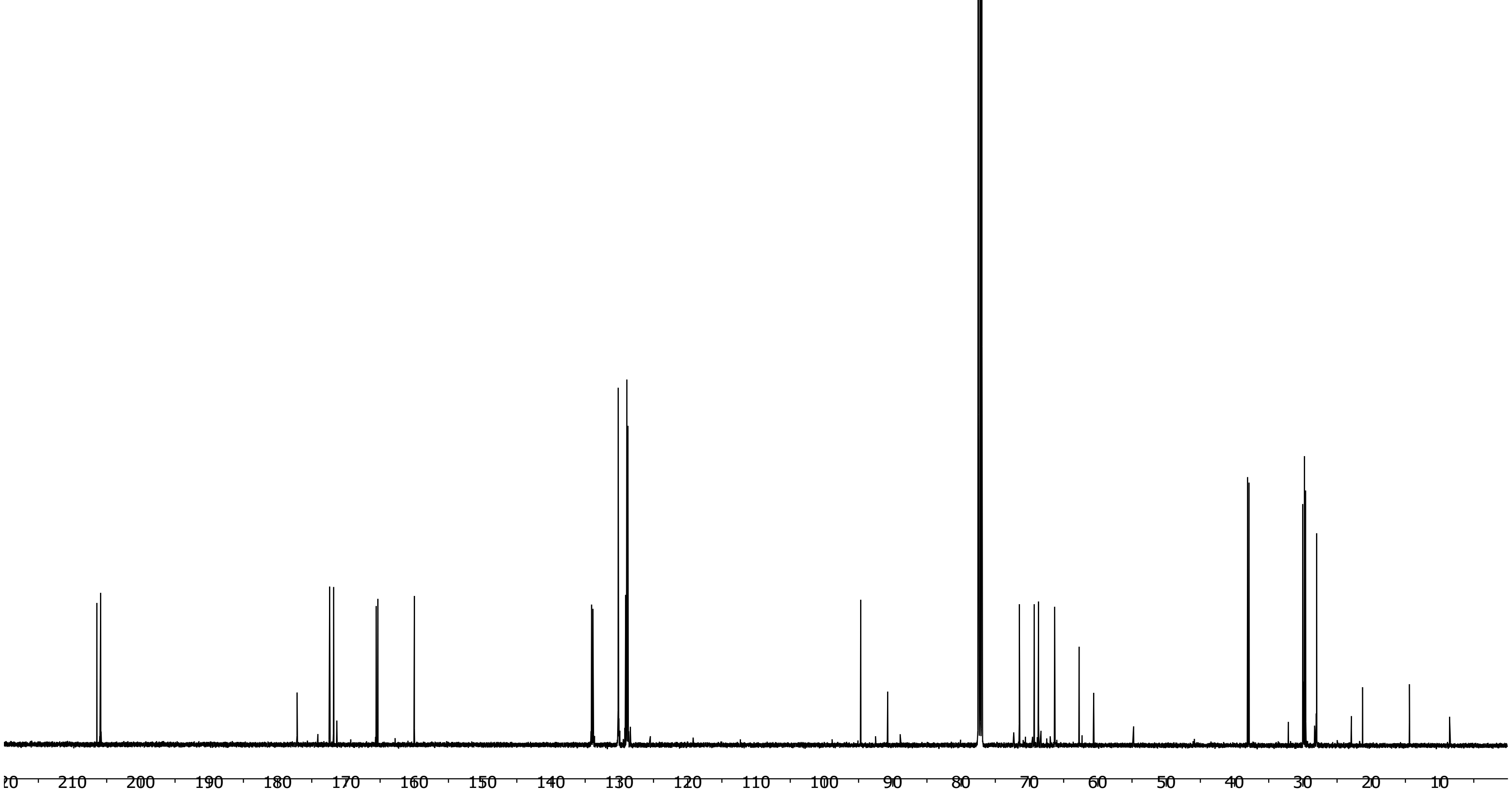

210 200 190 180 170 160 150 140 130 120 110 100 90 80 70 60 50 40 30 20 10

4-(1-Methyl-3-methyleneimidazolium)benzyl 2,4-Di-*O*-Benzoyl-3,6-di-*O*-levulonyl-β-D-mannopyranoside trifluoromethanesulfonate (6b)

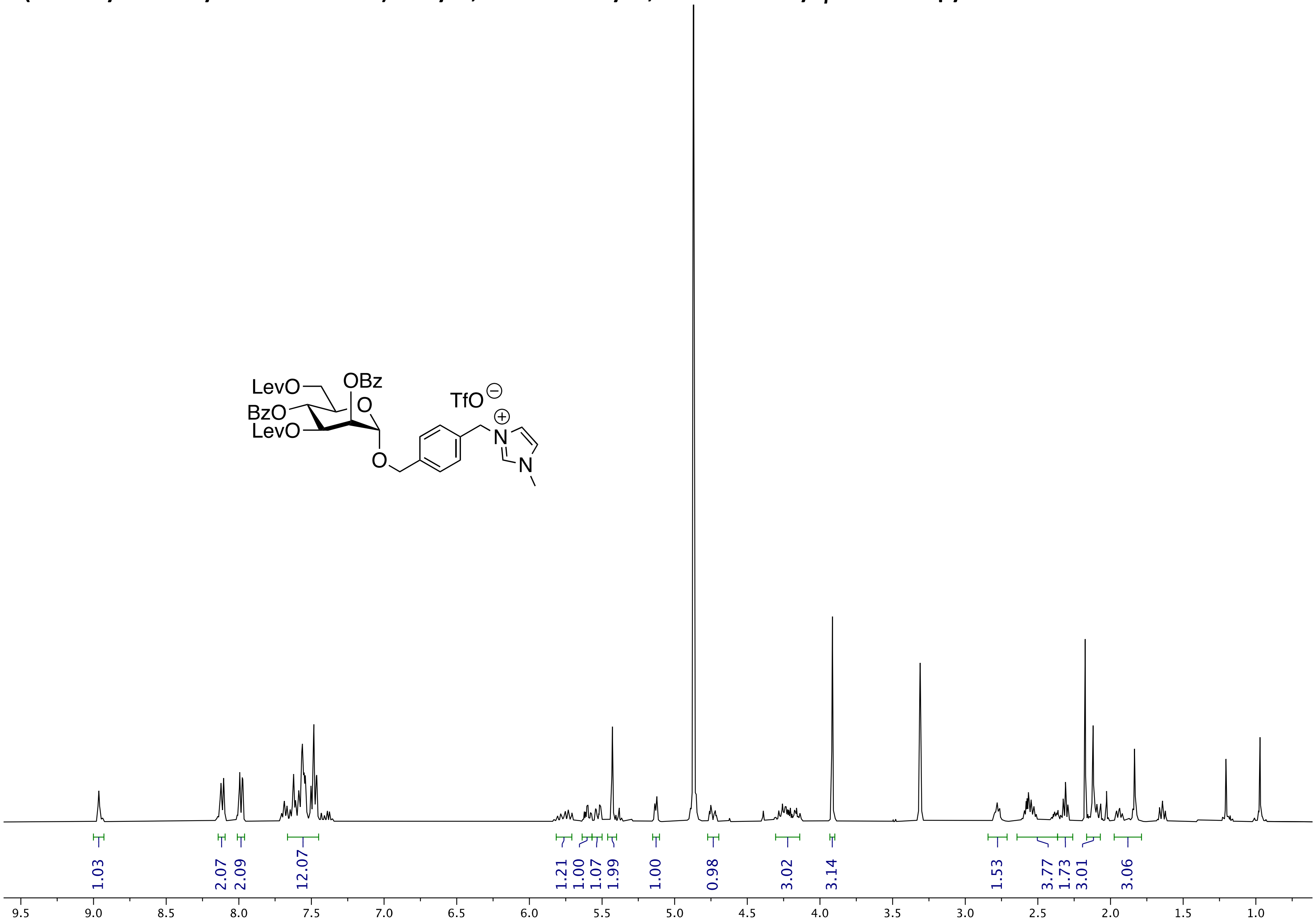

***3-(3-Methylimidazolium)-1-propyl 2,3,4-O-benzoylester-2-deoxy-2-(2,2,2-trichloroethoxycarbonylamino)-β-D-glucopyranoside trifluoromethanesulfonate* (7).**

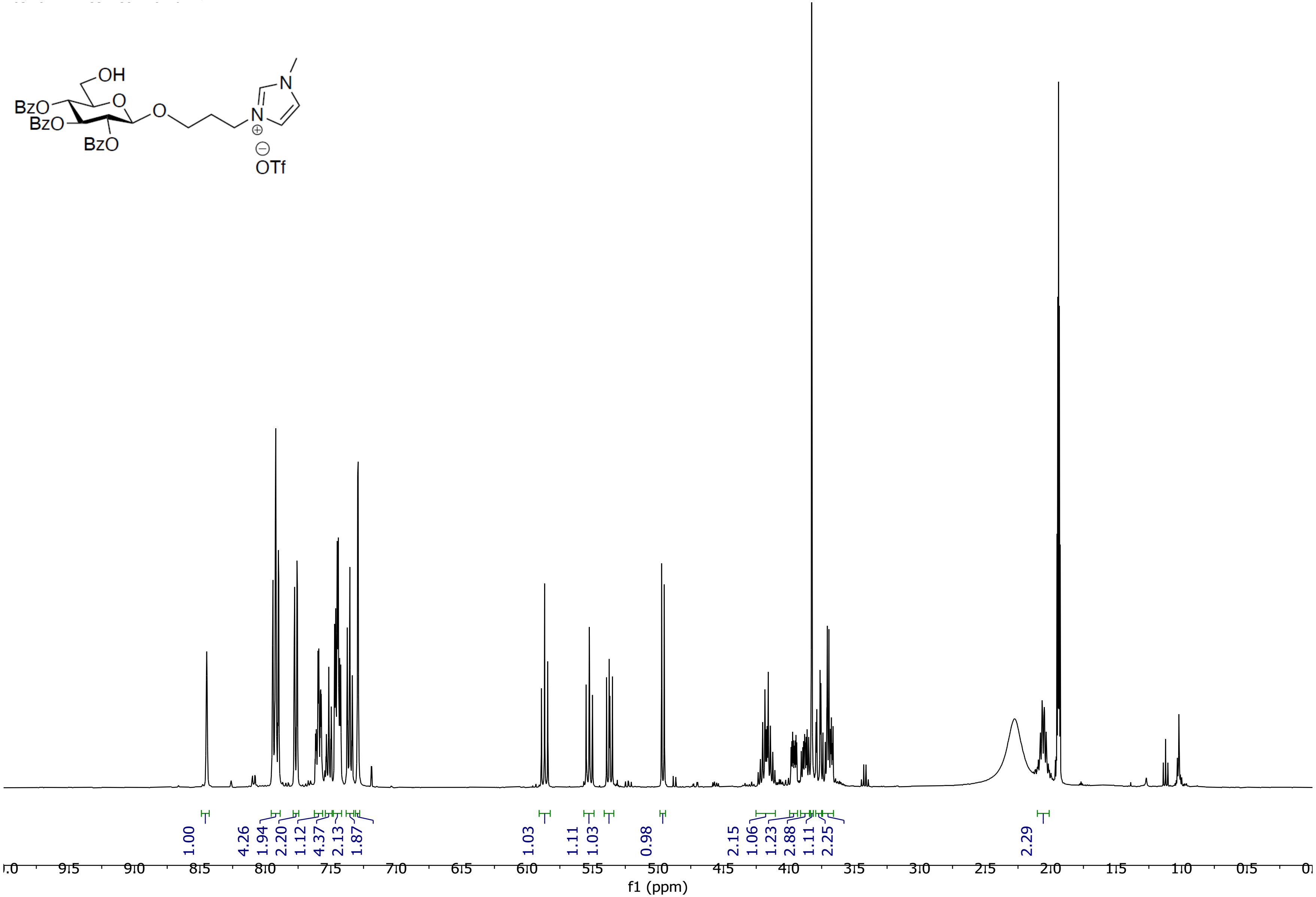

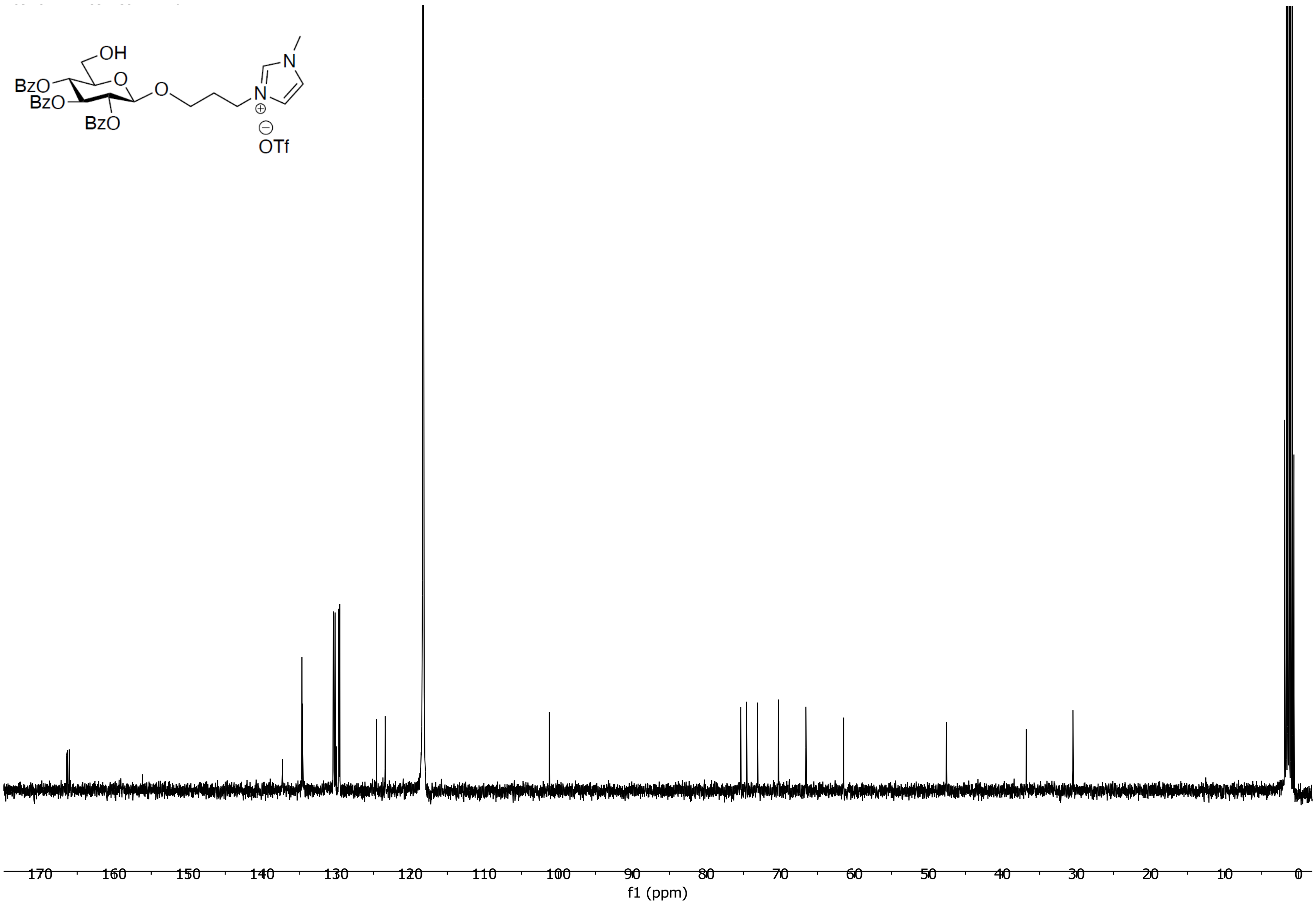

OH
BzO
BzO
BzO
O
O
N
N
OTf
170
160
150
140
130
120
110
100
90
80
70
60
50
40
30
20
10
0
f1 (ppm)

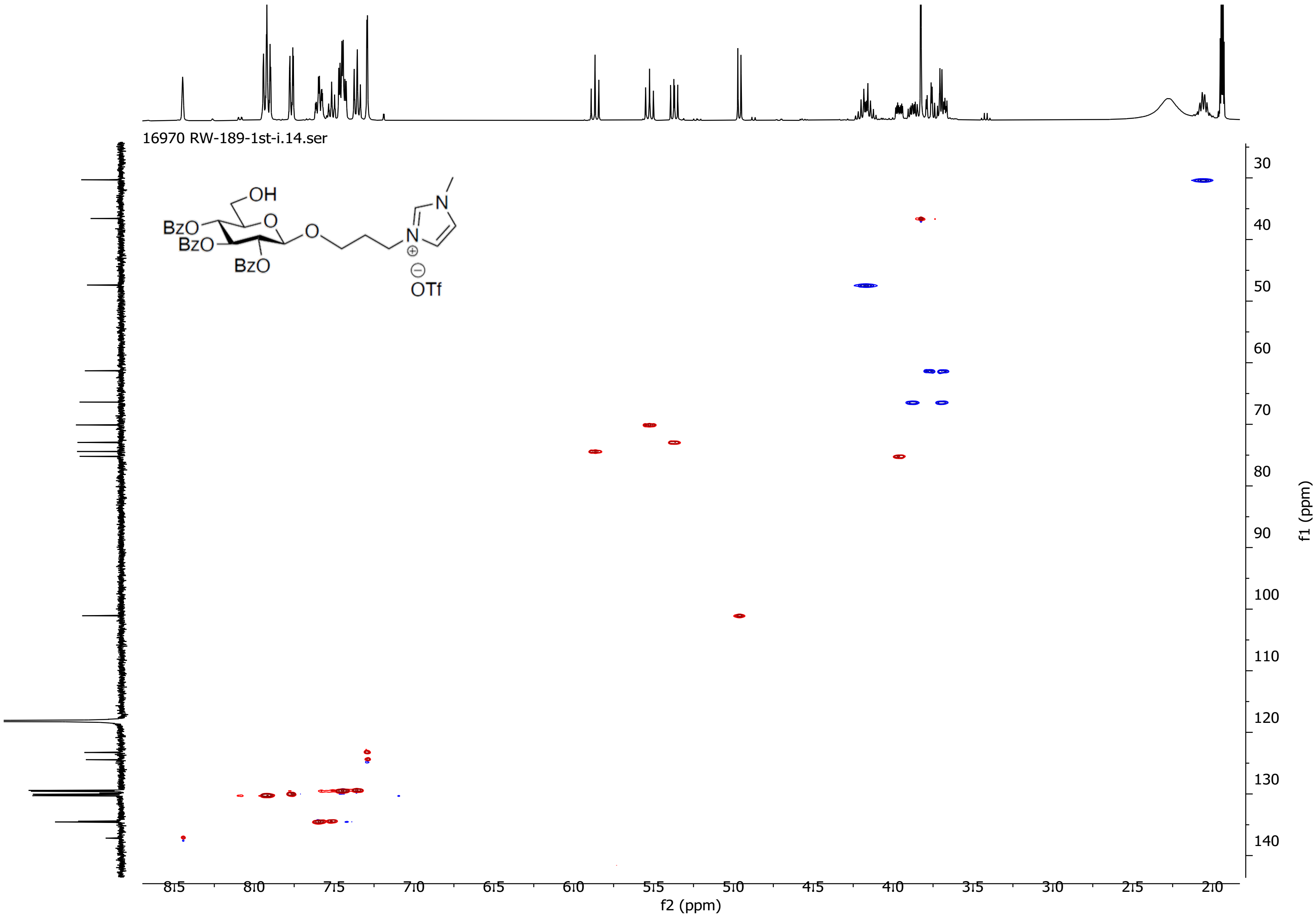
16970 RW-189-1st-i.14.ser
OH
O
BzO
BzO
BzO
O
N
N
OTf
f2 (ppm)
f1 (ppm)

3-(3-Methylimidazolium)-propyl 2,3,4-tri-O-benzoyl-6-O-(2,3,4,6-tetra-O-benzyl-D-glucopyranosyl)-β-D-glucopyranoside trifluoromethanesulfonate (8)

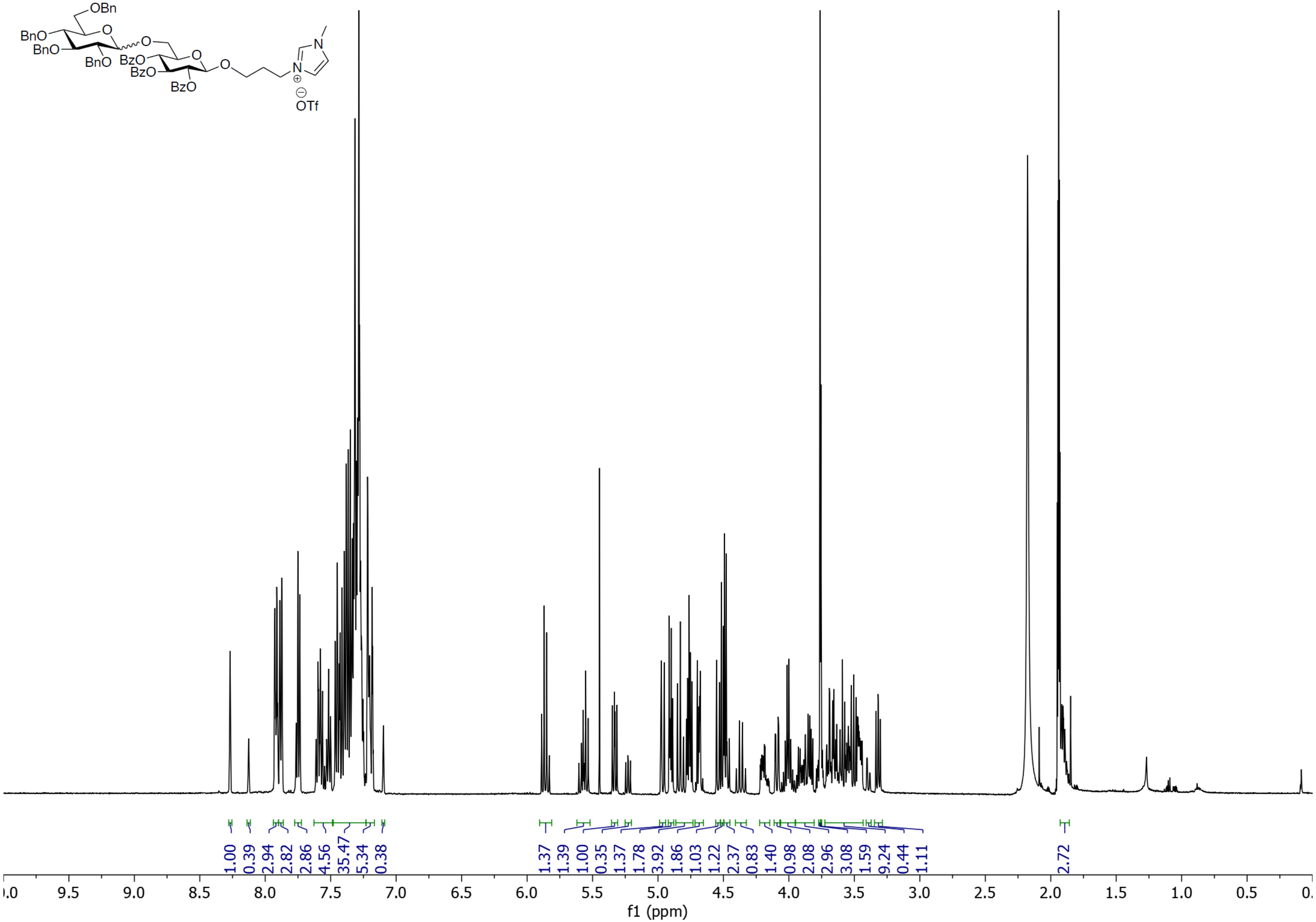

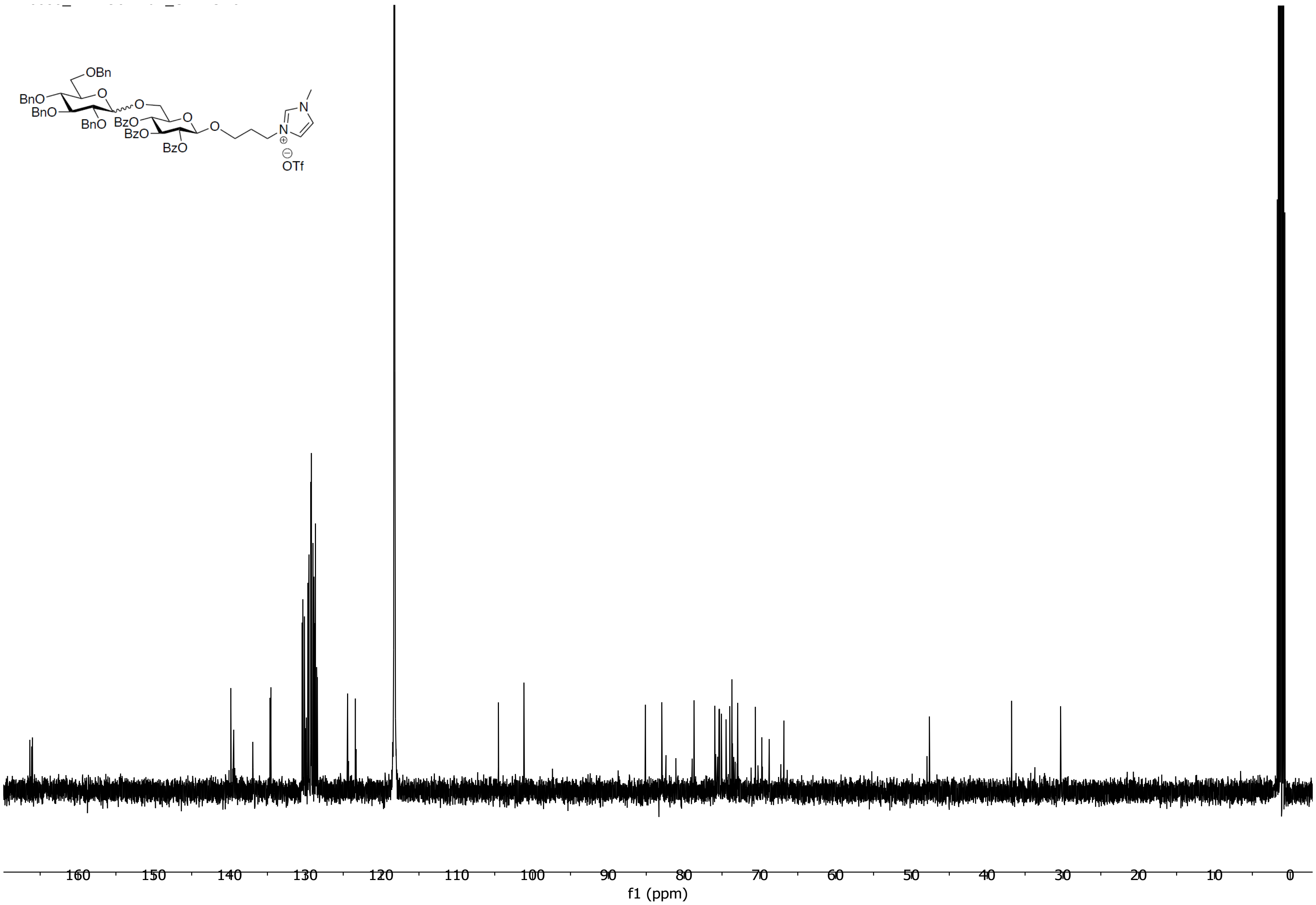
OBn
BnO
BnO
BnO
BzO
BzO
BzO
O
N
N
⊕
⊖
OTf
160
150
140
130
120
110
100
90
80
70
60
50
40
30
20
10
0
f1 (ppm)

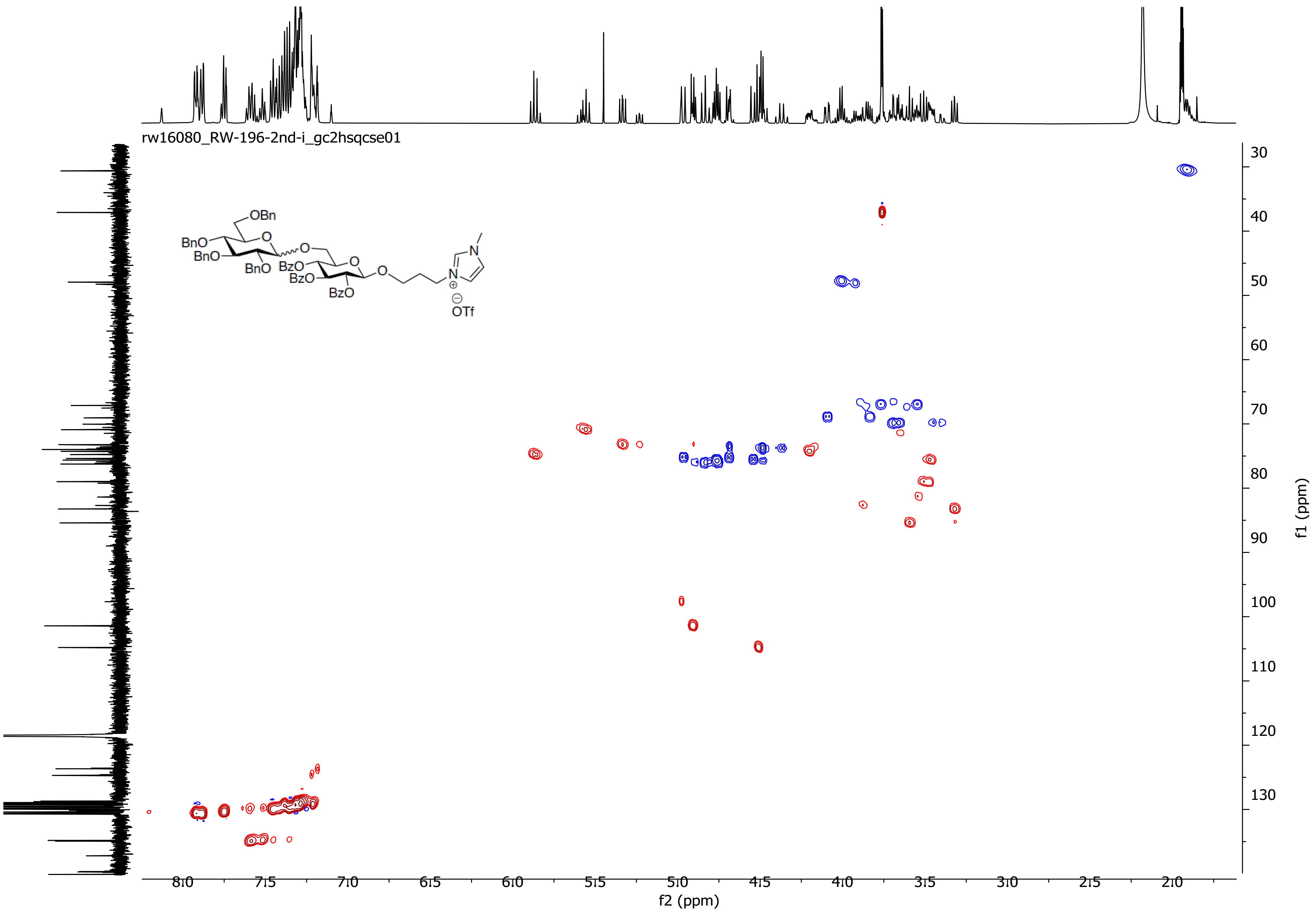
rw16080_RW-196-2nd-i_gc2hsqcse01
OBn
BnO
BnO
BnO
O
O
BzO
BzO
BzO
O
O
N
N
OTf
f2 (ppm)
f1 (ppm)
8.0
7.5
7.0
6.5
6.0
5.5
5.0
4.5
4.0
3.5
3.0
2.5
2.0
30
40
50
60
70
80
90
100
110
120
130

Trimannoside (14)

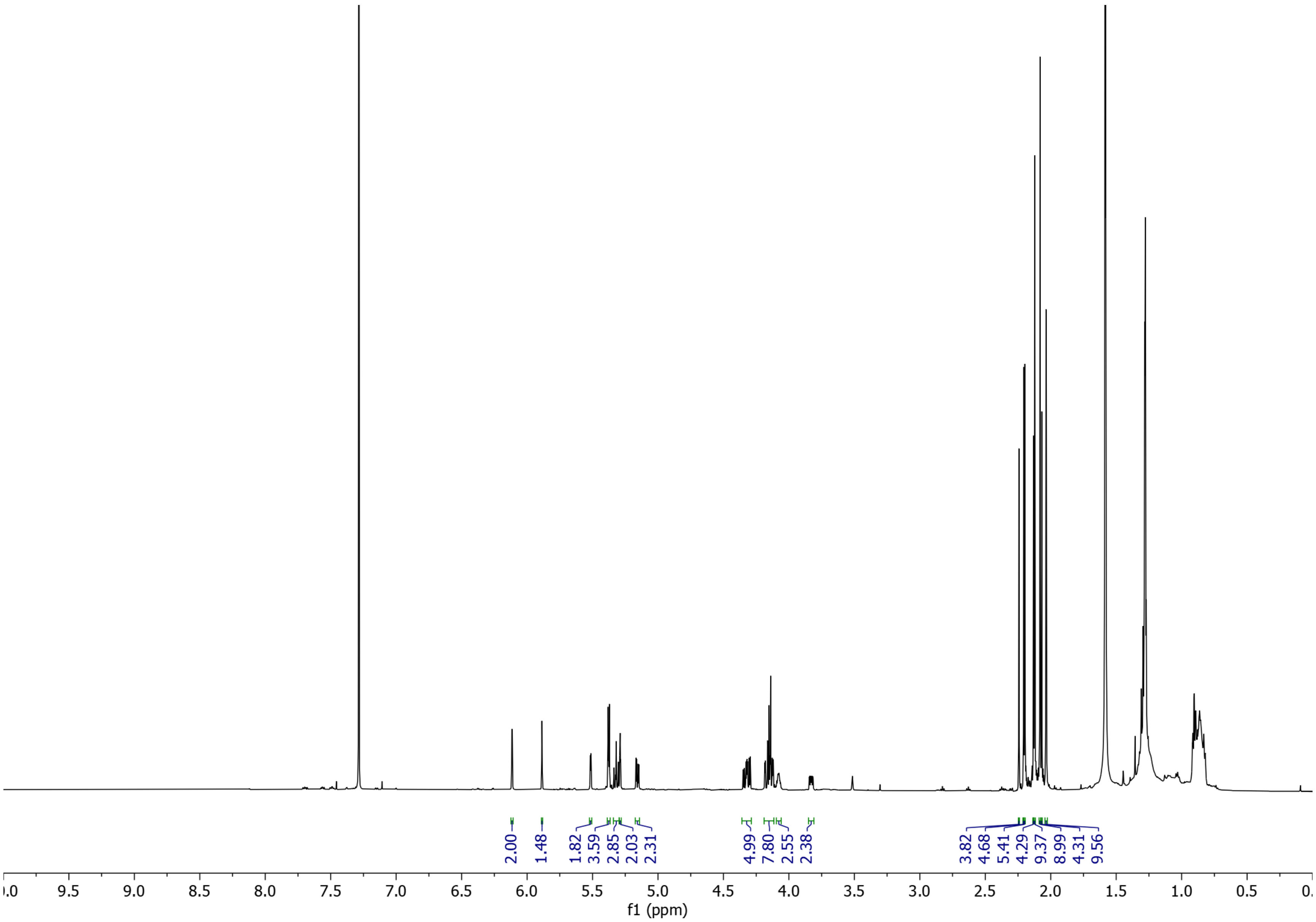

Trimannoside (14)

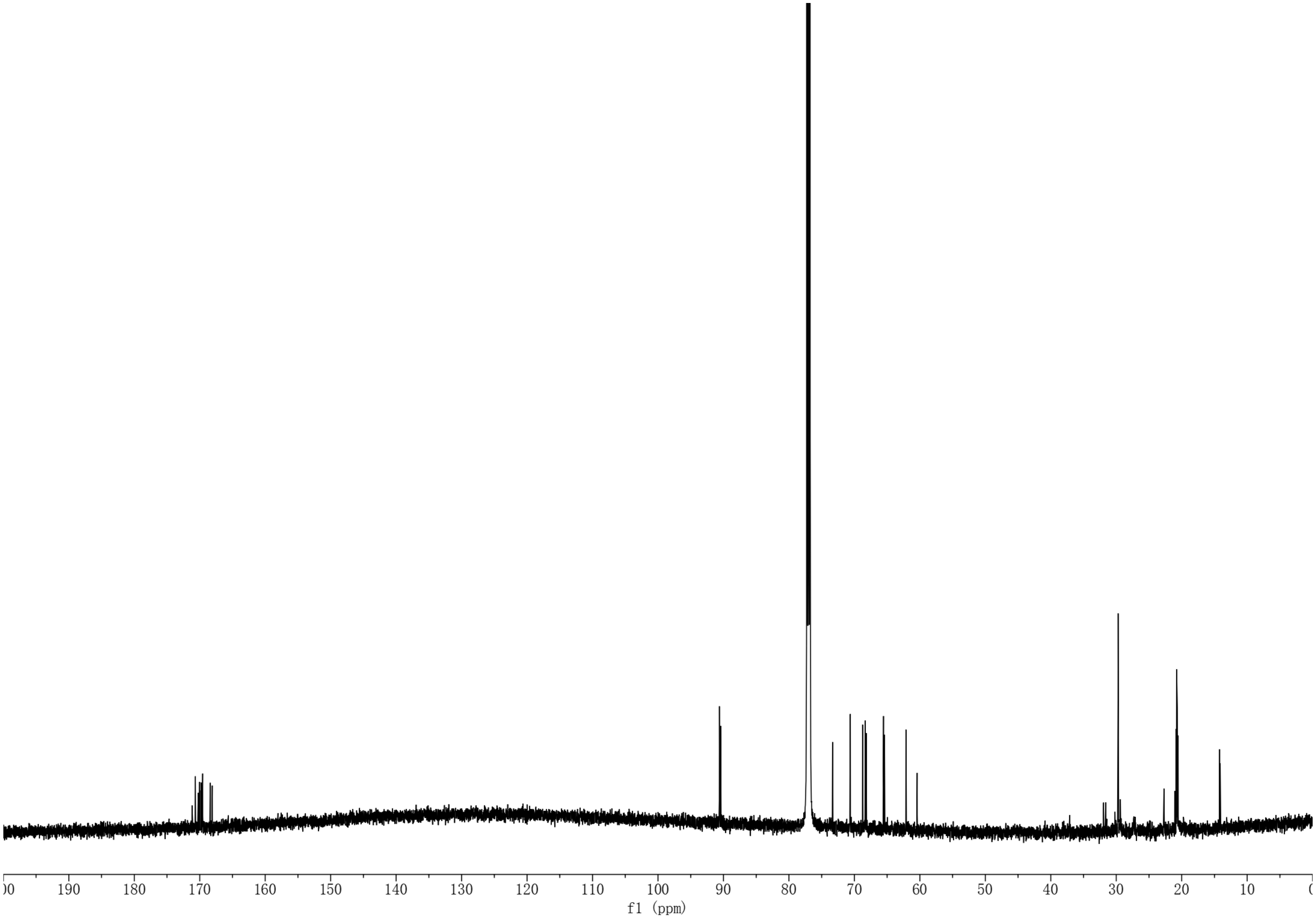

Pentamannoside (15)

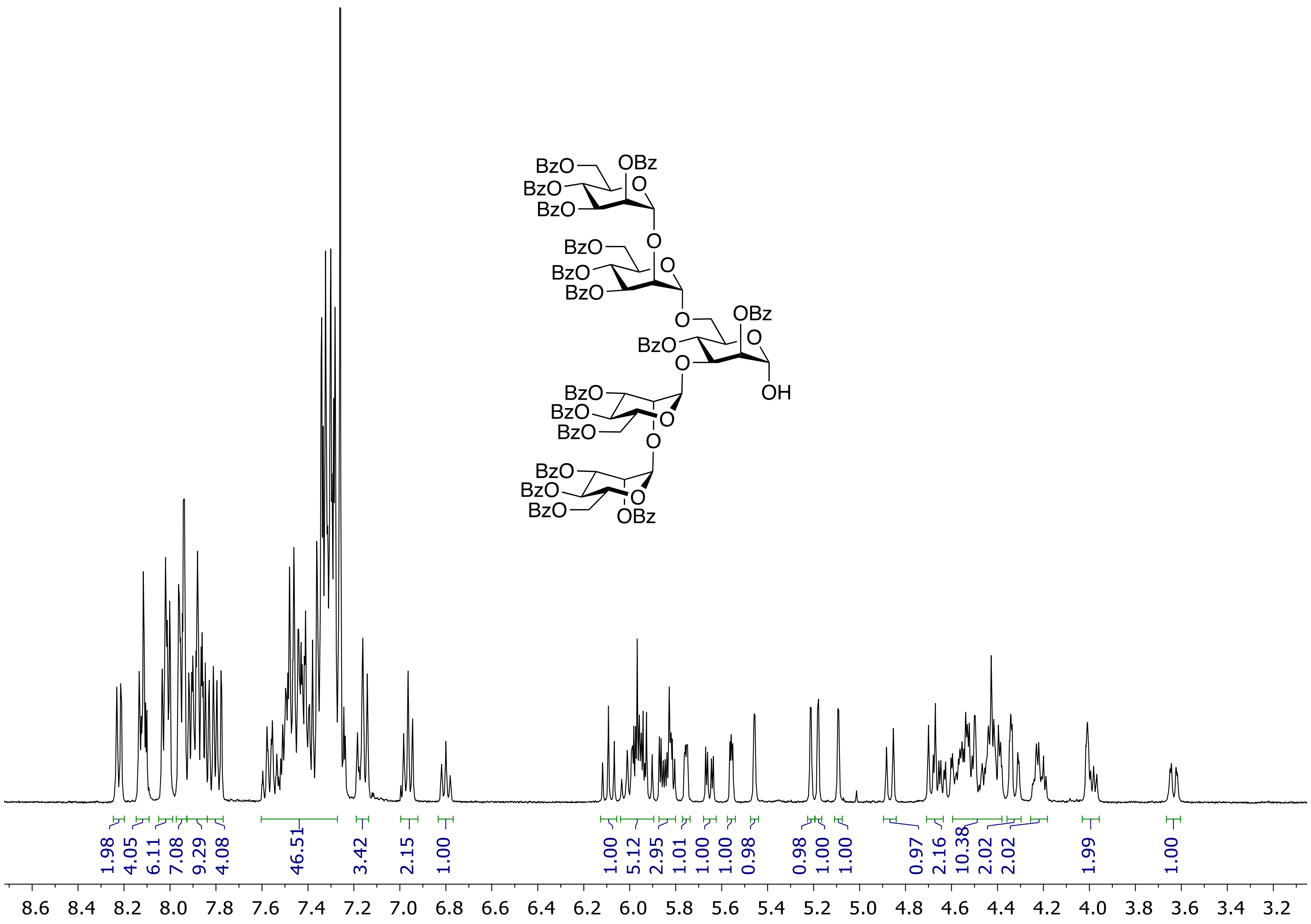

Pentamannoside (15)

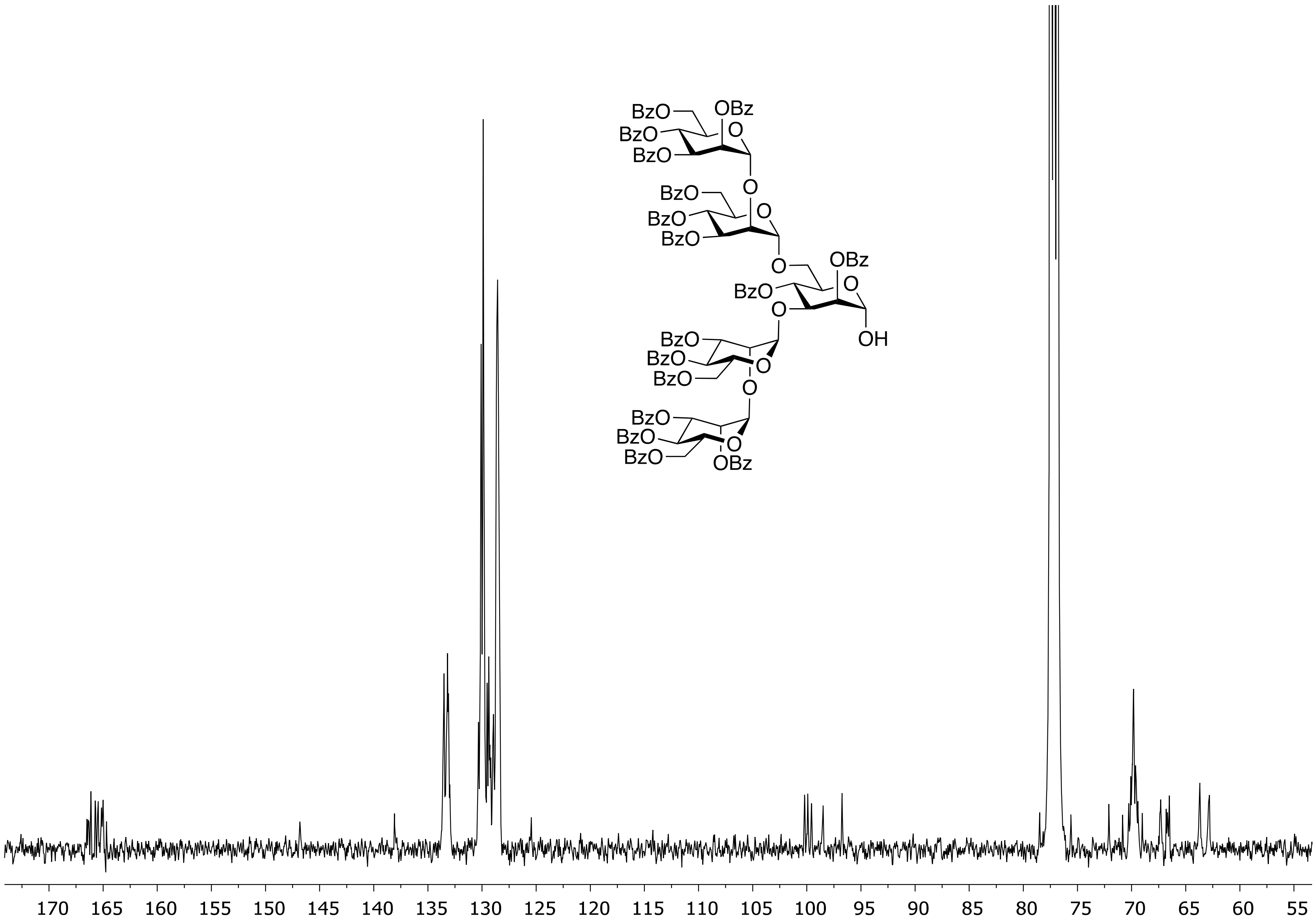

Supplement: Supplementary file 1 — au4c00686_si_001.pdf [file au4c00686_si_001.pdf]
